# Effectiveness of eHealth Nutritional Interventions for Middle-Aged and Older Adults: Systematic Review and Meta-analysis

**DOI:** 10.2196/15649

**Published:** 2021-05-17

**Authors:** Caroline Robert, Mojisola Erdt, James Lee, Yuanyuan Cao, Nurhazimah Binte Naharudin, Yin-Leng Theng

**Affiliations:** 1 Wee Kim Wee School of Communication and Information Nanyang Technological University Singapore Singapore; 2 Department of Pharmacology National University of Singapore Singapore Singapore; 3 Institute for Infocomm Research A*STAR Singapore Singapore; 4 Lee Kuan Yew Centre for Innovative Cities Singapore University of Technology and Design Singapore Singapore

**Keywords:** eHealth, mHealth, nutritional intervention, nutrition apps, middle-aged, older adults, systematic review, meta-analysis

## Abstract

**Background:**

The risk of development of chronic diseases related to poor nutrition increases with age. In the face of an aging population, it is important for health care sectors to find solutions in delivering health services efficiently and effectively to middle-aged and older adults.

**Objective:**

The aim of this systematic review and meta-analysis was to consolidate the literature that reported the effectiveness of eHealth apps in delivering nutritional interventions for middle-aged and older adults.

**Methods:**

A literature search from five databases (PubMed, CINAHL, Cochrane, Web of Science, and Global Health) from the past 5 years was performed. Studies were selected for inclusion that used eHealth to deliver nutritional interventions to adults aged 40 years and above, and reported health and behavioral outcomes. Two independent reviewers searched for research articles and assessed the eligibility of studies to be included in the review. A third reviewer resolved disagreements on study inclusion. We also assessed the quality of the included studies using the CONSORT 2010 checklist.

**Results:**

A total of 70 studies were included for analysis. The study quality ranged from 44% to 85%. The most commonly used eHealth intervention type was mobile apps (22/70, 31%). The majority of studies (62/70, 89%) provided multicomponent health interventions, which aimed to improve nutrition and other health behaviors (eg, exercise, smoking cessation, medication adherence). Meta-analysis results indicated high and significant heterogeneity; hence, conclusions based on these results should be considered with caution. Nonetheless, the results generally showed that eHealth interventions improved anthropometric and clinical outcomes, but not behavioral outcomes such as fruit and vegetable consumption.

**Conclusions:**

The use of eHealth apps to deliver health interventions has been increasing in recent years, and these apps have the potential to deliver health services to a larger group of people. Our findings showed that the effectiveness of eHealth apps to deliver health interventions for middle-aged to older adults was supported by the improvement of anthropometric and clinical outcomes. Future work could aim to develop research frameworks in administering eHealth interventions to address heterogeneity in this field of research.

## Introduction

The world is aging rapidly as people live longer and fertility rates decline. An aging population poses challenges to society as it is accompanied by a declining labor force and an increase in government spending in health care, thereby increasing the burden of health care management and delivery [1]. Moreover, the risk of developing chronic diseases such as hypertension, diabetes, and coronary heart diseases increases with age [2]. In fact, studies have shown that the risk of developing chronic diseases linked to a poor diet not only increases with age [3,4], but the onset of chronic diseases was also found to increase rapidly among the middle-aged population [5]. To delay the onset of these chronic conditions that are often related to poor diet and nutrition in middle-aged and older adults, it is important to improve diet and nutrition [6,7]. The need for an adequate, healthy, and well-balanced diet is thus essential, not only for the management of chronic diseases but also for their prevention [8,9].

To support the management of a healthy diet and lifestyle, many have turned to eHealth services. eHealth is an emerging field in health care that utilizes various technologies for the management and delivery of health services to users. eHealth encompasses mobile health (mHealth), which focuses on mobile devices and apps. eHealth technologies are invaluable in providing health care services that are personalized, timely, and efficient [10]. The use of eHealth technologies in the health care sector has been increasing in recent years [11,12]. In turn, there has been great interest in the research community for evaluating the efficacy of eHealth technologies in delivering health services and achieving positive health outcomes for users [11,13-15].

eHealth nutritional technologies typically aim to provide nutrition-related services that focus on aiding people with weight loss, in maintaining a healthy diet, and in supporting the self-management of nutrition-related chronic diseases and abating further regression of chronic conditions. Although studies have reported the success of eHealth nutritional interventions in targeting weight loss and promoting healthy eating habits [13,16-18], the study populations typically comprised mainly younger adults or adults. To our knowledge, the use of eHealth and evaluation of its effectiveness in providing nutrition-related services for older adults is much less frequently reported in the literature. A systematic review [19] did explore the use of eHealth among older adults aged 50 years and above, but with greater focus on the use of these technologies as a means for health promotion and primary prevention. The authors reported that the use of eHealth technologies was generally accepted among the older population in health promotion and primary prevention of diseases. Most of the studies reviewed involved older adults with weight problems, who used eHealth technologies that aimed to improve physical activity and diet. However, the authors did not report how successful the reviewed eHealth technologies were in improving the health of the study groups.

Therefore, the aims of this systematic review and meta-analysis were to consolidate the results of published research studies on the use of eHealth technologies for nutrition- and diet-related services for middle-aged and older adults. The onset of chronic diseases related to poor lifestyle habits increases with age [20], which could lead to higher stress and burden on health care systems in the face of an aging society [21]. Furthermore, eHealth was suggested to be advantageous in administering health care services to older adults owing to its efficiency and cost-effectiveness, but more research is needed to evaluate the effectiveness of eHealth on improving or maintaining health, which can strengthen evidence to support its use in administering health services in an effective and cost-effective manner [22,23]. Thus, the purpose of this systematic review and meta-analysis was to evaluate the effectiveness of eHealth nutritional interventions for the prevention and management of chronic diseases among middle-aged and older adults.

To address this aim, we established the following research questions: (1) Which eHealth technologies (eg, mobile phone, wearables) are most commonly used in eHealth nutritional interventions for middle-aged and older adults? (2) What are the common types of eHealth features provided by eHealth nutritional technologies? (3) Compared to non-eHealth interventions/standard care, does eHealth result in improvements to health and behavioral outcomes related to nutrition and diet? (4) Does the duration of eHealth interventions lead to better overall improvements in health and behavioral outcomes related to nutrition?

We define eHealth nutritional interventions as those using technologies such as mobile devices, telephones, wearables, sensors, and mobile and web-based apps to support users in achieving nutritional-related outcomes such as weight reduction, or changes in dietary intake or behavior. This support could be in the form of a reminder system, coaching calls, sharing of educational content, or sending motivational messages with the primary aim to support users in various activities such as setting and achieving nutritional health goals, recording dietary behavior, monitoring food intake, regulating eating habits, or tracking physical activity.

We performed a systematic review to consolidate the literature on the use of eHealth technologies in providing nutrition-related services to answer research questions 1 and 2. Additionally, meta-analyses were performed to evaluate the effectiveness of eHealth nutritional interventions in improving outcomes, addressing research questions 3 and 4. The strengths and limitations of employing such eHealth nutritional interventions in older adults are also discussed to provide considerations for future research in developing and implementing eHealth nutritional technologies.

## Methods

### Literature Search

This systematic review was performed in accordance with the Preferred Reporting Items for Systematic Reviews and Meta-Analyses for Protocols 2015 (PRISMA-P 2015) guidelines [24]. Systematic searches were performed in May 2020 using five databases: PubMed/Medline, CINAHL, Cochrane, Web of Science, and Global Health. We considered that these databases are sufficiently extensive to cover the literature on eHealth, mHealth, public health, and health systems. In addition, manual searches for relevant studies were also performed from the reference lists of retrieved articles and directly in Google Scholar. Systematic searches were limited to the English language literature, human research, and year of publication between 2014 and 2019. We limited the search to articles published in the last 5 instead of 10 years, as technology is rapidly outdated and findings published more than 5 years ago might not be up to date or of high relevance to the current and future context of eHealth nutritional interventions. We identified the search categories based on the purpose of the systematic review. The search term “eHealth” was selected as it is the type of intervention of interest to our research questions, “nutrition” was selected since the purpose of the eHealth interventions of interest is to improve nutrition, and the search terms “middle-aged” and “elderly” were selected as these were our target populations. These search terms were entered in the Cochrane Library medical subject heading (MeSH) browser, and the relevant MeSH terms and synonyms were selected. MeSH terms represent a controlled vocabulary thesaurus maintained by the National Library of Medicine, which are used to index research articles for the PubMed and Medline databases. As shown in Table 1, the selected MeSH terms were used to perform full-text literature searches in the respective databases. Additionally, we used Boolean operators (eg, AND, OR, NOT) in our search strategy to provide a narrower and more productive search. More detailed information about the search strategies used for each database are presented in Multimedia Appendix 1.

**Table 1:** Medical subject heading (MeSH) search terms used for the literature search.

Search terms	MeSH terms
eHealth	eHealth OR telemedicine OR telehealth OR mhealth OR mobile health
Nutrition	Nutrition, diet, food, eating, food intake, ingestion, diet habit
Middle-aged and elderly	middle aged OR aged OR “aged, 80 and over” OR elderly

### Inclusion Criteria and Selection of Studies

Studies were selected to be included in the systematic review based on the following inclusion criteria: (1) used a form of eHealth nutritional intervention for disease prevention and/or management; (2) involved adults aged 40 years and above; and (3) reported data regarding anthropometric measures (eg, weight, BMI, blood pressure readings), dietary behaviors, and other health outcomes (eg, self-report of physical and mental well-being).

Based on our definition, an eHealth nutritional intervention should encompass technologies that aid participants in losing weight, in maintaining a healthy diet, and in supporting the self-management of nutrition-related chronic diseases and abating further regression of chronic conditions. The use of eHealth to improve diet and nutrition among older adults could either be the primary or secondary aim of the intervention. Likewise, the intervention could be a stand-alone nutrition-only intervention or part of a multicomponent intervention such as those that aimed to improve other health behaviors (eg, physical activity).

We compiled a comprehensive list of outcome measures from the literature pertaining to nutrition, diet, and health outcomes that we could expect to be reported as the results of eHealth interventions, including: (1) anthropometric outcomes such as weight, BMI, waist circumference, hip circumference, body adiposity, and waist-to-hip ratio; (2) clinical outcomes such as liver enzyme and lipid profile, cholesterol level, blood pressure level, fasting blood glucose level, hemoglobin A_1c_ (HbA_1c_) level, urinary sodium level, triglycerides level, fat mass or body fat, pulse pressure, insulin level, C-reactive protein, alkaline phosphatase, total white blood cell, alanine aminotransferase/aspartate aminotransferase, and serum creatinine; (3) behavioral outcomes such as dietary attitudes, dietary behavior, adherence to a Mediterranean diet, adherence to health behaviors, physical activity level, nutritional status, low salt content of purchased foods, low saturated fat and energy content of purchased foods, and the Framingham Heart Study cardiovascular disease (CVD) risk score [25,26]; (4) educational outcomes such as nutritional knowledge and health literacy; and (5) other outcomes such as psychological outcomes, quality of life, and app usage.

A study was excluded from the review if it (1) implemented a nonexperimental study design (eg, observational and case studies, study protocol); (2) was not a peer-reviewed research article (eg, conference proceedings, letters, commentaries); (3) did not report any health outcomes as aforementioned in the inclusion criteria; and (4) used an eHealth intervention as a follow-up intervention to observe maintenance of outcome changes from a previously administered health intervention that did not use eHealth.

Titles and abstracts of studies were first screened by an independent reviewer. During the first round of screening, studies that did not meet the eligibility criteria for selection were excluded from the review. Two independent researchers reviewed and screened the remaining studies based on the inclusion and exclusion criteria, and any disagreements were discussed and resolved with a third independent reviewer. Data and references were managed using EndNote software.

### Data Extraction

The process of data extraction followed a standardized procedure as reported in previous systematic reviews [13,19,27]. This systematic review adhered to the guidelines proposed by Cochrane Handbook for Systematic Review of Interventions [28]. Data were extracted based on our research aims, and the inclusion and exclusion criteria. Multimedia Appendix 2 presents the relevant characteristics and data that were extracted from the included studies. With the aim to answer our research questions, we extracted data on the study design, participant information, intervention description, outcome measures, and results. Study design comprised the study method or design, information regarding the groups involved in the study, duration of the intervention, measurement time points, and attrition rate. Participant information included sample size and sample selection criteria such as demographics, disease or health condition, mean age, and gender distribution. Interventions are described as a brief overview of the intervention and eHealth features. Outcome measures included the primary and secondary outcomes measured in the study. Results are described as an overview of the main primary and secondary findings of the study, including details on mean or percentage changes in outcomes, as well as significance levels where possible.

### Quality Assessment

The quality of all included studies in the systematic review was assessed using the CONSORT (Consolidated Standards of Reporting Trials) 2010 checklist for reporting randomized controlled trials (RCTs) [29]. The CONSORT checklist includes a 25-point evaluation for RCTs. Although it is mostly used to assess RCTs, most of the criteria in the CONSORT checklist are applicable to other study designs. Quality assessment using the CONSORT checklist has also been adopted in other systematic reviews [13,27]. To assess quality, points were allocated to each criterion: 1 point was given to a fulfilled criterion, 0.5 points to a partially fulfilled criterion, 0 points to an unfulfilled criterion, and NA was indicated for criteria that were not applicable to the study. The quality of the systematic review was checked in accordance with the PRISMA-P 2015 checklist and reported in Multimedia Appendix 3.

### Meta-analysis

A meta-analysis was performed using Cochrane Review Manager version 5.3 [30]. Only studies with an RCT design were included in the meta-analysis, as scores at baseline and postintervention are not independent [31]. Mean change scores from baseline to postintervention were included as the dataset for analysis. Postintervention results were treated as the scores or values collected at the end of the intervention period. SD values of the mean change scores were obtained as reported or calculated from the standard error or 95% CI if the study did not report the SD values [28]. Studies were excluded from the meta-analysis if they did not provide sufficient data for computing the mean change and SD values, or if the methodology and instruments used to measure the outcomes were not standardized and similar to the majority of studies in the meta-analysis. Our meta-analysis was performed using a random-effects model, with mean difference for outcomes that were measured and reported in a standardized manner (eg, weight in kilograms), and standardized mean difference for outcomes that were measured using different scales or measurements [26]. Publication bias for each outcome was assessed with funnel plots (Multimedia Appendix 4). Subgroup analyses based on the duration of the intervention period (ie, ≤3 months, 4-6 months, 7-12 months) were performed on outcomes that had at least three studies per subgroup to evaluate whether the intervention duration had differential effects on the outcomes.

## Results

### Study Selection

**Figure 1:**
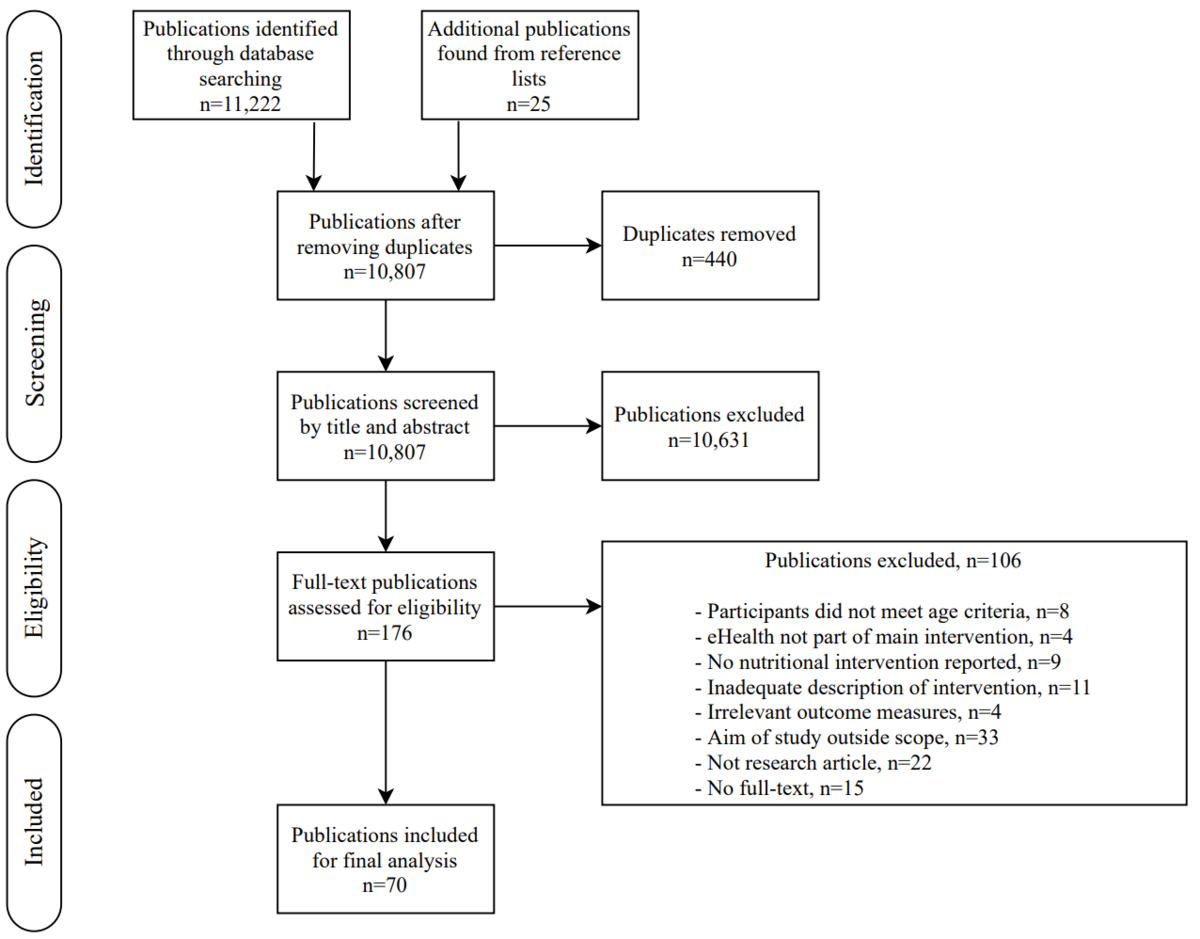
Flowchart of publication selection procedure.

Figure 1 presents the flowchart of the study selection process. A total of 11,247 studies were identified from database searches and additional reference lists. After the removal of 440 duplicates, 10,807 studies were screened by title and abstract. Among these, 176 full-text publications were considered to be potentially eligible for inclusion. We had several reasons for the exclusion of publications. Studies whose recruited participants were aged 39 years and below were excluded as they did not meet the age criterion of our target population. Publications were excluded that did not focus on eHealth as part of their main intervention. Publications were excluded that reported an eHealth intervention, but not related to nutrition. Publications that did not describe what the eHealth devices aimed to achieve in the nutritional intervention (eg, whether they comprised an educational component) were excluded due to the inadequate description of the intervention. Publications were also excluded for studies that did not measure any eHealth nutritional outcomes. Some publications were excluded because the study did not aim to administer an eHealth nutritional intervention to improve health outcomes of the participants but rather to, for example, compare methodologies or to evaluate the cost-effectiveness of eHealth programs, and were thus outside of the scope of this analysis. We only included full-text research articles. Of the full-text articles identified, 70 studies were finally included in the systematic review, having fulfilled our study inclusion criteria and having clearly indicated the effectiveness of implementing eHealth nutritional interventions on nutrition-related health outcomes.

### Study Characteristics

The characteristics of the included studies for the systematic review are presented in Multimedia Appendix 2. This systematic review adheres to the Cochrane guidelines for reporting characteristics of studies that were included in the review [28]. Thirteen studies were performed in the Asia-Pacific region [32-44], 3 studies were performed in the Middle East [45-47], 35 studies were performed in North America [48-82], 1 study was performed in South America [83], and 18 studies were performed in Europe [84-101]. The majority of the studies included were RCTs, with 38 studies that performed two-group RCTs [33-36,38-41,43,44,47-51,53,55,57-60,69,70,74-77,80,83, 85,88,92,93,95-97,100,101] and 12 studies that performed three-group RCTs [45,46,54,56,61,79,86,87,90,91,94,99]. Nineteen studies involved a pre-post study design [32,37,42,52,63-68,71-73,78,81,82,84,89,98] and one study involved a pragmatic trial design [62].

### Participant Information

The most common target population group was patients with chronic diseases. Of the 38 studies that recruited patients with chronic diseases, 25 recruited patients with diabetes mellitus or who were in a prediabetic state [32,40-44,46,47,50-52, 55,60,62,65,66,70-72,75,82,83,86,90,93], 7 studies recruited patients with CVD [33,34,36,39,45,49,53], 1 study recruited patients with liver disease [48], 2 studies recruited patients with hypertension [61,69], and 3 studies recruited patients who reported any form of chronic disease [38,94,95]. Seventeen studies recruited patients with other clinical disorders such as individuals at risk of breast cancer [58], cancer survivors [64,92], those with obesity [57,59,63,67,68,73,76,77,89,101], and those who had obesity along with other health conditions [56,74,91,100]. The remaining 14 studies recruited healthy older adults [35,37,54,78-80,84,85,87,88,96-99]. With respect to gender, 47 studies recruited more female participants [32,34,37,44,48,52,54-59,62-66,68-72,76-78,80-87,89,91-93,95-97,99-101], 5 of which recruited only female participants [56,58,63,64,79]. One study recruited equal numbers of male and female participants [42]. The remaining 22 studies recruited more male participants for their intervention [33,35-41,43,45-47,49-51, 53,60,61,67,74,90,94], 3 of which recruited only male participants [35,37,74]. Most of the studies (38/70, 54%) included participants with mean ages ranging from 50 to 59 years [32,33,39-43,45,46,48,51,53-55,58,60,61,64-66,69-71, 73-75,79,81,82,84,86,88,90,92,94,96,97,100]. There were 18 studies that recruited participants with mean ages ranging from 40 to 49 years [34,35,37,47,57,59,63,68,72,76,77,80,85,87,89, 91,99,101]. Eleven studies recruited participants with mean ages from 60 to 69 years [36,38,44,49,50,52,56,62, 67,83,93], and two studies recruited participants aged 70 and above [95,98]. Das et al [78] recruited participants with ages that ranged from 40 to 70 years and above. Recruitment ranged from 19 to 2305 participants, with reported attrition rates ranging from 0% to 67.4%.

### eHealth Interventions

The purpose of using eHealth as a health intervention varies from the self-management of chronic diseases to the prevention or delay of their onset. The majority of the included studies (39/70, 56%) used eHealth interventions for the self-management of chronic conditions [32-34,36,39-50,53,55,60-62,65,66,69-71,74-76,78,79,82,83,86,90,93-95,100]. With healthy older adults as participants, 14 studies utilized eHealth as an intervention for the prevention of chronic diseases [35,37,54,77,80,84,85,87,88,96-99,101]. Additionally, 17 studies implemented eHealth interventions to support the progression of diseases [38,51,52,56-59,63,64,67,68,72,73,81,89,91,92].

The interventions were most commonly performed for a duration of 3-6 months. Twenty-nine studies performed the intervention for 3 months or less [32,34,36,40,41,45,46,57,59,60,63-65, 70,74,76,78,80,82,86-88,91,93,96,97,101], 24 studies were performed for 4-6 months [33,37-39,42,44,47,48,51,53-56,58, 61,68,73,75,81,84,92,98-100], 13 studies involved interventions for a duration of 7-12 months [35,43,52,62,67,69, 70,84,85,89,90,94,95], and 3 studies performed the intervention for more than 12 months [66,70,79].

The studies included in this review reported the use of different technologies as their interventions, with the most common (22/70, 31%) being the use of mobile apps [36,40-42,44,47,53,57,63,64,66,68,75,77,84,89-91,96,97,99,101]. Twelve studies used a web-based app [34,43,62,65,67,69,70, 78,79,87,92,100], 9 studies used phone calls [49,50,56,59,60,74,81,83,93], and 12 studies used wearable technology in conjunction with other eHealth technologies [37,38,52,55,58,76,79,80,86,88,94,100]. Two studies used an automated program [51,98] and the remaining studies used email [61,70,79], text messages [33,39,45,46,48,82], or videoconferencing [54,73,76,78,95]. As shown in Multimedia Appendix 2, the eHealth focused on nutritional intervention only in 8 studies [32,36,43,60,71,72,74,77,92,99,101]. The remaining studies administered an intervention on nutrition and other health behaviors such as exercise, smoking cessation, medication adherence, and behavioral change techniques for a better lifestyle.

Multimedia Appendix 5 presents the types of features implemented by the eHealth apps for the studies included in the systematic review. Most of the studies implemented several types of features in their eHealth app to deliver the intervention to participants. The most common feature of the eHealth interventions was the distribution of educational content for health behaviors. The second most common feature was to allow users to record their health behaviors such as dietary behavior, clinical and anthropometric data, or physical activity levels, and included health reports generated to assess their adherence or performance during the intervention. Thirty-six studies allowed participants to set their own health goals, which they would aim to achieve by the end of the intervention. Motivational messages would be sent to participants to further encourage them to adopt or maintain health behaviors, or to continue to adhere to the intervention. Fifteen studies implemented a reminder system in the mHealth app to send reminders to users to input their health data, and some included a point-based system whereby participants would receive incentives when they successfully attained a health goal.

### Effectiveness of eHealth Interventions

A summary of the intervention findings that included anthropometric, clinical, behavioral, educational, and psychological outcomes is provided in Multimedia Appendix 6. In general, the studies found overall positive effects of the intervention in measured outcomes. The most commonly measured outcomes were weight (n=44), dietary behavior (n=30), BMI (n=27), HbA_1c_ levels (n=24), and physical activity levels (n=23). Among outcomes reported by five or more studies, adherence to health behaviors, Framingham CVD risk score, dietary behavior, weight, and BMI were those that showed the most common improvements in favor of the eHealth intervention group. Specifically, 5/7 (71%) of studies that reported adherence to health behaviors, 3/5 (60%) studies for Framingham CVD risk, 14/30 (47%) studies for dietary behavior, 20/44 (45%) studies for weight, and 11/27 studies (41%) for BMI showed improvements in favor of the intervention group. Multimedia Appendix 6 presents a summary of results for each included study at the latest measurement time point (ie, end of intervention or last follow-up), showing positive, negative, or no change to outcomes measured for all included studies in the intervention and control or comparison groups. Multimedia Appendix 7 presents a more comprehensive overview of the results for each study included in the systematic review. There are several cautionary notes about the results reported in some of the included studies. Bentley et al [86] did not perform inferential analyses of secondary outcomes (weight and HbA_1c_ level) due to a small sample size. Thus, positive results in favor of the eHealth intervention group should be interpreted with caution. Recio-Rodriguez et al [97] did not provide follow-up results on secondary outcomes (blood pressure, waist circumference, and BMI).

### Meta-analysis

Summary of the meta-analysis findings are reported in Table S1 in Multimedia Appendix 8, including heterogeneity, mean differences and 95% CIs, as well as the significance of the intervention effect. The forest plots for each variable reported in Table S1 are shown in Figures S1-S12 in Multimedia Appendix 8. The *P* values for heterogeneity showed significance for nearly all variables included in the meta-analysis. This indicates significant clinical heterogeneity [102] among the studies, possibly due to variability in the eHealth nutritional interventions administered. The forest plots also show the diverse effects of the eHealth nutritional interventions on the outcomes reported in the meta-analysis. Nonetheless, we considered it to still be worthwhile to summarize the results quantitatively with a random-effects model [102]. The effects of eHealth interventions on weight, BMI, waist circumference, low-density lipoprotein-cholesterol, systolic blood pressure, and HbA_1c_ level favored the intervention group (all *P*<.001) as compared to the control group, whereas the effects on fruit and vegetable consumption favored the control group (*P*=.01). In addition, the effects of the eHealth intervention on body fat, triglyceride level, Framingham CVD risk, and calorie intake favored neither the intervention nor the control group (all *P*>.05). Subgroup analyses based on the duration of the intervention period (ie, ≤3 months, 4-6 months, 7-12 months) on the outcomes of weight, waist circumference, and HbA_1c_ levels indicated high and significant heterogeneity for many variables (Figures S1, S3, and S7 in Multimedia Appendix 8); thus, the conclusions based on these results should be considered with caution.

### eHealth Usage

The majority of the studies included in the systematic review neither reported participants’ adherence to the eHealth app nor the participants’ perceptions and satisfaction toward the eHealth intervention. Only two studies reported higher adherence to the mHealth interventions among the intervention group when compared to control groups [33,82,97]. A few studies reported that overall, the eHealth apps were well-received by participants, as they found it to be useful [36], easy to use [32,33,36,37,55,68,86], and were satisfied with their respective eHealth apps [33,68]. Interestingly, two studies found that older participants were more likely to use the eHealth intervention app more frequently than younger participants [88,90]. However, Fukuoka et al [55] raised the issue of decreasing adherence to the mHealth intervention due to technical issues with the mobile app and pedometer. Similarly, Mundi et al [68] found a decrease in mobile app usage over the study period, even though participants reported that they were generally satisfied with the mobile app.

### Study Quality Assessed by the CONSORT 2010 Checklist

Multimedia Appendix 9 presents a detailed quality assessment of each study, with their raw and percentage scores indicated. The percentage scores for all included studies ranged between 44% and 85%. Overall, the quality for all included studies was judged to be “fair,” with a mean percentage score of 65.85%. Two studies were assessed to have low quality [40,63] with percentage scores below 50%. Ten studies were ranked as fair with percentage scores between 50% and 59% [34,38,41,49,58,64,74,84,94,95]. Twenty-five studies with percentage scores ranging from 60% to 69% were assessed as good quality [32,35,39,45,52-54,56,57,60,63,65,67,70,71,83, 85,86,89-93,96,98], and 18 studies were ranked as very good quality with a score of 70% and above [33,36,37,48, 50,51,55,59,61,62,66,69,72,73,75,87,88,97].

## Discussion

### Principal Results

The findings of the studies included in this systematic review and meta-analysis provided varying evidence for the effectiveness of eHealth interventions in improving health and other related outcomes for the prevention and management of chronic diseases related to poor nutrition. Results from the systematic review demonstrated that eHealth interventions were highly successful in significantly improving adherence to health behavior, Framingham CVD risk, dietary behavior, weight, and BMI.

The results of the meta-analysis revealed overall positive within-group improvements in favor of the intervention group for anthropometric (ie, weight, BMI, waist circumference) and clinical (ie, low-density lipoprotein-cholesterol, systolic blood pressure, HbA_1c_ level) outcomes. No within-group improvements were found in fasting blood glucose, body fat, triglyceride level, Framingham CVD risk, and calorie intake. Subgroup analyses based on the intervention duration were performed for outcomes of weight, waist circumference, and HbA_1c_ levels. Regardless of the intervention duration, there were significant improvements in weight. However, the highest improvement in weight was found for interventions administered for 4-6 months. Similarly, interventions that were administered for exactly 4-6 months improved waist circumference and had the largest improvement on HbA_1c_ levels compared with other intervention durations. In other words, interventions offered for less than 4 months and for more than 6 months showed no significant improvements in waist circumference, and improvements in weight and HbA_1c_ level were lower as compared to those achieved when interventions were delivered for 4-6 months. This could most likely be due to the effectiveness of eHealth interventions often only being measurable after a minimum intervention duration. Moreover, studies with longer intervention durations could show less effectiveness possibly due to unsustainable rates of adherence and drops in compliance of the study participants over time. Nevertheless, interpretation of these results requires caution owing to the significant heterogeneity among studies included in the meta-analysis. With regard to behavioral outcomes, improvements in fruit and vegetable consumption were more apparent in the control group. However, caution is also needed in interpreting this finding as the meta-analysis only included three studies that measured fruit and vegetable consumption. Indeed, it is also important to consider that not all studies in the systematic review were included in the meta-analysis, as some studies reported insufficient data, or used very diverse instruments or methods to measure their outcomes. Therefore, caution is also required in interpreting the results related to the effectiveness of eHealth on health and behavioral outcomes.

Furthermore, discrepancies in the findings of the meta-analysis (ie, improvements in anthropometric and clinical outcomes, but not behavioral outcomes) suggest that the improvements found in eHealth intervention groups could be due to the fact that many studies not only focused on nutrition but also on other lifestyle behaviors. Moreover, several studies [32,33,36,57] found improvements in dietary behavior or adherence to health behavior, but did not find any improvements in anthropometric outcomes or clinical outcomes (eg, weight, BMI, blood pressure, cholesterol). This contrast in findings begs the question as to whether improvements in health outcomes were brought about by improvements in dietary behavior. Therefore, future studies should further evaluate the relationship between improvements in behavioral and health outcomes, and whether such relationships will lead to longer-lasting effects. This would help validate the efficacy of the eHealth intervention, and its effectiveness in improving and maintaining health.

The implementation of eHealth technologies to deliver nutritional interventions provides great convenience and potential for health care systems, as participants are able to receive efficient, timely, and personalized health services. Previous research acknowledged the advantages of using eHealth in providing health services to older adults in a cost-effective and convenient manner [20,23]. Moreover, Kampmeijer et al [19] demonstrated that eHealth services are generally widely accepted among the older population. As these findings suggested that future work is needed to evaluate the effectiveness of eHealth on health outcomes, our current work bridges this gap in the literature by providing further evidence and considerations on the relevance of using eHealth for middle-aged and older populations.

### Future Considerations

Due to the exploratory nature of our review, we included a considerably broad range of eHealth interventions, target outcomes, as well as population groups. To address concerns of heterogeneity found in the meta-analysis, future meta-analyses should focus on specific types of eHealth interventions (eg, mobile apps), specific primary outcomes (eg, weight or BMI), or specific population groups (eg, patients with hypertension). With heterogeneity addressed in this way, results and conclusions drawn upon these studies would then be able to offer insight into the efficacy of eHealth interventions on specific outcomes. Subgroup analyses could also be performed to evaluate if different intervention durations have differential effects on target outcomes.

The vast majority of the included studies used a multicomponent intervention, whereby the eHealth technologies were most commonly used to provide nutritional and physical exercise intervention. In fact, we found only six studies that solely delivered nutritional interventions, and reported mixed results on the efficacy of eHealth to participants’ health outcomes [32,36,60,71,74,92]. The meta-analysis further showed that the intervention duration did not have a significant impact on health outcomes, suggesting that there could be other factors (eg, types of eHealth used or target population groups) that contributed to the effectiveness of such interventions. Having multicomponent interventions delivered through eHealth apps would understandably be more effective as compared to stand-alone nutritional interventions. Previous systematic reviews on mHealth app usage by adolescents and adults found similar favorable results for multicomponent interventions [13]. Moreover, for one to achieve a healthy and balanced lifestyle, it would be necessary to make positive changes to both diet and exercise simultaneously, rather than focusing on diet or exercise alone [103]. Thus, future developments of eHealth apps could consider having multicomponent intervention features to improve the health outcomes of users.

By and large, participants were reportedly satisfied with the eHealth technologies in helping them to achieve their health goals. However, a large number of studies did not systematically measure participants’ adherence, perception, and satisfaction toward the eHealth technologies. Future studies on the efficacy of eHealth interventions should measure these aspects, as it is important to have a better understanding of participants’ evaluation of the eHealth technologies from a qualitative and quantitative perspective. Otherwise, if the interventions provided are not widely accepted by participants, this might reduce their efficacy. Furthermore, if we consider the lack of a scientific theory and framework for app development [104], it would be imperative for future studies to consider participants’ engagement and evaluation of eHealth technologies, which might effectively improve future app development.

### Strengths and Limitations

One of the limitations of this systematic review was the search strategy employed to obtain relevant research articles. We searched for articles from five databases, which might have led us to miss relevant articles in the literature. However, we considered that the databases used for our search were sufficiently extensive in covering topics on eHealth and health services. We also manually searched articles that were not found in our selected databases. In addition, there are many ways to describe the search terms, which we might not have exhaustively included in our search strategy. However, we do consider the inclusion of MeSH terms, and general terms such as “telemedicine” and “nutrition” should suffice in comprising the main terms and concepts of interest. Nevertheless, the large number of articles identified, screened, and excluded does indicate that the search query could have been more specific. In addition, the use of Boolean operators (OR, AND) in our search query might have led to different interpretations by the various databases. Another limitation of our search strategy was the timeframe of searching for articles published in the last 5 years. We considered a 5-year timeframe as sufficient, as technologies used in eHealth tend to change and improve rapidly, and therefore become outdated in a short period. As such, studies using older technologies might already be outdated. We encountered important clinical heterogeneity in our meta-analysis; however, we still decided to report our findings in the appendices to illustrate that although these studies aimed to measure the same outcomes, there was high heterogeneity due to the diverse ways the interventions were administered (eg, mobile apps vs web-based tools). In addition, due to the different types of interventions administered across studies, the issue of clinical heterogeneity [102] makes it difficult to conclude consistent and convincing meta-analysis findings. Finally, we did not account for the quality of studies in our subgroup analyses. Variability in study quality may overestimate positive results, especially if studies with poorer quality are included in the meta-analysis. Nonetheless, the majority of our studies were ranked as being of good or very good quality; thus, we might expect less variability on the effect of study quality on subgroup analyses.

One strength of our study was that we had two independent reviewers performing the selection process and a third independent reviewer solving any disagreements in study inclusion. In addition, we followed the PRISMA-P 2015 guidelines for reporting a systematic review, and checked the quality of each included study using the CONSORT 2010 checklist in a standardized manner. We did consider additional ways to assess study quality, such as using the Cochrane risk of bias tool [105], which assesses each study outcome. However, we found that due to the exploratory nature and scope of our research aims, we were considering a rather large number of studies (70 in total), each having multiple primary and secondary outcomes. This made it challenging to assess the risk of bias for each outcome across all studies. Although the CONSORT checklist does not explicitly assess risk of bias, it does score and assess whether studies have adequately designed, analyzed, interpreted, and reported their results and methods, which we believe is an acceptable and valid way of assessing study quality and risk of methodological bias [13,27].

### Conclusion

We found varying evidence for the effectiveness of eHealth in providing nutrition-related interventions for middle-aged and older adults. Studies included in the systematic review generally reported positive support for the use of eHealth technologies in improving health and behavioral outcomes. Apps that delivered multicomponent interventions for improving nutrition and other health behaviors were more commonly used in health interventions as compared to stand-alone apps. The use of eHealth technologies has been increasing over the years, and has great potential in effectively delivering health services to a large group of people. Meta-analysis findings, although based on heterogeneous data and with quality limitations, generally demonstrated improvements in weight and BMI for eHealth users. To address heterogeneity in this field of research, future studies could look into developing a research framework or consensus in administering studies that involve eHealth interventions. Nevertheless, more research is needed to measure participants’ engagement with the technologies, and to provide structural and scientific frameworks for the development of future apps, which can provide a better understanding of their effectiveness and encourage app adherence among users.

## Appendix

Multimedia Appendix 1 Search strategies used for each database search.

Multimedia Appendix 2 Characteristics of included studies.

Multimedia Appendix 3 PRISMA-P 2015 Checklist.

Multimedia Appendix 4 Funnel plots.

Multimedia Appendix 5 Types of features implemented by eHealth apps.

Multimedia Appendix 6 Summary of findings.

Multimedia Appendix 7 Comprehensive overview of studies included in the systematic review.

Multimedia Appendix 8 Summary of the meta-analysis results and forests plots.

Multimedia Appendix 9 Study quality assessed by CONSORT 2010 Checklist.

## Abbreviations

CVD: cardiovascular disease
HbA_1c_: glycosylated hemoglobin (A_1c_)
MeSH: Medical Subject Headings
mHealth: mobile health
PRISMA-P 2015: Preferred Reporting Items for Systematic Reviews and Meta-Analyses for Protocols 2015
RCT: randomized controlled trial
