# Supplementary material for: Effectiveness of eHealth Nutritional Interventions for Middle-Aged and Older Adults: Systematic Review and Meta-analysis
Source: J Med Internet Res. 2021 May 17;23(5):e15649. doi: 10.2196/15649 (PMC8167617; doi:10.2196/15649)
Supplement: Multimedia Appendix 1 [file jmir_v23i5e15649_app1.docx]

Multimedia Appendix 1. Search strategies used for each database search

Pubmed (Medline)

| Searched for all fields: | telemedicine OR mobile health OR health OR mobile OR mhealth OR telehealth OR ehealth  nutrition OR Diet OR Food OR eating OR food intake OR ingestion OR diet habit  middle aged OR aged OR aged, 80 and over OR elderly |
| --- | --- |
| Filter selection: | Full text, 2014-2019, Humans, English, Medline, Middle-aged + Aged: 45 + years , Middle Aged: 45-64 years, Aged: 65+ years, 80 and over: 80+ years |
| Date of Search | 20 May 2020 |
| Hits: | 629 |

CINAHL Complete

| Searched for all fields: | telemedicine OR mobile health OR health OR mobile OR mhealth OR telehealth OR ehealth  nutrition OR Diet OR Food OR eating OR food intake OR ingestion OR diet habit  middle aged OR aged OR aged, 80 and over OR elderly |
| --- | --- |
| Filter selection: | Full text, 2014-2019, English, Research article, Human, Middle aged: 45-64 years, Aged: 65+ years, Aged: 80 and over |
| Date of Search | 20 May 2020 |
| Hits: | 492 |

Cochrane

| Searched for all fields: | telemedicine OR mobile health OR health OR mobile OR mhealth OR telehealth OR ehealth  nutrition OR Diet OR Food OR eating OR food intake OR ingestion OR diet habit  middle aged OR aged OR aged, 80 and over OR elderly |
| --- | --- |
| Filter selection: | RCT and reviews, CT.gov, Embase, ICTRP  2014-2019 |
| Date of Search | 20 May 2020 |
| Hits: | 9217 |

Web of Science

| Searched for all fields: | telemedicine OR mobile health OR health OR mobile OR mhealth OR telehealth OR ehealth  nutrition OR Diet OR Food OR eating OR food intake OR ingestion OR diet habit  middle aged OR aged OR aged, 80 and over OR elderly |
| --- | --- |
| Filter selection: | RCT and reviews  2014-2019 |
| Date of Search | 20 May 2020 |
| Hits: | 758 |

Global Health

| Searched for all fields: | telemedicine OR mobile health OR health OR mobile OR mhealth OR telehealth OR ehealth  nutrition OR Diet OR Food OR eating OR food intake OR ingestion OR diet habit  middle aged OR aged OR aged, 80 and over OR elderly |
| --- | --- |
| Filter selection: | All fields, Filter, articles and book, 2014 to 2019, English |
| Date of Search | 20 May 2020 |
| Hits: | 126 |
