# Supplementary material for: Effectiveness of eHealth Nutritional Interventions for Middle-Aged and Older Adults: Systematic Review and Meta-analysis
Source: J Med Internet Res. 2021 May 17;23(5):e15649. doi: 10.2196/15649 (PMC8167617; doi:10.2196/15649)
Supplement: Multimedia Appendix 2 [file jmir_v23i5e15649_app2.docx]

Multimedia Appendix 2. Characteristics of included studies.

| **Study** | **Population** | **Intervention** | **Control**  **and Timing** | **Outcome** |
| --- | --- | --- | --- | --- |
| Aalbers et al (2016), Netherlands [72] | Healthy adults (N=2,305) | Mobile app games to assess cognitive function and set lifestyle goals. Monitoring goals and feedback. Weekly blogs on heath and healthy recipes. | 2-group pre-post study;  Baseline and 12 months | Lifestyle-specific goal-setting success rate, Weight, BMI |
| Ahn et al (2016), South Korea [32] | Patients with DM (N=26) | Web- and mobile-based app to record daily dietary intake. Dietary patterns and results of nutrient intake. Reminder to input dietary data. Weekly nutrition classes. | 2-group pre-post study;  Baseline and 1 month | Weight, BMI, Dietary attitude and behavior, Nutritional knowledge |
| Akhu-Zaheya & Shiyab (2016), Jordan [42] | Patients with CVD (N=160) | Mobile phone reminder text messages for taking medication, smoking cessation, and importance of healthy diet. | 3-group RCT;  Baseline and 3 months | MEDAS, MMAS, Readiness to Quit Smoking Ladder, Number of cigarettes smoked |
| Alencar et al (2017), United States [89] | Obese participants (N=25) | Wireless devices (body composition scale, activity tracker, accelerometer, BP cuff). Mobile health coaching. Video conferencing. Weight loss program. Feedback from dietitian. Nutrition and fitness education. | 2-group RCT;  Baseline and 12 weeks | Weight, BMI, Body fat, Weekly step change, BP |
| Ambeba et al (2015), United States [90] | Obese adults (N=210) | PDAs to record dietary intake and receive feedback. Behavioural weight loss treatment. Group sessions. Dietary and exercise goals. Self-monitoring. | 2-group RCT;  Baseline, 6, 12, 18 and 24 months | Dietary intake |
| Axley et al (2017), United States [43] | Patients with chronic liver diseases (N=30) | Mobile phone motivational messages to maintain health-related behaviours. Educational content on nutrition, exercise, and stress management. | 2-group RCT (pilot);  Baseline and 6 months | Weight, BMI*,* Liver enzyme and lipid profile |
| Balk-Møller et al (2017), Denmark [73] | Healthy older adults (N=566) | Web- and mobile-based app to set health goals, record dietary intake, exercise levels. Feedback, messages, points system, content and weekly assignments. Group challenges. | 2-group RCT;  Baseline, 4 and 9.5 months | Weight*,* Body fat percentage*,* WC*,* BP, Cholesterol |
| Barnason et al (2019), United States [44] | Patients with CVD (N=43) | Telehealth sessions via telephone to educate on caloric intake, and portion control guidelines. | 2-group RCT;  Baseline, 4 and 6 months | Weight, BMI, PA levels, Perceived self-efficacy, Weight management behaviours |
| Benson et al (2019), United States [45] | Patients with T2DM (N=118) | Telephone coaching to motivate, educate, set goals, and identify barriers in achieving a healthy lifestyle. Portion control, calorie reduction, increase in fruit/vegetable, and healthy fats. | 2-group RCT;  Baseline and 12 months | HbA1c, BP, Tobacco use, Statin and aspirin use, Adherence to Mediterranean diet, PA level, Dietary intake, BMI, LDL-C, Medication adherence |
| Bentley et al (2016), United Kingdom [74] | Patients with T2DM (N=27) | Wearable device to track PA levels and nutritional intake, food type, portion sizes, or number of calories. Event diaries. Training on weight loss and self-management of hbA1c level. Training on AiperMotion 500. | 3-group RCT;  Baseline, 6, 12, and 16 weeks | Acceptance, adherence, and satisfaction towards wearable device, Weight, HbA1c |
| Block et al (2015), United States [46] | Patients with prediabetes (N=339) | Automated behaviour change program. Set goals to improve dietary habits, increase dietary fibre intake, decrease saturated and trans fats, added sugars, and refined carbs. | 2-group RCT;  Baseline, 3 and 6 months | FBG*,* HbA1c*,* Weight*,* BMI*,* WC*,* TG/HDL ratio*,* Framingham diabetes risk score |
| Castro Sweet et al (2018), United States [47] | Adults at risk of diabetes (N=501) | Website and mobile app to set health goals, track progress (weight, PA and dietary intake), interact with others, receive feedback from health coach. Pedometer to track PA levels. | Single-arm pre-post study;  Baseline, 4, 6, and 12 months | Weight, HbA1c, Total cholesterol, WHO-5, PHQ-4, SDSCA |
| Choi et al (2019), United States [48] | Patients with CVD (N=100) | Mobile app to take pictures and log dietary intake, and load educational content. Set goals and receive feedback. | 2-group pilot RCT;  Baseline, 3 and 6 months | Weight, BMI, MDS, BP*,* Cholesterol*,* Triglycerides, HbA1c, CRP |
| Clark et al (2019), United States [49] | Healthy adults (N=150) | Video-conferencing educational lessons on PA, diet and portion control. Consultation with health coach. Self-monitor PA and dietary intake. | 3-group RCT;  Baseline, 6 and 12 months | Proportion of participants who reduced ≥2 kg, Weight, Attendance or usage of intervention |
| Das et al (2017), United States [88] | Adults (N=644) | Website message board, log weight on website. Weekly video conference group meetings. Nutrition and weight management education, support and discussion. | Pre-post study;  Baseline and 11 weeks | Weight loss |
| Dennison et al (2014), United Kingdom [75] | Healthy adults (N=786) | Web-based program for self-monitoring. Select diet plan, set health goals, receive reminders and cognitive and behavioural strategies, coaching calls. | 3-group RCT;  Baseline and 2 months | Weight loss, Usage of POWeR programme |
| Duan et al (2018), China (Hong Kong) [34] | Patients with coronary heart disease (N=114) | Web-based program to set health goals and self-monitor PA levels and FVC. Develop action plan to achieve health goals. Receive feedback. | 2-group RCT;  Baseline and 2 months | PA levels, fruit/vegetable consumption, BMI |
| Duncan et al (2014), Australia [35] | Healthy male participants  (N=301) | Mobile app to self-monitor PA and dietary patterns, set health goals. Challenges, interact with others, educational materials, and receive feedback. | 2-group RCT;  Baseline, 3 and 9 months | Dietary behavior, PA level, Health literacy, Usage of IT-based intervention |
| Elbert et al (2016), Netherlands [91] | Healthy adults (N=342) | Mobile app to receive text or audio-based health information and feedback to stimulate fruit/vegetable intake. | 3-group RCT;  Baseline and 6 months | Dietary intake (fruit consumption) |
| Eyles et al (2017), New Zealand [36] | Patient with CVD (N=66) | Mobile app to scan barcode of packaged food and receive nutritional information. Weekly text messages to remind and encourage usage of app. | 2-group RCT;  Baseline and 1.5 months | Salt content of food purchases, Saturated fat content of food purchases, Energy content of food purchases, Systolic BP, Urinary sodium |
| Fernandes et al (2016), Brazil [71] | Diabetes patients (N=110) | Telephone calls to encourage healthy behaviours, build rapport, provide support, guidance on PA and diet plan. | 2-group RCT;  Baseline, 3 and 6 months | Diabetes self-care scores |
| Fukuoka et al (2015), United States [50] | Middle-aged adults at risk of diabetes (N=61) | Mobile app to self-monitor health data, PA levels, and caloric intake. Set health goals, provide dietary recommendations, reminder to record data. Pedometer to record PA levels. | 2-group pilot RCT;  Baseline, 3 and 5 months | Weight*,* BMI*,* WC*,* BP*,* Lipid profile*,* Glucose, PA levels, Caloric and fat intake (Block Food Frequency Questionnaire)*,* 7-day PA recall*,* Self-efficacy for PA*,* Social support and exercise survey*,* Depressive symptoms (CES-D) |
| Gilson et al (2017), Australia [37] | Healthy middle-aged adults (truck drivers) (N=19) | Mobile app and wearables to track step count. Incentive point-based system. Set health goals (step counts, healthier food choices). Record daily dietary choices, receive monthly feedback. | 2-group pre-post study;  Baseline, 5 and 7 months | Dietary intake (self-reported), PA level |
| Gomez-Marcos et al (2018), Spain [76] | Healthy older adults (N=833) | mHealth app to promote healthy diet and increase PA. Record daily food intake. Pedometer in smartphone to record step counts and PA. Analysis, counselling and plan to improve diet and increase PA. | 2-group RCT;  Baseline, 3 and 12 months | BMI, WC, CUN-BAE |
| Haas et al (2019), Switzerland [77] | Patients with obesity (N=43) | Mobile app to communicate with dietician. Self-monitor diet, PA levels. Nutritional content, set health goals, feedback and support. | Pre-post pilot study;  Baseline, 3 and 12 months | Weight, HbA1c, FBG, Triglycerides, Insulin level, HDL-cholesterol, BP, BMI, WC, Body fat, PA level, Dietary assessment, QoL |
| Hageman (2017), United States [92] | Women from underserved rural communities  (N=301) | Website with weekly behavior change lifestyle plan, eating and activity recommendations, peer-led discussion, blog, counselling email. Pedometer to record daily steps and PA. | 3-group RCT;  Baseline, 6, 18, and 30 months | Weight, WC, Kcal intake daily, Weekly minutes moderate or greater intensity activity, BP, Cholesterol, Triglycerides, FBG, Calorie intake, PA level |
| Haggerty et al (2017), United States [51] | Endometrial cancer survivors with obesity (N=41) | Telemedicine counselling sessions via telephone. Weight loss management. Daily text messages about weight loss management. | 3-group RCT;  Baseline and 6 months | Weight, WC, PA level, Physical Health SF-12 |
| Hales et al (2016), United States [52] | Overweight middle-aged adults (N=51) | Mobile app to track dietary intake and PA levels. Health information, reminders to record weight, notifications, set goals, and earn points for achieving goals. | 2-group RCT;  Baseline and 3 months | Weight, BMI, caloric intake and expenditure, social support for health behaviours (diet, exercise), self-efficacy for weight-loss behaviours, expectations of treatment |
| Hansel et al (2017), France [93] | Participants with abdominal obesity and T2DM with HbA1c >5.6% and <8.5%  (N=120) | Web-based nutritional support tool, automated digital scale, pedometer. Diet and PA self-monitoring, nutritional  assessment, balanced diet menu generator, PA education and prescription program. | 2-group RCT;  Baseline and 4 months | Weight, BMI, WC, BP, VO2, HbA1c, DQI-I score, IPAQ, FBG, Cholesterol, LDL-C, HDL-C, Triglycerides, Serum Glutamic Pyruvic, Transaminase, Serum Glutamic Oxaloacetic, Transaminase, Gammaglutamyl-transferase, hs-CRP, Uric acid, Creatinine |
| Hartman et al (2016), United States [53] | Women at-risk of breast cancer (N=54) | Mobile app to record weight and food intake. Fitbit to measure PA levels. Calorie targets, nutrition and PA reports. Telephone health coaching to support weight loss. | 2-group RCT;  Baseline, and 6 months | Weight, PA level |
| Holmen et al (2014), Norway [78] | Patients with T2DM (N=151) | mHealth app to self-manage diabetes. Record daily dietary and PA levels. Diabetes diary app to record and track blood glucose levels, food habits, PA levels, BP and health goals. Health reports, motivational feedback, and telephone health counselling. | 3-parallel group RCT;  Baseline, 4 months, and 12 months | Weight, heiQ score, Dietary habits, PA levels, QoL measured by Short-Form 36v2 Health Survey, Depressive symptoms measured by CES-D, HbA1c |
| Huber et al (2015), United States [54] | Patients with obesity (N=90) | Telecoaching on portion control plate and motivational interviewing to improve patients’ lifestyle habits. | 2-group RCT;  Baseline, 6, 12, 18, and 24 weeks | Weight, BMI, WC, Waist-to-hip ratio, Eating behaviours, PA level, WEL |
| Järvelä-Reijonen et al (2018), Finland [79] | Adults with psychological distress and obesity (N=219) | Mobile app with modules on acceptance and commitment therapy (ACT) in changing and improving health behaviours such as mindfulness, and healthy dietary habits. | 3-parallel group RCT;  Baseline, 10, and 36 weeks | IES score, TFEQ score, HTAS score, Eating competence, REBS score, Food and nutrient intake (IDQ score), Alcohol consumption, 48h dietary recall, PSS |
| Kanera et al (2017), Netherlands [80] | Cancer survivors (N=462) | Web-based intervention to set dietary goals, increase healthy eating behaviours (consuming more dietary fibre, whole grains, and fish), and receive advice on health modules (diet and nutrition). | 2-group RCT;  Baseline, 6, and 12 months | Daily food intake (8-item Dutch Standard Questionnaire on Food Consumption), frequency and serving sizes of vegetable consumption |
| Kempf et al (2017), Germany [81] | Patients with T2DM (N=202) | Telemedical coaching to motivate. Formula diet regimen. Self-monitor hbA1c levels, record weight and step count. | 2-group RCT;  Baseline, 12, 26, and 52 weeks | HbA1c, FBG, Weight, BP, Cholesterol, Triglycerides, CVD risk, QoL, Diet behavior, Antidiabetes medication |
| Khanna et al (2014), United States [55] | Patients with T2DM (N=75) | Telephone calls to educate on importance of lowering high-glycemic index foods. Self-assess and receive feedback on food intake by responding to telephone key pad. | 2-group RCT;  Baseline, and 3 months | HbA1c, BMI, WC, Cholesterol, BP, Triglycerides |
| Koot et al (2019), Singapore [87] | Patients with T2DM, and HbA1c ≥7.5%  (N=100) | Mobile app to log and monitor blood glucose levels, weight, meals. Pedometer to track PA. Educational online lessons. Health coach feedback on progress and opportunities for improvement. | Single-arm feasibility study;  Baseline, and 6months | HbA1c, weight, dietary habits, PA level |
| Lim et al (2016), South Korea [38] | Patients with HbA1c level <7% without hypoglycaemia (N=100) | Public switched telephone network connected to glucometer to measure blood glucose level. Wearables to record PA levels. Record daily dietary intake on website. | 2-group RCT;  Baseline, 3, and 6 months | HbA1c, BMI, Fat mass, BP, Cholesterol, Triglyceride, Clinical measures |
| Liu et al (2018), Canada [56] | Patients with hypertension  (N=128) | E-counseling emails to support health goals for BP management and to encourage healthy lifestyle behaviours (step counts, increased fibre intake, and exercise). | 3-parallel group RCT;  Baseline, and 4 months | Systolic BP, Cholesterol, Cardiovascular risk (10-year Framingham risk score), daily steps, dietary habits. |
| Lorig et al (2016), United States [57] | Participants with T2DM  (N=857) | Web-based app to educate on creating action plans, provide feedback, discussion with others, track PA level, food and medication intake. Community workshops on diabetes self-management. | 2-group pragmatic trial;  Baseline, 6, and 12 months | HbA1c, Health indicators (e.g., eye, foot, cholesterol, kidney exam), Aerobic exercise, General health, Psychological outcomes |
| Luley et al (2014), Germany [82] | Patients with metabolic syndrome  (N=184) | Accelerometer to measure PA level, and record dietary intake. Feedback and motivational messages via telephone calls from health coach. | 3-group RCT;  Baseline, 4, 8, and 12 months | Weight, BMI, WC, BP, Triglycerides, Cholesterol, Apolipoprotein-B, Uric acid, ALT, AST, CRP high sensitivity, HbA1c, Insulin, HOMA |
| Martin et al (2015), United States [94] | Healthy adults  (N=40) | Mobile app to educate on nutrition, record weight and receive feedback from counsellor. Health information via text messages, emails or wearables. | 2-group pilot RCT;  Baseline, 4, 8, and 12 weeks | Weight, WC, BP |
| Mason et al (2018), United States [58] | Women who were overweight  (N = 104) | Mobile app to track health goals, receive educational content (behavioural change and mindfulness to food cravings). Feedback on mindful eating. Reminders to check hunger and emotional state and discourage mindless eating. | Feasibility, pre-post study;  Baseline, and 1 month post-intervention | Weight, FCQ-TR score, RED score, PEMS score, Intervention engagement |
| McCarroll et al (2015), United States [59] | Overweight or obese endometrial and breast cancer survivors  (N = 50) | Mobile app to record food intake and PA levels. Monitor carb intake, limit daily cars intake and increase daily fibre intake. Motivational push-notifications to encourage daily recording of meals and PA levels. | Feasibility, pre-post study;  Baseline, and 1 month | Weight, BMI, WC, FACT-G, physical, social/family, emotional, and functional well-being, WEL, Nutrient quality daily food intake log |
| McKenzie et al (2017), United States [60] | Adults with T2DM (N=262) | E-health web-based app to provide biometric feedback, peer support, and educational content on management of diabetes in relation to diet, nutrition, and behavioural change techniques. | 2-arm pre-post study;  Baseline, and 10 weeks | HbA1c, FBG, BMI, weight, BP, cholesterol, triglycerides, Serum creatinine, ALT, AST, Alkaline phosphatase, CRP, white blood cell, change in medication prescription |
| Michaelides et al (2018), United States [61] | Participants with T2DM  (N=59) | Mobile app to interact with health coach, set daily health challenges, self-monitor and log PA and diet intake, receive feedback on food choices. Educational content and motivational messages. | Pre-post pilot study;  Baseline, 24, and 65 weeks | Weight, BMI, App usage |
| Miller et al (2017), USA [95] | Adults with mixed dyslipidaemia  (N=722) | Telephone-based health coaching by dietitians to promote healthy lifestyle, healthy diet and regular exercise. Dietary recall. Set health goals for weight loss. | 2-group pre-post study;  Baseline, and 6 months | Weight, BMI, cholesterol |
| Moin et al (2018), United States [62] | Participants who had obesity  (N=442) | Web-based app to educate on nutrition and exercise, interact with health coach, group chat, set weight goals. Self-monitor weight with wireless scale. | Pre-post, non-randomized comparative study;  Baseline, 6, and 12 months | Weight, App usage |
| Mundi et al (2015), United States [63] | *N* = 30  Patients undergoing bariatric surgery | Mobile app with educational videos (healthy eating, diet, healthy food choices, servings and portions, food labels) and PA (exercise, fitness plan). Supportive messages to encourage health behaviours. | Pre-post feasibility trial study;  Baseline and 15 weeks | Weight, BMI, Bariatric surgery knowledge questionnaire (self-developed), International PA Short Form |
| Nepper et al (2019), USA [96] | Patients with T2DM  (N=79) | Educational text messages on diabetes self-care activities, CVD risk awareness, home food availabilities related to food choices. | 2-group pre-post study;  Baseline, and 12 weeks | Diabetes self-care activities, CVD risk awareness, PA, dietary intake, Home Food Self-Inventory, satisfaction regarding feasibility and usefulness of intervention |
| Nolan et al (2018), Canada [64] | Patients with hypertension (N=264) | E-counselling to provide motivational and cognitive-behavioural skills to promote healthy lifestyle, nutrition and medication adherence. | 2-arm parallel RCT;  Baseline, 4, and 12 months | BP, pulse, cholesterol, Framingham CVD risk |
| Orlandoni et al (2016), Italy [83] | Patients treated with HEN (N=188) | During monthly home visits, video consultation via tablet with physician to examine patient and change medication or nutrition regimen. | 2-group RCT;  Baseline, monthly for 6.7 months | Incidence rate of complications, outpatient hospital visits, and hospitalisations |
| Peimani et al (2015), Iran [97] | Patients with T2DM (N=150) | Educational text messages on nutrition, exercise, medication adherence, blood glucose monitoring. SMS messages on barriers to diabetes self-management. | 3-group RCT;  Baseline, and 12 weeks | BMI, FBG, HbA1C, Lipid profile, SCI, DSCB, DMSES |
| Pfaeffli Dale et al (2015), New Zealand [33] | Patients with coronary heart disease (N=123) | mHealth supporting website and text messages to encourage healthy lifestyle changes (fruits/vegetables intake, smoking cessation, regular exercise). Pedometer to track PA. Cardiac rehabilitation. | 2-group parallel RCT;  Baseline, 3 and 6 months | Smoking habit, fruit/vegetable intake, alcohol intake, and PA. Adherence (EPIC-Norfolk Prospective Population Study) |
| Ramadas et al (2018), Malaysia [98] | Patients with T2DM (N=132) | Web-based app encouraging healthy eating behaviours (reduced sugar intake, improved eating out habits, and increased fruit/vegetable consumption). Reminder to log in if inactive with lesson plans. | 2-group RCT;  Baseline, 6, and 12 months | DKAB scores, DSOC, FBG, HbA1c |
| Recio-Rodriguez et al (2016), Spain [85] | Healthy older adults (N=833) | Mobile app to log food intake and exercise levels. Counseling on PA and Mediterranean diet. Dietary recommendation. Daily summary reports of food intake and PA performed, and plan to improve diet and increase PA. | 2-arm parallel RCT;  Baseline, and 3 months | MEDAS, PA level measured by PAR questionnaire and accelerometer, BP, WC, BMI |
| Recio-Rodriguez et al (2018), Spain [84] | Healthy older adults (N=833) | (Same as Recio-Rodriguez et al (2016), Spain). | 2-arm parallel RCT;  Baseline, 3 and 12 months | Nutritional composition of food intake measured by Food Frequency Questionnaire |
| Shahid et al (2014), Pakistan [99] | Rural diabetic patients with T2DM (N=440) | Mobile phone to record blood glucose readings and receive regular feedback. | 2-group RCT;  Baseline, and 4 months | BP, Hypertension, BMI, HbA1c, LDL-C, number of participants following diet plan and/or physically active |
| Santo et al (2018), Australia [39] | Patients with coronary heart diseases (N=710) | Mobile text messages advising and encouraging healthy lifestyle change (cardiovascular health, smoking cessation, PA, increased fibre and fish intake, decreased fats and high-salt foods, healthy cooking methods. | 2-arm parallel RCT;  Baseline, and 6 months | Dietary intake (10-item survey based on WHO STEPS instrument) |
| Saslow et al (2017), United States [65] | Participants with T2DM (N=25) | Receive emails with links to online resources encouraging very low-carbs ketogenic diet and positive lifestyle changes. | 2-group RCT;  Baseline, 16, and 32 weeks | HbA1c*,* weight*,* triglycerides*,* cholesterol, Diabetes Distress Scale*,* CESD scale*,* mDES scale*,* Self-report physical health*,* diet intake |
| Saslow et al (2018), United States [66] | Adults with T2DM (N=1000) | Web-based platform to educate on nutrition and diet, self-monitor and track health data, set health goals, reduce carbs intake, and obtain feedback on progress. | Pre-post study;  Baseline, and 12 months | HbA1c, BMI*,* medication prescription |
| Sepah et al (2017), United States [67] | Adults with prediabetes (N=220) | Web-based platform and mobile devices to record and monitor health data, access to health coach, peer support, and educational content (nutrition and exercise). | Pre-post study;  Baseline, 16 weeks, 1, 2, and 3 years | HbA1c, weight, program attendance |
| Sun et al (2019), China, [100] | Patients with T2DM (N=91) | mHealth management app to upload glucometer data via Bluetooth. Medical advice, reminders via personal messaging app. Record PA data via text messaging. | 2-group RCT;  Baseline, 3, and 6 months | HbA1c, PBG, Cholesterol, BMI, BP, FBG, Triglyceride, Satisfaction |
| Vadheim et al (2017), United States [68] | Overweight adults (N=894) | Telehealth videoconferencing to improve lifestyle behaviours by encouraging self-monitoring diet, weight, increasing PA levels and reducing fat intake. | 2-group pre-post study;  Baseline, 0-6, 7-13, and 14-16 weeks | Programme attendance*,* PA level*,* Weight*,* BMI |
| van Doorn-van Atten et al (2018), Netherlands [86] | Healthy older adults (N=204) | Telemonitoring with set-up box connected to television with Internet connection to record weight, step count, and BP. Receive nutrition education, nutritional information and advice, follow-up session with nurse. | 2-group pre-post study;  Baseline, 4.5, and 6 months | Mini Nutritional Assessment, weight, Dutch Health Diet-FFQ, Simplified Nutritional Appetite Questionnaire, Katz-15 Short Physical Performance Battery, QoL (MOS 36). |
| Ventura Marra et al (2019), United States [69] | Men with chronic diseases related to obesity (N=59) | Telephone health coaching, videoconferencing to discuss goal setting, weight change, and overcoming barriers to poor dietary habits. Self-monitor weight, dietary behaviours (fruit/vegetable intake, dietary health goals). | 2-group pilot RCT;  Baseline, 6, and 12 weeks | Weight, Body fat, Waist circumference, Energy intake, Dietary quality |
| Waki et al (2014), Japan [40] | Patients with T2DM (N=54) | Mobile app to record dietary intake, record weight, step count, BP, receive evaluation and feedback from health professional. | 2-group RCT;  Baseline, and 3 months | HbA1c, FBG, BMI, Cholesterol, BP, Diabetes self-management (diet and exercise) |
| Wayne et al (2015), Canada [70] | Patients with T2DM (N=131) | Mobile app to record daily food intake, PA levels. Set health goals, track progress, contact health coach. | 2-group RCT;  Baseline, 3, and 6 months | HbA1c, Weight, BMI, WC, Satisfaction with life, HADS, PANAS, SF-12 |
| Whitelock et al (2019), UK [101] | Overweight adults (N=107) | Mobile app to record and take pictures of meals. Reminder to review past recordings before mealtimes. Dietary information. | 2-group RCT;  Baseline, 4, and 8 weeks | Weight, Energy intake, Body fat |
| Zhou et al (2016), China [41] | Patients with DM (Type 1 and 2) (N=100) | Mobile app to set goals and medication regimens. Record self-care data (dietary intake, blood glucose values). Communicate with clinicians and receive feedback. Information on diet and exercise. | 2-group RCT;  Baseline, and 3 months | HbA1c, FBG, LDL-C*,* Weight*,* BP*,* Hypoglycemic events*,* Diabetes knowledge*,* Self-care behaviours |
