# Supplementary material for: Effectiveness of eHealth Nutritional Interventions for Middle-Aged and Older Adults: Systematic Review and Meta-analysis
Source: J Med Internet Res. 2021 May 17;23(5):e15649. doi: 10.2196/15649 (PMC8167617; doi:10.2196/15649)

Multimedia appendix 4. Funnel plots.

Figure S1. Funnel plots for weight.


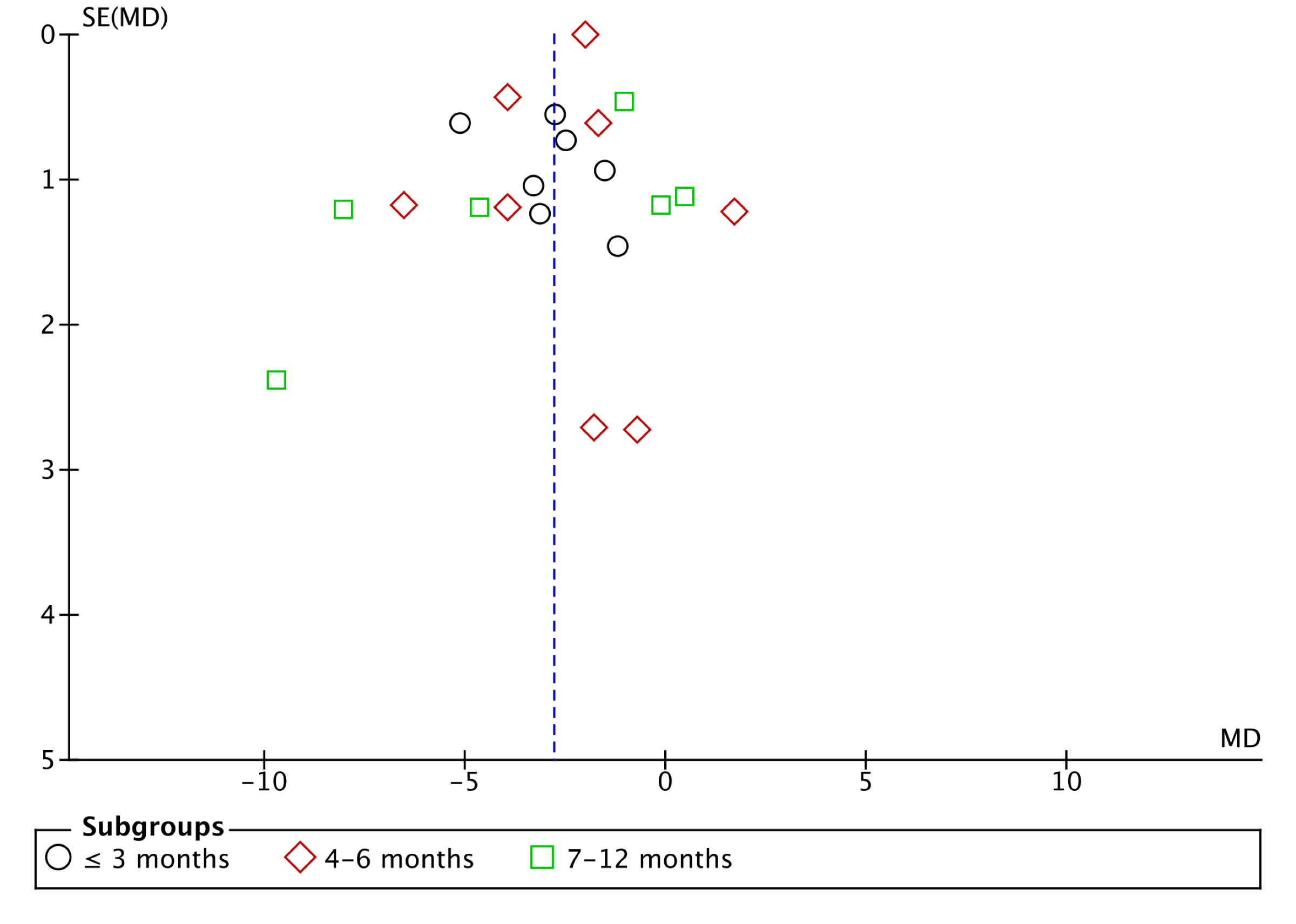


Figure S2. Funnel plots for BMI.


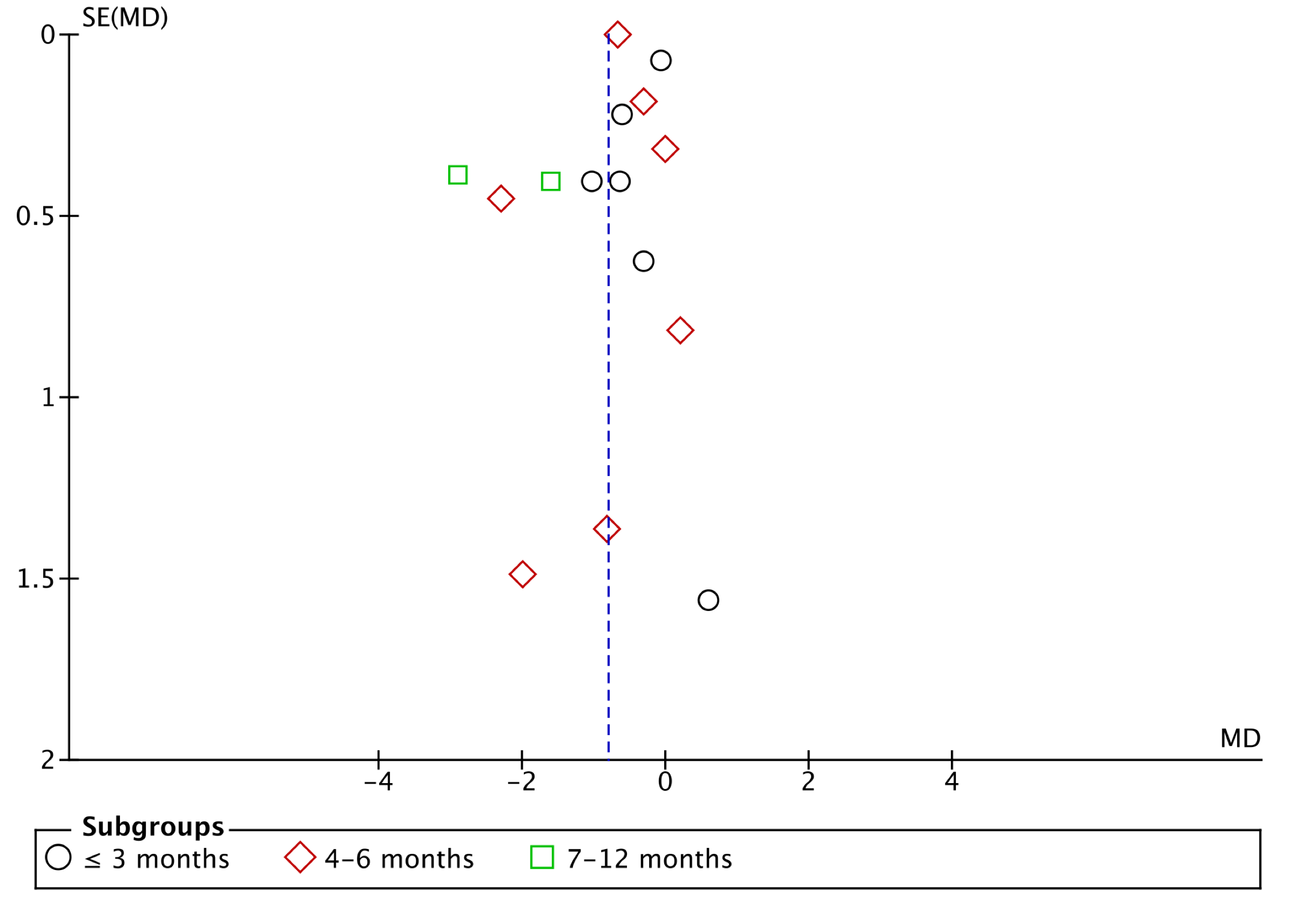


Figure S3. Funnel plots for waist circumference.


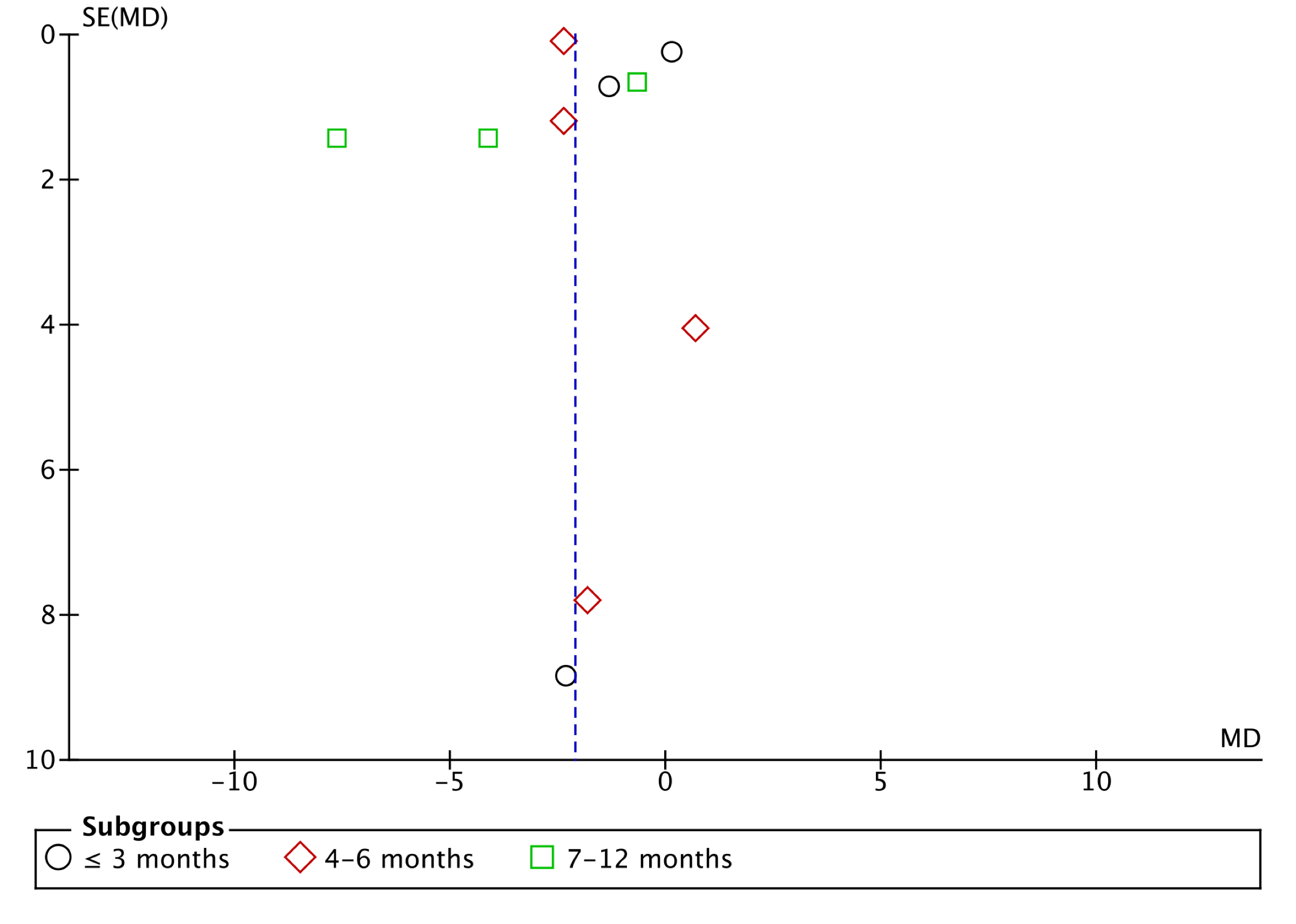


Figure S4. Funnel plots for LDL-cholesterol.


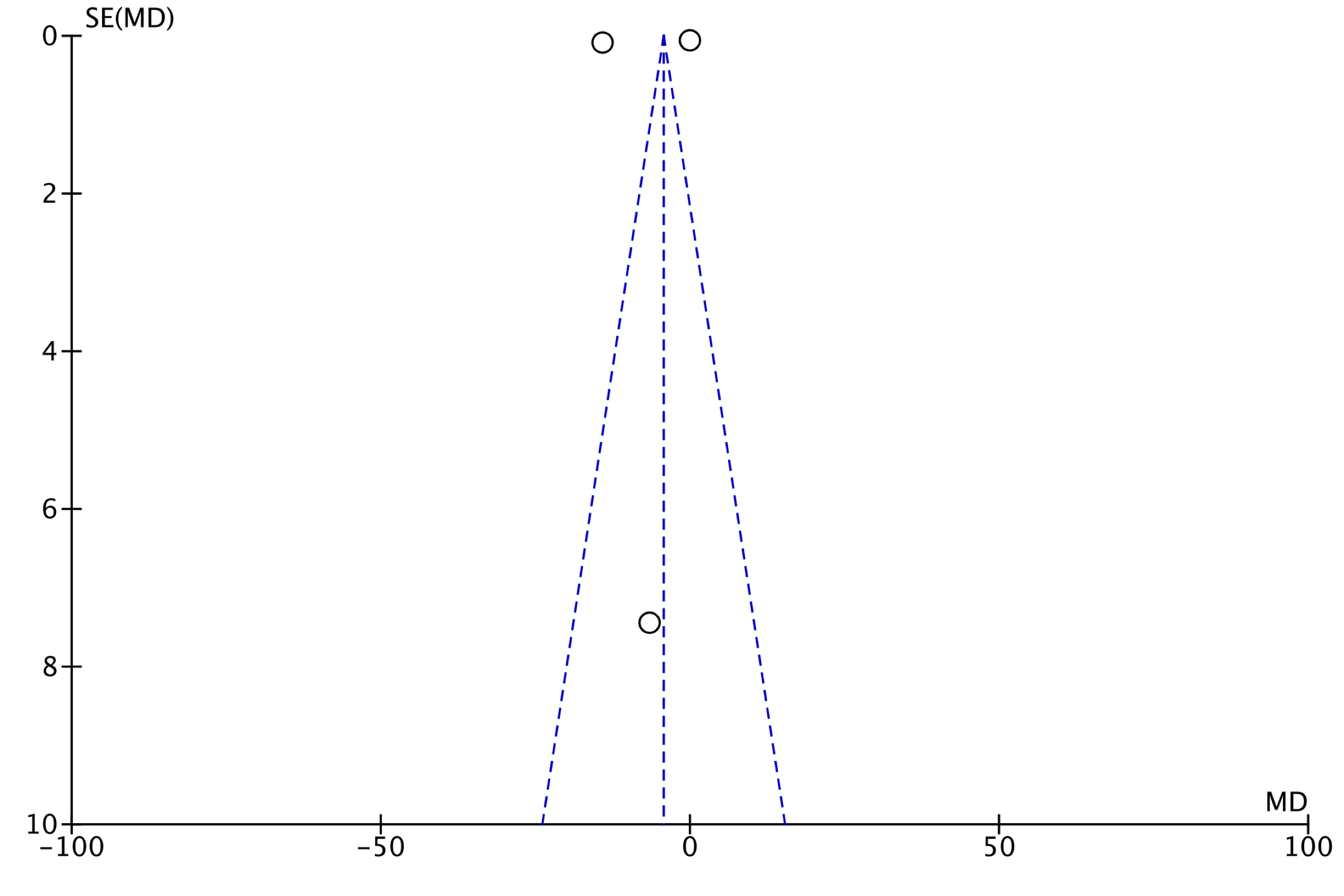


Figure S5. Funnel plots for systolic blood pressure.


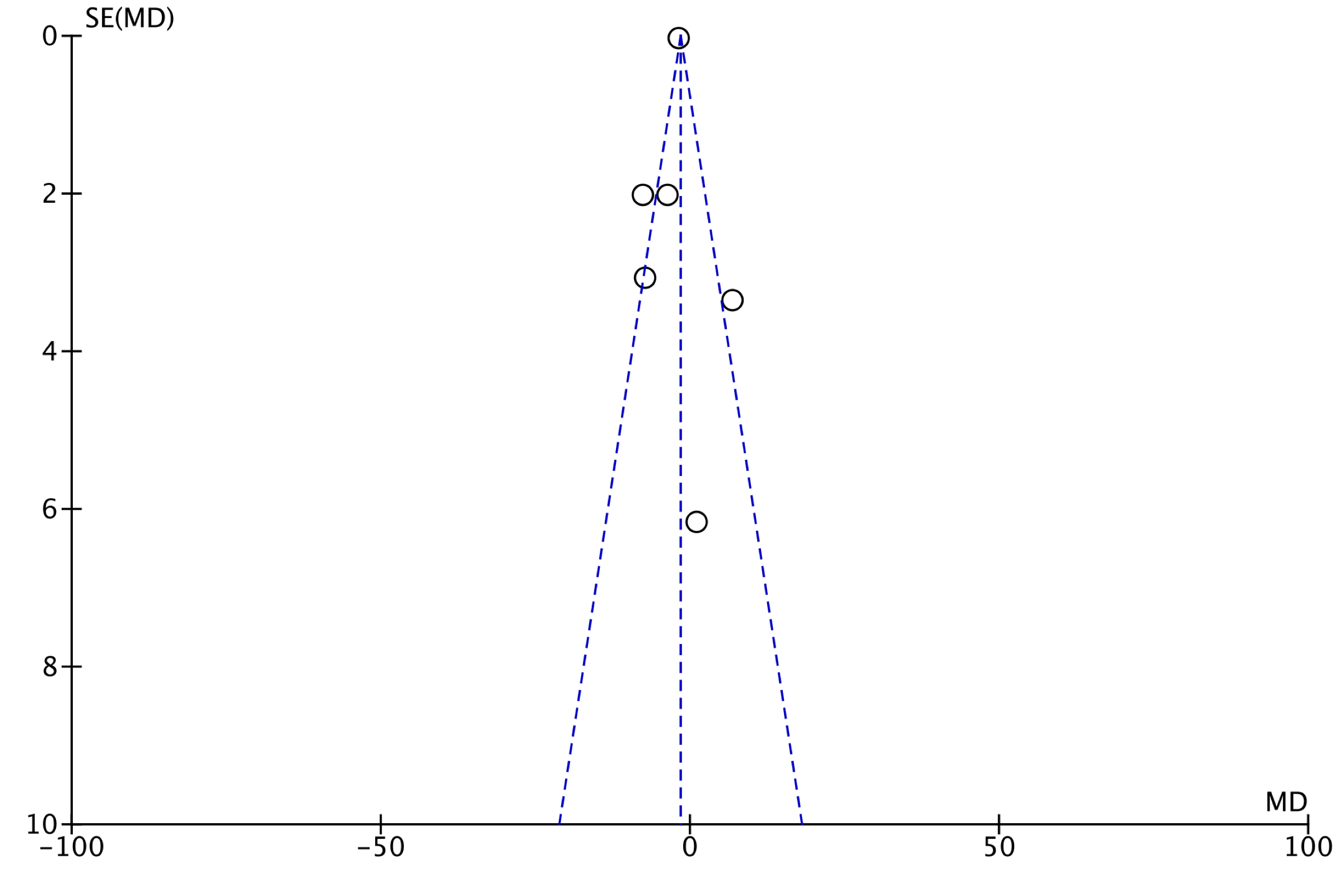


Figure S6. Funnel plots for fasting blood glucose.


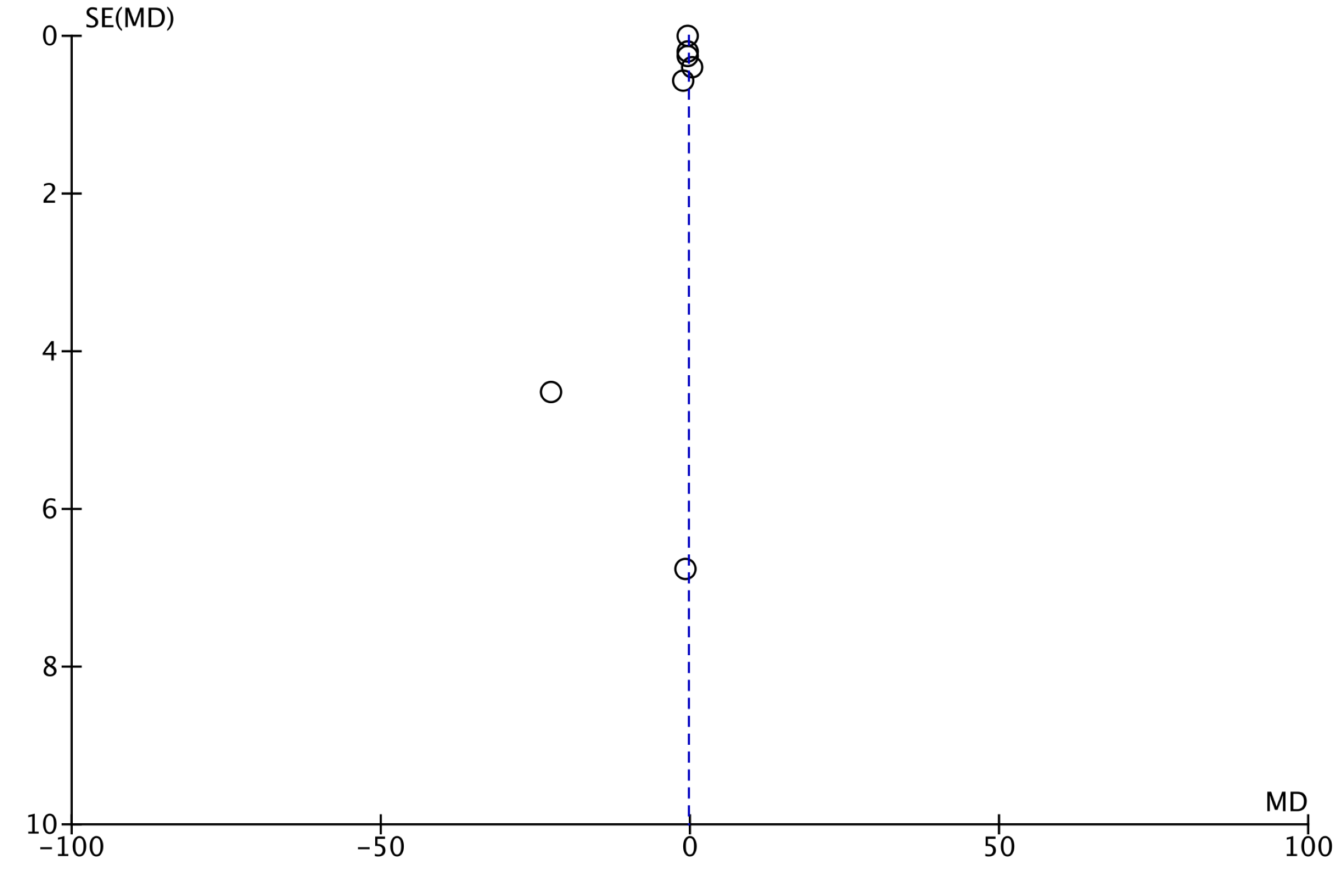


Figure S7. Funnel plots for hbA1c level.


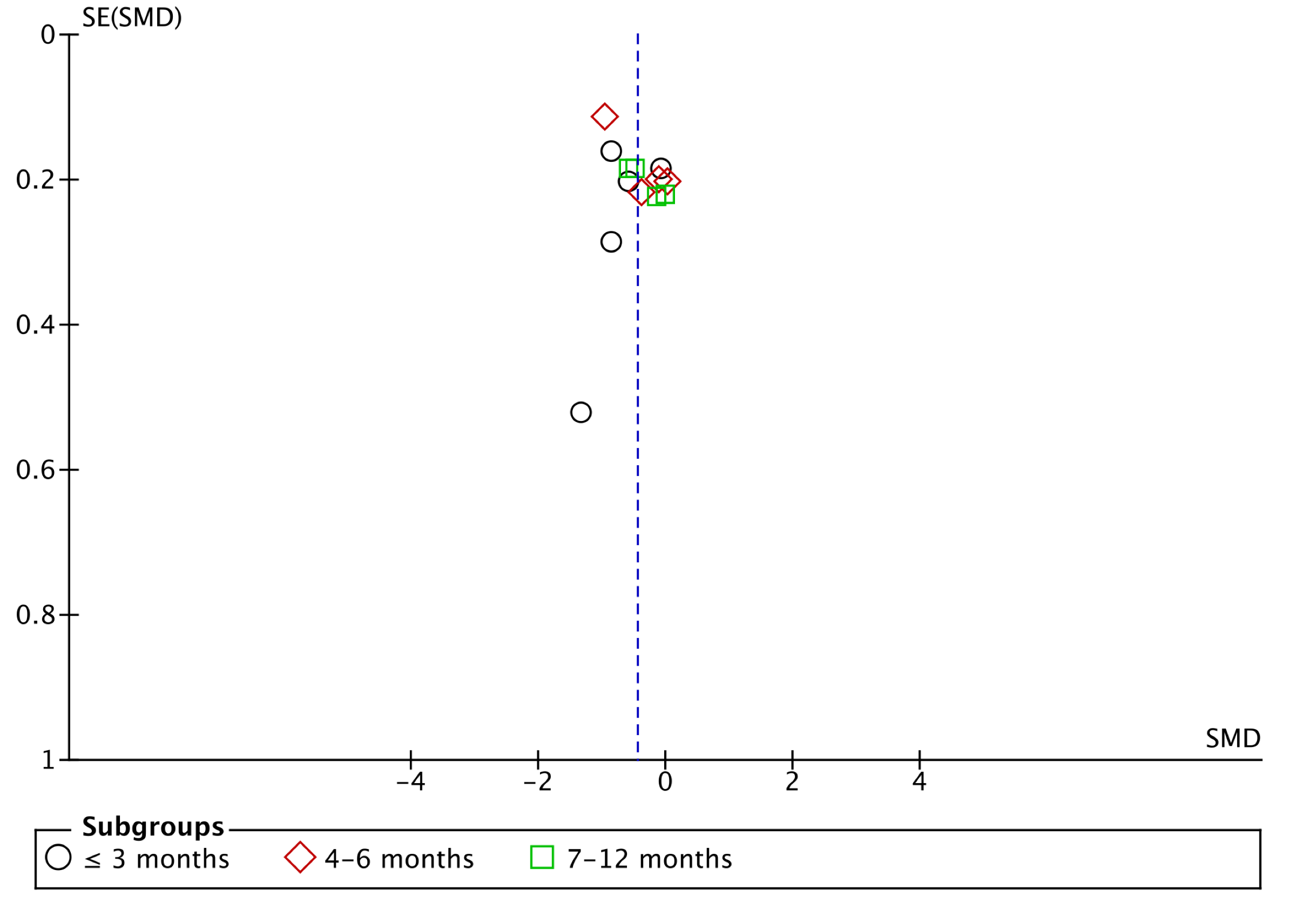


Figure S8. Funnel plots for body fat.


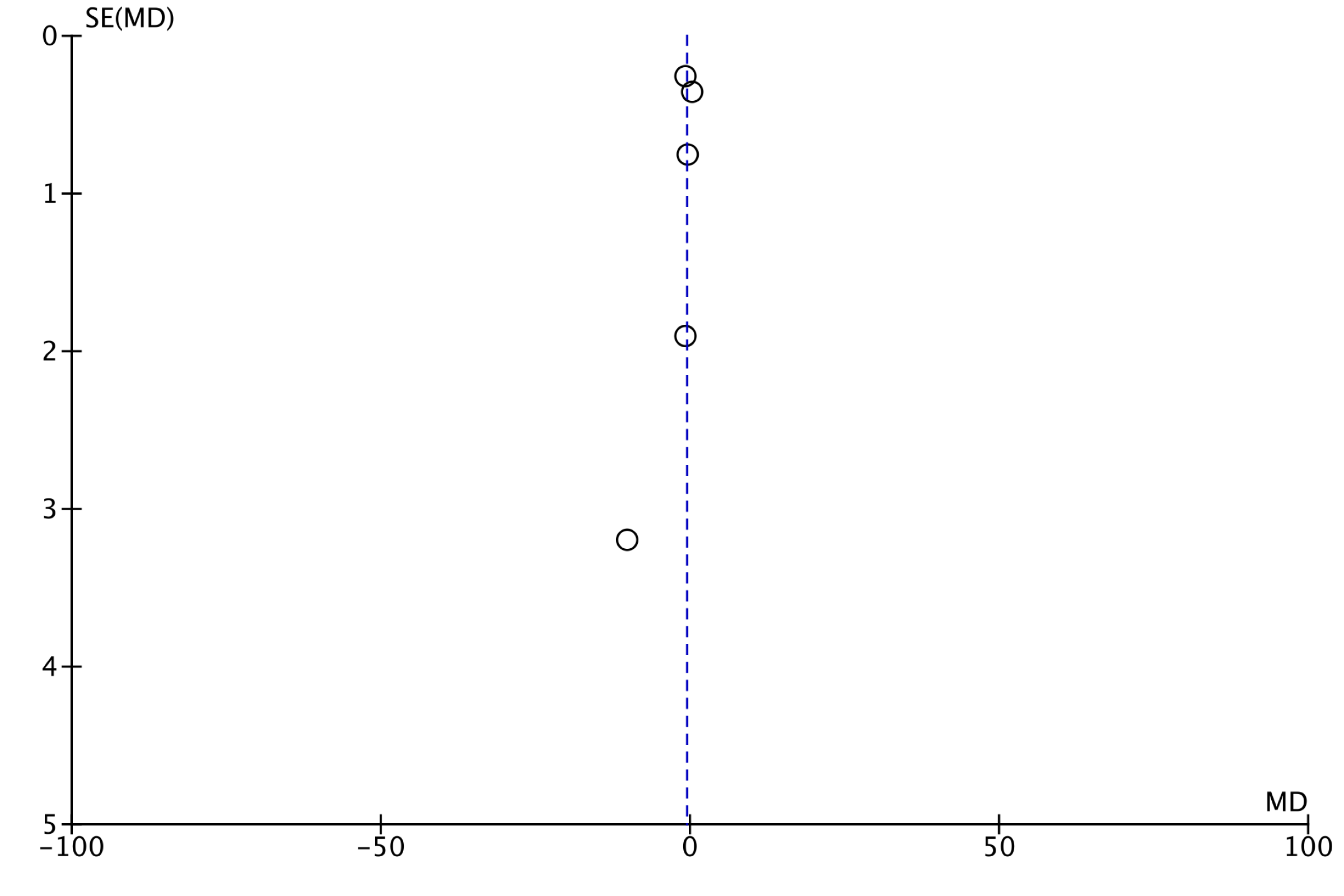


Figure S9. Funnel plots for triglyceride level.


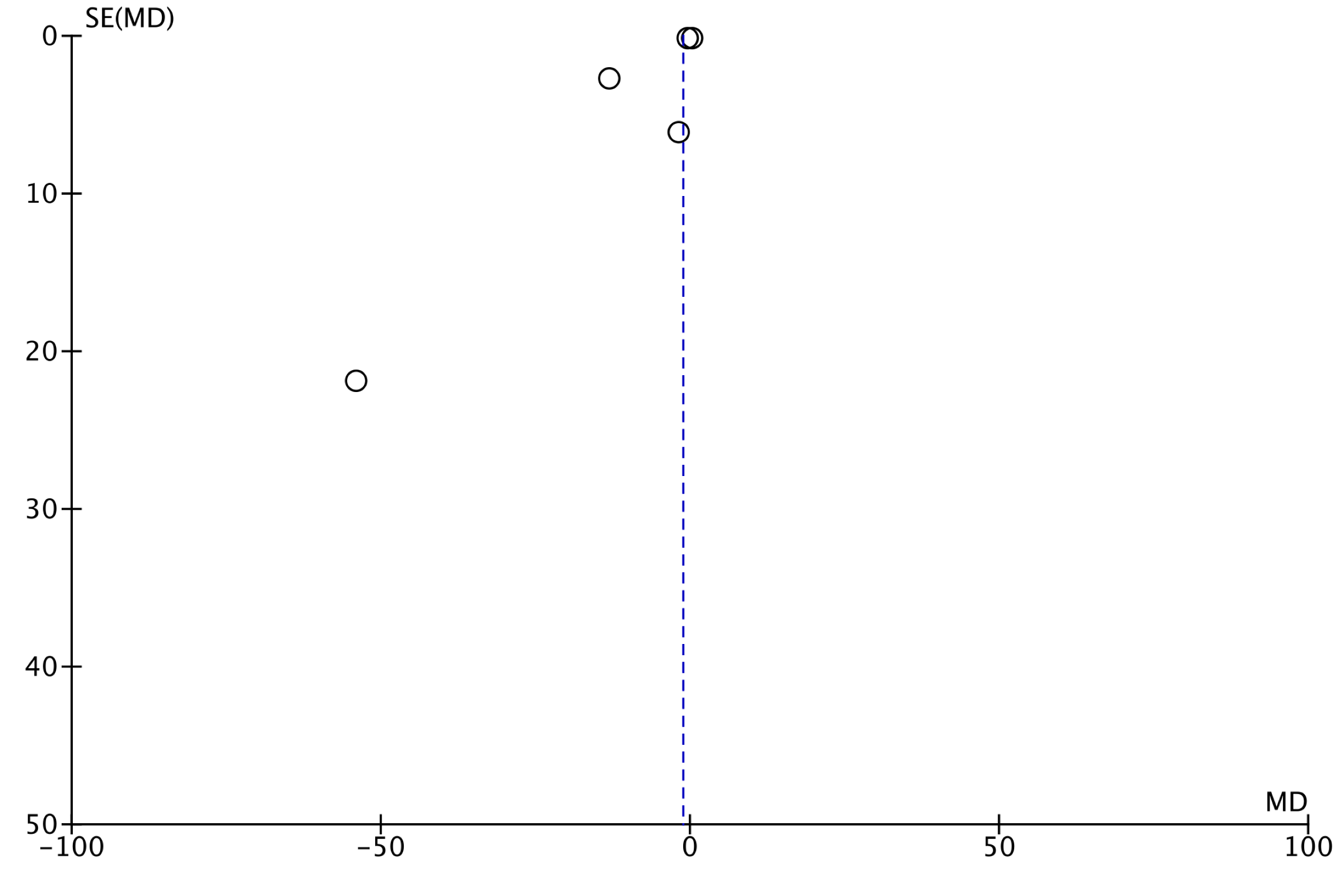


Figure S10. Funnel plots for Framingham risk.


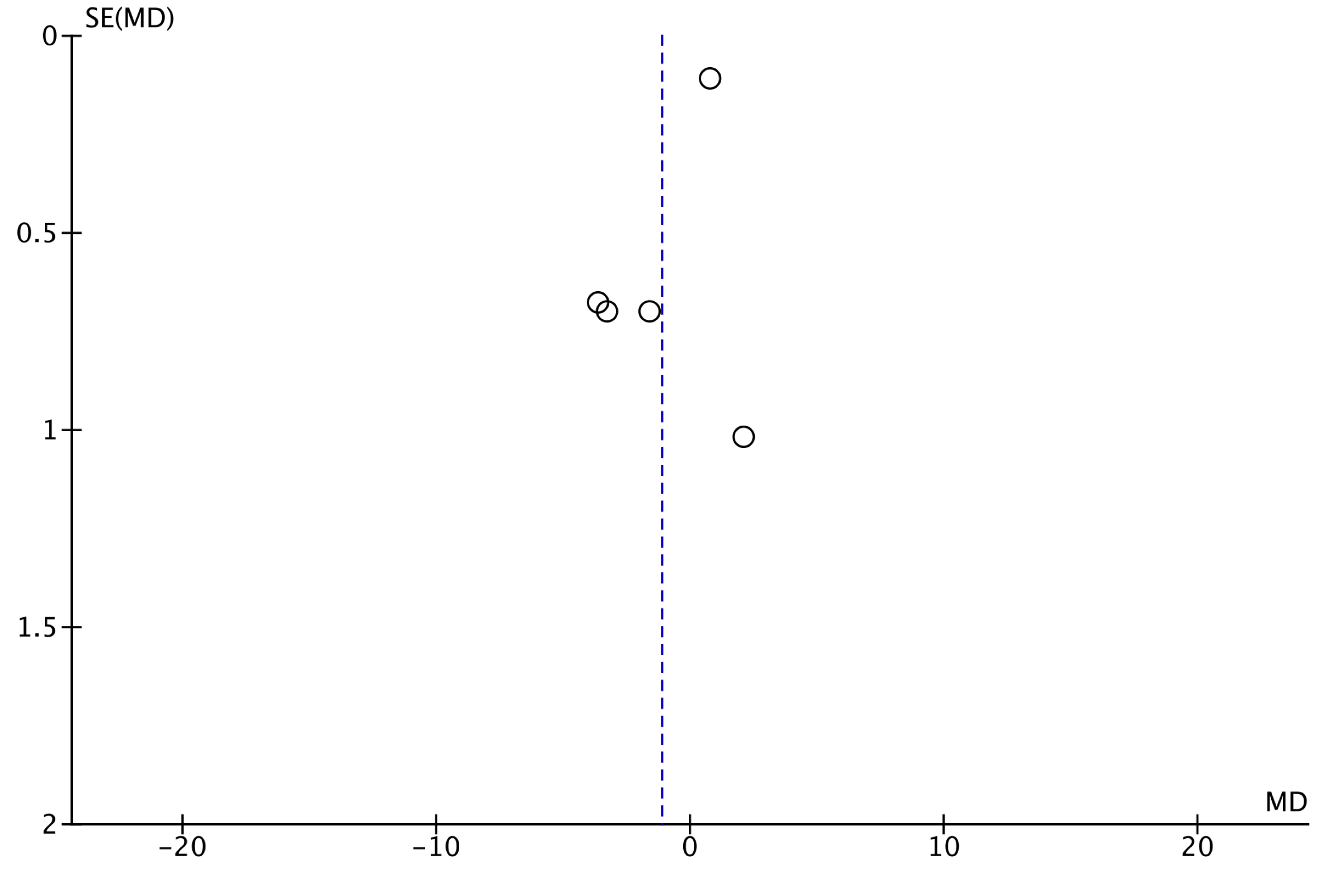


Figure S11. Funnel plots for calorie intake.


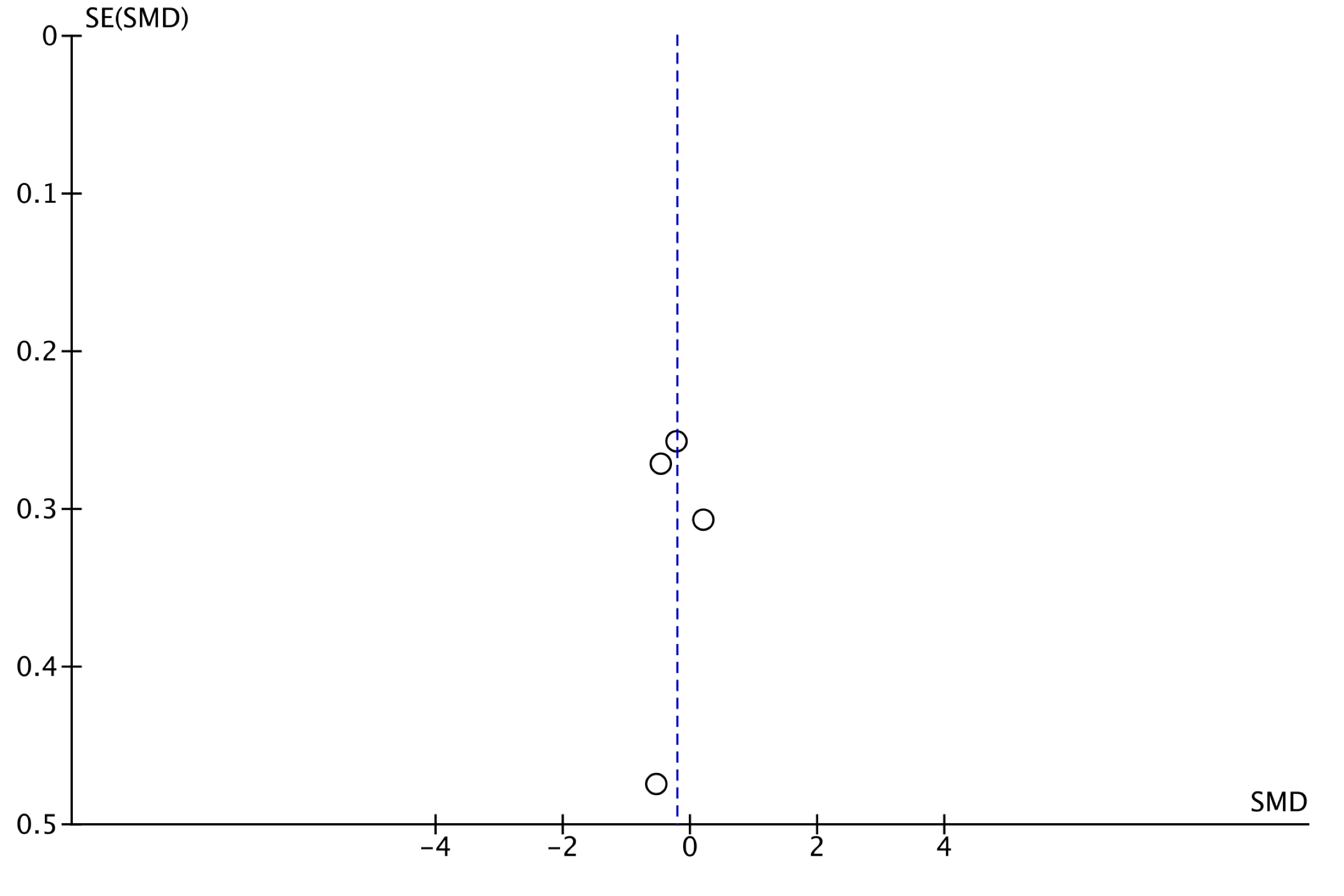


Figure S12. Funnel plots for fruit and vegetable consumption.


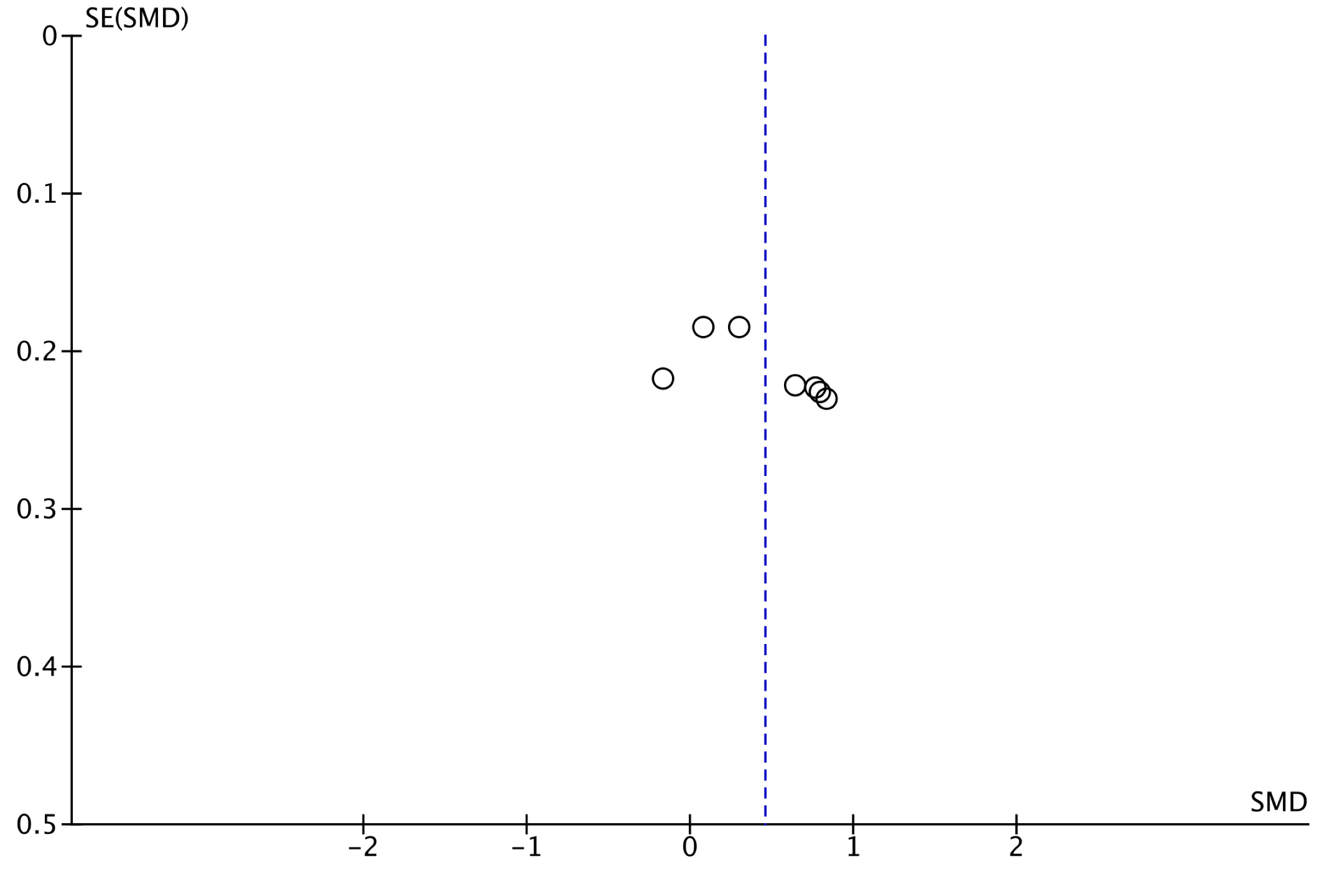

Supplement: Multimedia Appendix 4 [file jmir_v23i5e15649_app4.docx]
