# Supplementary material for: Effectiveness of eHealth Nutritional Interventions for Middle-Aged and Older Adults: Systematic Review and Meta-analysis
Source: J Med Internet Res. 2021 May 17;23(5):e15649. doi: 10.2196/15649 (PMC8167617; doi:10.2196/15649)
Supplement: Multimedia Appendix 5 [file jmir_v23i5e15649_app5.docx]

Multimedia Appendix 5. Types of features implemented by eHealth apps.

| Studies | Record health behaviours and health reports  (*n* = 50) | Reminder system  (*n* = 15) | Educational content  (*n* = 59) | Motivational messages  (*n* = 22) | Health goals setting  (*n* = 36) | Point-based system  (*n* = 3) |
| --- | --- | --- | --- | --- | --- | --- |
| Aalbers et al., 2016 |  | ✓ |  |  | ✓ |  |
| Ahn et al., 2016 | ✓ | ✓ |  |  |  |  |
| Akhu-Zaheya & Shiyab, 2016 |  |  | ✓ |  |  |  |
| Alencar et al., 2017 | ✓ |  | ✓ |  |  |  |
| Ambeba et al., 2015 | ✓ |  |  |  | ✓ |  |
| Axley et al., 2017 |  |  | ✓ | ✓ |  |  |
| Balk-Møller, 2017 | ✓ |  | ✓ | ✓ | ✓ | ✓ |
| Barnason et al., 2019 |  |  | ✓ |  |  |  |
| Benson et al., 2019 |  |  | ✓ | ✓ | ✓ |  |
| Bentley et al., 2016 | ✓ |  |  |  |  |  |
| Block et al., 2015 |  |  |  |  | ✓ |  |
| Castro Sweet et al., 2018 | ✓ |  | ✓ | ✓ | ✓ |  |
| Choi et al., 2019 | ✓ |  | ✓ | ✓ | ✓ |  |
| Clark et al., 2019 | ✓ |  | ✓ |  | ✓ |  |
| Das et al., 2017 | ✓ |  | ✓ |  | ✓ |  |
| Dennison et al., 2014 |  | ✓ | ✓ |  | ✓ |  |
| Duan et al., 2018 | ✓ | ✓ |  |  | ✓ |  |
| Duncan et al., 2014 | ✓ |  | ✓ | ✓ | ✓ |  |
| Elbert et al., 2016 |  |  | ✓ | ✓ |  |  |
| Eyles et al., 2017 | ✓ | ✓ |  |  |  |  |
| Fernandes et al., 2016 |  |  | ✓ | ✓ |  |  |
| Fukuoka et al., 2015 | ✓ | ✓ | ✓ |  | ✓ |  |
| Gilson et al., 2017 | ✓ |  | ✓ |  |  |  |
| Gomez-Marcos et al., 2018 | ✓ |  | ✓ |  |  |  |
| Haas et al., 2019 | ✓ |  | ✓ | ✓ | ✓ |  |
| Hageman et al., 2017 | ✓ |  | ✓ | ✓ |  |  |
| Haggerty et al., 2017 | ✓ | ✓ | ✓ | ✓ |  |  |
| Hales et al., 2016 | ✓ | ✓ | ✓ | ✓ | ✓ | ✓ |
| Hansel et al., 2017 | ✓ |  | ✓ |  |  |  |
| Hartman et al., 2016 | ✓ |  | ✓ |  | ✓ |  |
| Holmen et al., 2014 | ✓ |  |  | ✓ | ✓ |  |
| Huber et al., 2015 |  |  | ✓ | ✓ | ✓ |  |
| Järvelä-Reijonen et al., 2018 |  |  | ✓ |  | ✓ |  |
| Kanera et al., 2017 |  |  | ✓ |  | ✓ |  |
| Kempf et al., 2017 | ✓ |  | ✓ | ✓ |  |  |
| Khanna et al., 2014 | ✓ |  | ✓ | ✓ |  |  |
| Koot et al., 2019 | ✓ |  | ✓ |  |  |  |
| Lim et al., 2016 | ✓ | ✓ | ✓ |  |  |  |
| Liu et al., 2018 |  |  | ✓ |  | ✓ |  |
| Lorig et al., 2016 | ✓ |  | ✓ |  | ✓ |  |
| Luley et al., 2014 | ✓ |  | ✓ |  |  |  |
| Mason et al., 2018 | ✓ | ✓ | ✓ | ✓ | ✓ |  |
| Martin et al., 2015 | ✓ |  | ✓ |  |  |  |
| McCarroll et al., 2015 | ✓ | ✓ |  |  |  |  |
| McKenzie et al., 2017 | ✓ |  | ✓ |  |  |  |
| Michaelides et al., 2018 | ✓ | ✓ | ✓ | ✓ | ✓ |  |
| Miller et al., 2017 | ✓ |  | ✓ |  | ✓ |  |
| Moin et al., 2018 | ✓ |  | ✓ |  | ✓ |  |
| Mundi et al., 2015 |  |  | ✓ | ✓ |  |  |
| Nepper et al., 2019 |  |  | ✓ |  |  |  |
| Nolan et al., 2018 | ✓ |  | ✓ | ✓ | ✓ |  |
| Orlandoni et al., 2016 | ✓ |  |  |  |  |  |
| Peimani et al., 2015 |  |  | ✓ |  |  |  |
| Pfaeffli Dale et al., 2015 |  |  | ✓ |  |  |  |
| Ramadas et al., 2018 |  | ✓ | ✓ |  |  |  |
| Recio-Rodriguez et al., 2016 | ✓ |  | ✓ |  | ✓ |  |
| Recio-Rodriguez et al., 2018 | ✓ |  | ✓ |  | ✓ |  |
| Santo et al., 2018 |  |  | ✓ | ✓ |  |  |
| Saslow et al., 2017 | ✓ |  | ✓ |  | ✓ |  |
| Saslow et al., 2018 | ✓ |  | ✓ | ✓ | ✓ |  |
| Sepah et al., 2017 | ✓ |  | ✓ |  | ✓ |  |
| Shahid et al., 2014 |  |  | ✓ |  |  |  |
| Sun et al., 2019 | ✓ | ✓ | ✓ |  |  |  |
| Vadheim et al., 2017 | ✓ |  | ✓ |  | ✓ |  |
| van Doorn-van Atten et al., 2018 ) | ✓ |  | ✓ |  |  |  |
| Ventura Marra et al., 2019 | ✓ |  | ✓ |  | ✓ |  |
| Waki et al., 2014 | ✓ |  | ✓ |  |  |  |
| Wayne et al., 2015 | ✓ |  |  |  | ✓ |  |
| Whitelock et al., 2019 | ✓ | ✓ | ✓ |  |  | ✓ |
| Zhou et al., 2016 | ✓ |  | ✓ |  | ✓ |  |
