# Supplementary material for: Effectiveness of eHealth Nutritional Interventions for Middle-Aged and Older Adults: Systematic Review and Meta-analysis
Source: J Med Internet Res. 2021 May 17;23(5):e15649. doi: 10.2196/15649 (PMC8167617; doi:10.2196/15649)
Supplement: Multimedia Appendix 7 [file jmir_v23i5e15649_app7.docx]

Multimedia Appendix 7. Comprehensive overview of studies included in systematic review.

| Aalbers et al (2016). Netherlands ^72^. | | | | | |
| --- | --- | --- | --- | --- | --- |
| *Study Design* | *Participant Information* | *Intervention description* | | *Outcome measures* | *Results* |
| *Design*  2-group pre-post study  *Group Info*   - Goal-setting group (GS),  n = 1212 - Non-goal setting group (NG), n = 1093   *Duration*  12 months    *Measurement points*  Baseline, 12 months,    *Attrition rate*  Did not specify | *N* = 2305  *Sample*  Healthy adults  *Mean age*   - GS = 52.34 (12.21) - CG = 51.28 (13.73)     *Gender distribution*   - GS = 71.11% female (862) - NG = 63.04% female (698) | *Intervention*  All participants played games on the app to assess their cognitive function. The GS group set lifestyle goals, were sent feedback about their goals, and monitored the goal achievements. Weekly blogs on heath and healthy recipes were also featured.  *eHealth features*  Games and goal setting functions on mobile application. | | *Primary outcomes*   - Lifestyle-specific goal-setting success rate   *Secondary outcomes*   - Weight - BMI | *Primary outcomes*  Of the 392 GS participants at follow-up, they achieved 38.75% of their health goals.  *Secondary outcomes*  Of the 153 GS participants at follow-up, there were significant weight loss (mean Δ = -0.8kg, *P*=.01), BMI (mean Δ = -0.3, *P*=.01), and reduction in unhealthy nutritional behaviour (mean Δ = -0.6, *P*=.01) from baseline. |
| Ahn et al (2016), South Korea ^32^. | | | | | |
| *Study Design* | *Participant Information* | *Intervention description* | | *Outcome measures* | *Results* |
| *Design*  2-group pre-post study  *Group Info*   - Intervention group (IG),  n = 14 - Control group (CG), n = 12   *Duration*  1 month  *Measurement points*  Baseline, 1 month  *Attrition rate*  0% | *N* = 26  *Sample*  Patients with diabetes mellitus  *Mean age*   - IG = 50.5 (17.1) - CG = 49.7 (16.4)   *Gender distribution*   - IG = 57.1% female - CG = 41.7% female | *Intervention*  IG recorded their daily dietary intake and the program displays their dietary patterns and results of their nutrient intake. The program has an alarm to remind IG to input their dietary data. CG received weekly nutrition classes for 1 month.  *eHealth features*  Web- and mobile-based program, Diabetes Mellitus Dietary Management Guide | | *Primary outcomes*   - Weight - BMI - Dietary attitude measured by 13-item survey devised by authors - Dietary behaviour measured by 19-item survey devised by authors - Nutritional knowledge measured by 20-item survey devised by authors | *Primary outcomes*  No changes in weight and BMI for both groups post intervention.  Dietary attitude scores improved in both IG (mean Δ = 2.4, *P*<.05) and CG (mean Δ = 3.3, *P*<.05).  Dietary behaviours improved for IG (mean Δ = 4.7, *P*<.01), but not CG (*P*>.05).  There were no improvements in nutritional knowledge for both groups (*P*>.05). |
| Akhu-Zaheya & Shiyab (2016), Jordan ^42^. | | | | | |
| *Study Design* | *Participant Information* | *Intervention description* | | *Outcome measures* | *Results* |
| *Design*  3-group randomized controlled trial (RCT)  *Group Info*   - IG, *n* = 52, received reminder text messages - CG, *n* = 56, received usual care - Placebo, *n* = 52, received general text messages   *Duration*  3 months  *Measurement points*  Baseline, 3 months  *Attrition rates*  11.11% | *N* = 160    *Sample*  Patients with cardiovascular diseases (CVD)  *Mean age*  54.94 (10.86)  *Gender distribution*  45.6% female | *Intervention*  In addition to routine care, IG received reminder text messages for medication, smoking cessation, and diet. Messages about diet included the benefits and importance of a healthy diet, unhealthy foods to avoid and the negative consequences of unhealthy diet.  *eHealth features*  Text messages via mobile phones | | *Primary outcomes*   - Mediterranean Diet Adherence Screener (MEDAS) - Morisky 8-Item Medication Adherence Scale (MMAS) - Readiness to Quit Smoking Ladder - Number of cigarettes smoked | *Primary outcomes*  At 3 months, post-hoc analysis showed IG scored highest on MEDAS, followed by placebo, and CG, with scores 8.86 (1.8), 8.11 (2.03), and 5.80 (1.97) respectively, *P* <.001. Thus, they had the greatest adherence to the Mediterranean diet. IG scored highest for MMAS, followed by placebo and CG, with scores 6.29 (1.03), 5.56 (1.48), 5.24 (1.66) respectively, *P*=.001. However, no post-hoc results to reveal where the group differences in MMAS lies. No group differences in readiness to quit smoking (*P*=0.33) and cigarette numbers (*P*=0.34).  Within-group comparisons of MEDAS from baseline to 3 months were found to be increased in IG [6.9 (1.9) vs 8.86 (1.8), *P*<.001] and reduced in CG [7.28 (2.0) vs 5.8 (1.97), *P*<.001]. |
| Alencar et al (2017), United States ^89^. | | | | | |
| *Study Design* | *Participant Information* | | *Intervention description* | *Outcome measures* | *Results* |
| *Design*  2-group RCT  *Group Info*   - Intervention group,  n = 13 - Comparison group, n = 12   *Duration*  12 weeks    *Measurement points*  Baseline, 12 weeks    *Attrition rate*  5 drop outs (2 IG, 3 CG); 16.7% | *N* = 25  *Sample*  Obese participants  *Mean age*   - IG = 41.2 (13.9) - CG = 52.4 (23.9)     *Gender distribution*   - Female = 52% (13) - Male = 48% (12) | | *Intervention*  Telemedicine-based (video conferencing) weight loss program.  Weekly educational video module and handout. Individual feedback from dietitian. Nutrition education, fitness education, behaviour modification  *eHealth features*  Bluetooth wireless mHealth devices (body composition scale, activity tracker, accelerometer, and blood pressure cuff), mobile phone-based health coaching, video conferencing | *Primary outcomes*   - Weight - BMI - Body fat - Weekly step change - Blood pressure | *Primary outcomes*  Compared to baseline, IG had significantly more reductions in weight and body fat as compared to CG (*P*<.05):  Body weight loss*:*  IG = [mean Δ 7.3 (+/-4.4kg)],  CG = [mean Δ 1.3 (+/3.9kg)]  Percent body weight loss:  IG = [mean percent Δ 7.2 (+/-4.4%)],  CG = [mean percent Δ 1.5 (+/-4.1%)]  Body Fat reduction*:*  IG = [mean Δ -9.0 (+/8.3)],  CG = [mean Δ 1.3 (+/3.9)]  IG significantly increased their weekly step compared to CG (*P* <.05)*:*  IG = [mean Δ 30,163.8 (+/30,117.6)],  CG = [mean Δ -5972.0 (+/22,286.0)]  Compared to baseline, no significant changes in BP for both groups (*P*>.05).  Systolic BP (mmHg):  IG = [mean Δ 3.4 (+/7.0)],  CG = [mean Δ -3.3 (+/9.5)]  Diastolic BP (mmHg):  IG = [mean Δ 0.62 (+/4.0)],  CG = [mean Δ -4.3 (+/8.3)] |
| Ambeba et al (2015), United States ^90^. | | | | | |
| *Study Design* | *Participant Information* | | *Intervention description* | *Outcome measures* | *Results* |
| *Design*  2-group RCT  *Group Info*   - IG,  n = 70 - CG, n = 140   *Duration*  24 months    *Measurement points*  Baseline, 6, 12, 18 and 24 months; 2 x 24-hour dietary recalls on one weekday and one weekend day    *Attrition rate*  IG=11 (15.7%) CG=19 (13.6%) | *N* = 210  *Sample*  Obese adults  *Mean age*   - IG = 46.4 (9.4) - CG = 47.0 (8.8)     *Gender distribution*   - IG = 84.3% female - CG = 85% female | | *Intervention*  Behavioural weight loss treatment. Daily feedback messages tailored to participant’s self-monitoring entries for energy and fat intake, delivered remotely in real-time.  Group sessions, dietary and exercise goals, self-monitoring, and feedback messages.  *eHealth features*  IG had PDAs to record dietary intake, and receive feedback | *Primary outcomes*   - Dietary intake | *Primary outcomes*  Percent change in energy intake over time;  Significant within-group decreases in energy (IG: −22.8%, *P*<.0001; CG: −14.0%, *P*<.0001) and total fat intake (IG: −10.4%, *P*=.0002; CG: −4.7%, *P*=.02) over time compared to baseline.  Compared to CG, the IG achieved a larger mean reduction in energy intake, mainly at 6 months (IG: −26.5%; CG: −15.0%, *P*=.01) and 24 months (IG: −23.4%; CG: −14.1%, *P*=.03).  Percent change in saturated fat intake over time;  IG had a significant within-group decrease in saturated fat intake (IG: −11.3%, *P*=.005; CG: -0.5%, *P*=.03) over time.  IG had a reduction in saturated fat intake at 24 months compared to CG (IG: −9.3%; CG: 3.4%, *P*=.04),  Percent change in carbohydrate intake over time  Significant improvements over time in carbohydrate intake for both groups (*P*<.05).  Percent change in total fat intake over time  Significant improvements over time in total fat intake for both groups (*P*<.05). Trend toward significant decrease at 6 months (IG: −15.6%; CG: −8.2%, *P*=.06).  No difference in added sugar intake between IG and CG. Significant within-group difference; IG significantly reduced added sugar intake from baseline to 24 months (IG: −25.8%, *P*=.01; CG: −11.5%, *P*=.10). |
| Axley et al (2017), United States ^43^. | | | | | |
| *Study Design* | *Participant Information* | *Intervention description* | | *Outcome measures* | *Results* |
| *Design*  2-group RCT (pilot)  *Group Info*   - IG, *n* = 13 - CG, *n* = 17     *Duration*  6 months  *Measurement points*  Baseline, 6 months  *Attrition rate*  27% | *N* = 30  *Sample*  Patients with chronic liver diseases  *Mean age*   - IG = 54 (2.7) - CG = 52 (2.3)   *Gender distribution*  67% female | *Intervention*  3 weekly text messages sent by CareMessage. Text messages that included educational content on nutrition, exercise, and stress management. Motivational messages to encourage them to maintain health behaviours. For 2 weeks, messages were targeted on nutrition; encouraging participants to increase water consumption and reduce intake of sugary drinks. During the last 3 weeks, messages provided online links to view exercises routines and healthy recipes.  CG received usual care.  *eHealth features*  Text messaging via mobile phones. | | *Primary outcomes*   - Weight - BMI     *Secondary outcomes*   - Liver enzyme and lipid profile | *Primary outcomes*  Significant reduction in weight [mean Δ = 6.9 (+/-2.8), *P*=.03], and BMI in IG, [mean Δ=1, *P*= .025]. No changes in weight and BMI for CG (*P*>.05).  *Secondary outcomes*  Significant mean reduction in liver enzymes ALT levels(IU/L), [mean Δ=12.5 (+/-5.3), *P*=.035], and AST levels (IU/L) [mean Δ=9.3(+/-4.2), *P*=.048] in IG.  No changes in CG in both primary and secondary outcomes (*P*>.05 for all). |
| Balk-Møller et al (2017), Denmark ^73^. | | | | | |
| *Study Design* | *Participant Information* | *Intervention description* | | *Outcome measures* | *Results* |
| *Design*  2-group RCT  *Group Info*   - IG, *n* = 355 - CG, *n* = 211   *Duration*  9.5 months  *Measurement points*  Baseline, 4 months, 9.5 months  *Attrition rate*  At 4 months:   - IG = 37% attrition from baseline - CG = 33% attrition from baseline     At 9.5 months:   - IG = 33% attrition from 4 months - CG = 18% attrition from 4 months | *N* = 566  (For analysis, 152 from IG and 117 from CG)  *Sample*  Healthy older adults  *Mean age*   - IG = 47.0 (10.0) - CG = 47.0 (9.9)   *Gender distribution*   - IG = 92.1% female - CG = 92.3% female | *Intervention*  IG pledged a health goal they aimed to achieve. The app provided feedback, content, and messages that helped IG to focus and achieve health goals. IG entered daily food intake and exercise levels into the app. There were weekly assignments to encourage IG to work together and achieve health goals as part of a group competition, earn points on the app, and win prizes. It encouraged users to work together to complete challenges, thereby facilitating social support and social interactions in real life.  CG had no activities throughout the study.  *eHealth features*  A web- and app-based application named SoSu-life, which allowed users to enter daily dietary intake and exercise. It provided personalised feedback, included a points system, and initiated weekly assignments. | | *Primary outcomes*   - Weight   *Secondary outcomes*   - Body fat percentage - Waist circumference (WC) - Blood pressure (BP) level - Cholesterol level | *Primary outcomes*  At 9.5 months, IG had greater reduction in body weight (adjusted difference = -1.01kg, 95% CI: -1.94 to -.08, *P*=.03) as compared to CG.  *Secondary outcomes*  At 9.5 months, IG had greater reduction in body fat percentage, (adjusted difference = -.79, 95% CI: -1.48 to -.09, *P*=.03), and WC (adjusted difference = -1.79cm, 95% CI: -3/09 to -.049, *P*=.007) as compared to CG.  There were no differences in BP and cholesterol levels between groups at 4 months and 9.5 months. |
| Barnason et al (2019), United States ^44^. | | | | | |
| *Study Design* | *Participant Information* | *Intervention description* | | *Outcome measures* | *Results* |
| *Design*  2-group RCT    *Group Info*   - Intervention group (IG),  n = 22 - Control group (CG), n = 21   *Duration*  3 months    *Measurement points*  Baseline, 4 months, 4 months    *Attrition rate*  13.95% | *N* = 43  *Sample*  Patients with cardiovascular diseases    *Mean age*  63 (9.3)  *Gender distribution*   - IG = 23% (5) female - CG = 38% female (8) | *Intervention*  All participants received standard cardiac rehabilitation. IG were educated on caloric intake, and portion control guidelines. 36 telehealth sessions with a nurse were conducted over a 6-week period.  *eHealth features*  Telephone calls | | *Primary outcomes*   - Weight - BMI   *Secondary outcomes*   - PA levels - Perceived self-efficacy - Weight management behaviours | *Primary outcomes*  At 6 months, weight loss was greater in IG (mean Δ= -7.17kg) than CG (mean Δ= -4.22kg) compared to baseline. There was also greater reduction in BMI in IG (mean Δ=2.4) than CG (mean Δ = 1.5).  *Secondary outcomes*  There were no changes in PA levels for both groups. At follow-up, IG had higher perceived self-efficacy in eating habits ([F(1,36) = 12.6, *P*=0.001] and managing diet behaviour [[F(1,36) = 8.6, *P*=0.006]. |
| Benson et al (2019), United States ^45^. | | | | | |
| *Study Design* | *Participant Information* | *Intervention description* | | *Outcome measures* | *Results* |
| *Design*  2-group RCT    *Group Info*   - IG, *n* = 60 - CG, *n* = 58   *Duration*  1 month    *Measurement points*  Baseline, 12 months    *Attrition rate*  11.9% | *N* = 118    *Sample*  Patients with type 2 diabetes  *Mean age*   - IG = 59.8 (10.2) - CG = 60 (8.66)   *Gender distribution*   - Intervention = 46.7% female - CG = 43.1% female | *Intervention*  IG received telephone coaching which aimed to motivate, educate, help participants set goals, and identify barriers in achieving a healthy lifestyle. Nutritional intervention included educating them on portion control, calorie reduction, increasing fruit, veg, and health fats intake.  *eHealth features*  Telephone calls | | *Primary outcomes*   - HbA1c level - Blood pressure level - Tobacco use - Statin and aspirin use - Adherence to Mediterranean diet     *Secondary outcomes*   - PA level - Dietary intake - BMI - LDL- cholesterol level - Medication adherence | *Primary outcomes*  At 1-year follow-up, IG increased statin and aspirin use compared to baseline (both *P*<.05). There was an increase in aspirin intake and hbA1c level in CG during follow-up (both *P*<.05). Both IG and CG improved their adherence to a mediterranean diet overtime, but no between-group differences. No other changes were observed, and there were no between-group differences at 1-year follow up.  *Secondary outcomes*  At 1 year, IG increased fruit intake and diabetes medication adherence, and reduced hbA1c level and LDL-cholesterol level (all *P*<.05). CG reduced whole grains intake, hbA1c level, and increased cholesterol medication adherence (*P*<.05). No other changes were observed. |
| Bentley et al (2016), United Kingdom ^74^. | | | | | |
| *Study Design* | *Participant Information* | *Intervention description* | | *Outcome measures* | *Results* |
| *Design*  3-group RCT  *Group Info*   - Group 1, *n* = 9, received advice on diet and exercise - Group 2, *n* = 9, group 1 intervention and use of AiperMotion 500 - Group 3, *n* = 9, group 2 intervention and email motivational support   *Duration*  3 months  *Measurement points*  Baseline, 6 weeks, 12 weeks, 16 weeks  *Attrition rate*  26% | *N* = 27    *Sample*  Patients with diabetes mellitus (Type 2)  *Mean age*   - Group 1 = 55.7 (9.9) - Group 2 = 52.1 (7.8) - Group 3 = 50.8 (8.1)   *Gender distribution*   - Group 1 = 100% female - Group 2 = 44.4% female - Group 3 = 22.2 % female | *Intervention*  All participants received 90-min group training on weight loss and self-management of HbA1c level. They were encouraged to have a low-GI diet. Group 2 and 3 received additional 60-min training on use of AiperMotion 500. Group 3 recorded event diaries detailing events that might influence their conformance to lose weight.  *eHealth features*  Wearable device, AiperMotion 500, which tracks PA levels and allows users to input nutritional intake such as food type, portion sizes, or number of calories. | | *Primary outcomes*   - Acceptance, adherence, and satisfaction towards AiperMotion 500   *Secondary outcomes*   - Weight - Glycated haemoglobin A1c level (HbA1c) | *Primary outcomes*  Adherence to use the AiperMotion 500 increased over time. Qualitative analysis showed that the device was generally easy to use, well-received and acceptable, and participants were satisfied to use it.  *Secondary outcomes*  From week 6 to 16, there was an increase of weight in Group 1 (mean Δ = 0.7), and decrease of weight in Group 2 (mean Δ = -3.3) and Group 3 (mean Δ = -3.0). There was an increase of HbA1c level in Group 1 (mean Δ = 0.9), and decrease of HbA1c level in group 2 (mean Δ = -10.7) and Group 3 (mean Δ = -5.0). |
| Block et al (2015). United States ^46^. | | | | | |
| *Study Design* | *Participant Information* | *Intervention description* | | *Outcome measures* | *Results* |
| *Design*  2-group RCT  *Group Info*   - IG, *n* = 33 - CG, *n* = 33   *Duration*  6 months  *Measurement points*  Baseline, 3 months, 6 months  *Attrition rate*  15% | *N* = 339    *Sample*  Patients with prediabetes  *Mean age*   - Intervention = 55.0 (8.8) - CG = 54.9 (9.1)   *Gender distribution*   - Intervention = 31.9% female - CG = 30.7% female | *Intervention*  IG had weekly goal settings which aimed to improve dietary habits, such as increasing dietary fibre consumption, and decreasing saturated and trans fats, added sugars food, and refined carbohydrates.  *eHealth features*  Alive-PD, automated program which provides behaviour change program. | | *Primary outcomes*   - Fasting glucose level - HbA1c level     *Secondary outcomes*   - Weight - BMI - WC - TG/HDL ratio - Framingham diabetes risk score | *Primary outcomes*  Compared to CG, IG had greater decrease in fasting glucose (mean = –7.36 mg/dL, 95% CI –7.85 to –6.87, *P*<.001) and HbA1c (mean = –0.26%, 95% CI –0.27 to –0.24, *P*<.001).  *Secondary outcomes*  Compared to CG, IG had greater decrease in weight (mean = 3.26 kg, 95% CI –3.26 to –3.25, *P* <.001), BMI (mean = 1.05, 95% CI –1.06 to –1.05, *P*<.001), and WC (mean = 4.56, 95% CI –4.69 to –4.43, *P*<.001). Reduction in Framingham diabetes risk score in IG from 16% at baseline to 11% at 6 months (95% CI 10.08 to 11.92, *P*<.001) |
| Castro Sweet et al (2018), United States ^47^. | | | | | |
| *Study Design* | *Participant Information* | *Intervention description* | | *Outcome measures* | *Results* |
| *Design*  Single-arm pre-post study    *Group Info*  All participants took part in the intervention  *Duration*  12 months    *Measurement points*  Baseline, 4 months, 6 months, 12 months  *Attrition rate*  0% | *N* = 501    *Sample*  Adults at risk of diabetes  *Mean age*  68.8 (2.58)    *Gender distribution*  64% female (320) | *Intervention*  The programme involves a curriculum for participants to access weekly lessons, set health goals, track their progress (weight, PA and dietary intake), and interact with other users. Health coaches can monitor user’s progress and provide them with personalised feedback.  *eHealth features*  Website and mobile app to access the programme, pedometer to track PA levels | | *Primary outcomes*   - Weight - hbA1c level - Total cholesterol level - World Health Organization Well-being index (WHO-5) - Patient Health Questionnaire for depression and anxiety (PHQ-4) - Summary of Diabetes Self-care Activities (SDSCA) | *Primary outcomes*  At baseline, the mean body weight was 207.7 (38.4) lbs. Compared with baseline, there was a reduction of 6.5% of mean weight (*P*=.001), reduction of 8.0% (*P*=.001) at 6 months, and reduction of 7.5% (*P*=.001) at 12 months.  At 6 months, there was a reduction in hbA1c level by 0.14% (n=69, *P*=.0001), and reduction in cholesterol by 12.92mg/dL (n=136, *P*=.0001) compared with baseline. At 12 months, reduction in hbA1c level was 0.14% (*P*=.0001), and cholesterol was reduced by 7.08mg/dL (*P*=.008). Self-reported well-being, depression, and self-care improved (*P*<.0001). |
| Choi et al (2019), United States ^48^. | | | | | |
| *Study Design* | *Participant Information* | *Intervention description* | | *Outcome measures* | *Results* |
| *Design*  2-group pilot RCT  *Group Info*   - IG, *n* = 51 - CG, *n* = 49   *Duration*  6 months  *Measurement points*  Baseline, 3 months, 6 months  *Attrition rate*  12% | *N* = 100    *Sample*  Patients with cardiovascular diseases  *Mean age*   - IG = 56.6 (1.7) - CG =57.2 (1.8)     *Gender distribution*   - Intervention = 43.1% female (22) - CG = 34.7% female (17) | *Intervention*  IG used an app which allowed them to set goals, challenges, have access to educational content, enter dietary intake and receive feedback. CG received standard care which involved dietary counselling.  *eHealth features*  Mobile app that allows users to take pictures and log in dietary intake, and load educational content. | | *Primary outcomes*   - Mediterranean diet score (MDS)     *Secondary outcomes*   - Blood pressure level - Cholesterol level - Triglycerides level - hbA1c level - C-reactive protein - Weight - BMI | *Primary outcomes*  At 6 months, both IG and CG improved their MDS (both *P*<.001), but there were no between group differences (*P*=.29).  *Secondary outcomes*  At 6 months, both IG and CG reduced their BMI compared with baseline (-0.8 in IG and -0.3 in CG, both *P*=.02). Between-group comparisons showed that IG had greater reduction (*P*=.03) than CG. Both IG and CG reduced their weight (-3.3 pounds in IG and -3.1 pounds in CG, both *P*=.02), but IG had greater reduction in weight than CG (*P*=.04).  No changes or between-group differences were observed in the other outcomes. |
| Clark et al (2019), United States ^49^. | | | | | |
| *Study Design* | *Participant Information* | *Intervention description* | | *Outcome measures* | *Results* |
| *Design*  3-group RCT  *Group Info*   - Video-conferencing group (VCG),  n = 50 - In-person intervention (IPG), n = 49 - Control group (CG), n = 51   *Duration*  5 months    *Measurement points*  Baseline, 6 months, 12 months    *Attrition rate*  16% | *N* = 150  *Sample*  Healthy adults  *Mean age*   - VCG = 53.2 (6.1) - IPG = 53.2 (8.1) - CG = 53.9 (6.1)     *Gender distribution*   - VCG = 92% female (46)   IPG = 77.6% female (38)   - CG = 76.5% female (39) | *Intervention*  All groups had access to the Healthy Me Programme (HMP), which allowed to consult health coaches and self-monitor their PA and dietary intake. In addition, VCG and IPG received educational lessons on PA, diet and portion control in video-conferencing or in-person lessons respectively.  *eHealth features*  Computers to allow video-conferencing for the VCG group. | | *Primary outcomes*   - Proportion of participants who reduced ≥2 kg.   *Secondary outcomes*   - Weight - Attendance or usage of intervention | *Primary outcomes*  At 12 months, proportion of participants who lost ≥2 kg were 17/35 (48.6%) in VCG, 14/44 (31.8%) in IPG, and 15/47 (31.9%) in CG. There were no between-group differences in proportion of participants (*P*=.22).  *Secondary outcomes*  At 12 months, percentage of weight gain in VCG was 1.59% (95% CI -.45 to 3.62), and percentage weight loss for IPG and CG were -.41% (95%CI -2.2 to 1.39) and -.11% (95%CI -1.51 to 1.29) respectively. There were no between-group differences in percentage weight loss (*P*=.24). 58% of VCG and 45% of IPG attended at least one training session. |
| Das et al (2017), United States ^88^. | | | | | |
| *Study Design* | *Participant Information* | | *Intervention description* | *Outcome measures* | *Results* |
| *Design*  Pre-post study  *Group Info*   - n=45 (Community + in person) - n=309 (Community + videoconference) - n=132 (Worksite + in person) - n=158 (Worksite + video conference)     *Duration*  11 weeks    *Measurement points*  Baseline, 11 weeks    *Attrition rate*  28.4% (183/644) | *N* = 644  *Sample*  Healthy Adults  *Mean age*  Age provided in range:  *n*=43 (18-29.9 years)  *n*=77 (30-39.9 years)  *n*=165 (40-49.9 years)  *n*=201(50-59.9 years)  *n*=158(>60 years)    *Gender distribution*   - 87.4% female (563) - 12.6% male (81) | | *Intervention*  iDiet weight loss program, weekly 1-hour group meetings (in person or video conference, in community or worksite). Nutrition and weight management education, support and discussion.  *eHealth features*  Website message board, log weight in website, online video conferencing. | *Primary outcomes*   - Weight | *Primary outcomes*  Mean Δ of percentage weight loss was 7.4(+-3.6) compared to baseline.  Age category, program class type  (worksite vs. community group), and year and month of enrolment were significantly associated with percentage weight loss. 2017  Program delivery method (videoconference vs. in-person) and payment structure (for worksite, different payment types) were not significantly associated with percentage weight loss.  No significant  interaction between program delivery and class type |
| Dennison et al (2014), United Kingdom ^75^. | | | | | |
| *Study Design* | *Participant Information* | *Intervention description* | | *Outcome measures* | *Results* |
| *Design*  3-group RCT  *Group Info*   - Web only group (W),  n = 264 - Web + coach group (WC), n = 247 - Control group (CG), n = 275   *Duration*  2 months    *Measurement points*  Baseline, 2 months    *Attrition rate*   - W = 84.8% - WC = 78.5% - CG = 41.1% | *N* = 786  *Sample*  Healthy adults  *Mean age*   - W = 43.3 (12.5) - WC - = 44.4 (12.6) - CG = 44.2 (13)     *Gender distribution*   - W = 82.5% female (217) - WC = 78.9% female (195) - CG = 78.5% female (216) | *Intervention*  Users select a diet plan and set personal target to achieve their health goals. For the WC group, additional coaching calls from researchers were conducted to encourage them to continue using the programme.  *eHealth features*  Web-based programme that allowed self-monitoring, goal-setting, and provided reminders and cognitive and behavioural strategies to users. | | *Primary outcomes*   - Usage of the POWeR programme   *Secondary outcomes*   - Weight | *Primary outcomes*  In general, usage was low with only 17.8% in the W group and 25.9% in the WC group. WC group used the intervention longer than the W group and were 1.61 more likely to complete more than three sessions.  *Secondary outcomes*  Compared to controls, W [(mean Δ = -2.01 kg (3.45)] and WC group [mean Δ = -2.27kg (3.41)] had greater weight loss. There were no differences in weight change between W and WC group (mean difference = 0.27kg, *P*=.68). |
| Duan et al (2018), China (Hong Kong) ^34^. | | | | | |
| *Study Design* | *Participant Information* | *Intervention description* | | *Outcome measures* | *Results* |
| *Design*  2-group RCT  *Group Info*   - Intervention group (IG),  n = 60 - Control group (CG), n = 54   *Duration*  2 months    *Measurement points*  Baseline, 2 months    *Attrition rate*  IG = 26.7%  CG = 27.8% | *N* = 114  *Sample*  Patients with coronary heart disease  *Mean age*   - IG = 45.8 (14.68) - CG = 51.57 (11.57)     *Gender distribution*   - IG = 54.4% female - CG = 51.9% female | *Intervention*  It includes a web-based programme. Users set health goals and self-monitor their PA levels and FVC. Feedback on their health goals and progress were provided. Users also develop their own action plan to help them achieve their health goals.    *eHealth features*  Web-based programme. | | *Primary outcomes*   - PA levels - Fruit and Vegetable Consumption (FVC) - BMI | *Primary outcomes*  Over the intervention period, IG had greater PA levels and FVC as compared to CG (eta-squared effect ranged from .06 to .43). |
| Duncan et al (2014), Australia ^35^. | | | | | |
| *Study Design* | *Participant Information* | *Intervention description* | | *Outcome measures* | *Results* |
| *Design*  2-group RCT    *Group Info*   - IT-based intervention, *n* = 205 - Print-based intervention, *n* = 96     *Duration*  9 months  *Measurement points*  Baseline, 3 months, 9 months    *Attrition rate*  At 3 months:   - IT-based intervention, 52% attrition from baseline - Print-based intervention, 31% attrition from baseline   At 9 months:   - IT-based intervention, 3% attrition from 3 months - Print-based intervention, 13% attrition from 3 months | *N* = 301    *Sample*  Healthy male participants  *Mean age*   - IT-based intervention = 44.17 (.41) - Print-based intervention = 43.84 (.59)     *Gender distribution*  100% male | *Intervention*  Both groups received the same intervention materials. All participants self-monitored their PA and dietary patterns, set personal health goals, take part in challenges related to healthy eating and exercise, and were provided with educational materials. In addition, the IT group received personalised feedback on their progress, and had the ability to interact with other users on the web app. The print group received hard-copy booklets which included general health information, and log books for diet and PA levels.  *eHealth features*  The IT-based intervention involved an app- and mobile-based app called the ManUp intervention. | | *Primary outcomes*   - Dietary behaviour - Physical Activity (PA) level - Health literacy - Usage of IT-based intervention | *Primary outcomes*  There were no between-group differences in dietary behaviour, PA levels, and health literacy at 9 months (all *P*>.05).  For the IT-group, usage of the IT-platform increased with time. The average number of log-ins to the IT platform at 3 months and 9 months were 6.99 (.86) and 9.22 (1.47) respectively. The average number of self-monitoring entries at 3 months and 9 months were 16.69 (2.38) and 22.51 (3.79) respectively. |
| Elbert et al (2016), Netherlands ^91^. | | | | | |
| *Study Design* | *Participant Information* | | *Intervention description* | *Outcome measures* | *Results* |
| *Design*  3-group RCT  *Group Info*   - Text-based, *n* = 114 - Audio-based, *n*= 113 - Control group, *n* = 115   *Duration*  6 months    *Measurement points*  Baseline, 6 months    *Attrition rate*  55% | *N* = 342    *Sample*  Healthy adults    *Mean age*   - 41.4 (14.6)     *Gender distribution*   - 73.3% female | | *Intervention*  Monthly text-based or audio-based tailored health information and feedback (via mobile phone app) aimed at stimulating fruit and vegetable intake.    *eHealth features*  Mobile phone app | *Primary outcomes*   - Dietary intake (fruit consumption) | *Primary outcomes*  Audio-based group, but not text-based group, increased fruit consumption (increase by an average of 3 pieces of fruit a week compared to baseline)  Main effect on fruit consumption remained  significant (F_2,323_=3.18, *P*=.04, partial η^2^=0.02); IG(text-based) mean 13.5(SE 0.52), IG(audio-based) mean 15.3(SE 0.52), CG mean 14.3(SE 0.51).  No significant main effect of condition with regard to vegetable consumption. |
| Eyles et al (2017), New Zealand ^36^. | | | | | |
| *Study Design* | *Participant Information* | *Intervention description* | | *Outcome measures* | *Results* |
| *Design*  2-group RCT    *Group Info*   - IG, *n* = 33 - CG, *n* = 33     *Duration*  1 month  *Measurement points*  Baseline, 1.5 months  *Attrition rate*  0% | *N* = 66  *Sample*  Patients with cardiovascular disease    *Mean age*   - IG = 64 (7.0) - CG = 65 (8.0)   *Gender distribution*   - IG = 24% female - CG = 90% female | *Intervention*  IG used the app when they shop for food. When using the app, they receive nutritional information of food, in particular, salt content of food. They were sent weekly text messages to remind and encourage them to use the app. CG received usual care.  *eHealth features*  Mobile application, SaltSwitch, allows users to scan barcode of packaged food and receive nutritional information of food. The app is able to provide healthier food choices with lower salt content. | | *Primary outcomes*   - Salt content of food purchases   *Secondary outcomes*   - Saturated fat content of food purchases - Energy content of food purchases - Systolic BP - Urinary sodium | *Primary outcomes*  IG purchased food items with lower salt content than CG (mean = -.3, 95% CI -.58 to -.03, *P*=.03).  *Secondary outcomes*  There were no differences between groups on the saturated fat, and energy content of purchased food. There were no group differences in systolic BP (mean = -1.7, 95% CI -7.4 to 3.9, *P*=.54) and urinary sodium (mean = -10.6, 95% CI -278 to 256, *P*=.94). |
| Fernandes et al (2016), Brazil ^71^. | | | | | |
| *Study Design* | *Participant Information* | *Intervention description* | | *Outcome measures* | *Results* |
| *Design*  2-group RCT  *Group Info*   - IG, *n* = 104 - CG, *n* = 106, received usual care   *Duration*  6 months  *Measurement points*  Baseline, 3 months, 6 months  *Attrition rate*  20% | *N* = 110    *Sample*  Diabetes patients  *Mean age*   - IG = 62.9 (10.2) - CG = 62.9 (10.3)   *Gender distribution*   - IG = 73.1% female - CG = 64.1% female | *Intervention*  Telephone intervention aimed to encourage physical exercise and healthy diet. There were 3 types of telephone interventions: 1) building rapport between nurse and participants, 2) provide support and guidance on physical activity and following a diet plan, 3) encouraging participants to maintain health behaviours.  *eHealth features*  Telephone calls | | *Primary outcomes*   - Diabetes self-care questionnaire | *Primary outcomes*  At 6 months, IG has a progressive increase in diabetes self-care scores from baseline compared to CG [mean = 1.97 (0.96) in IG vs mean = 0.57 (1.53) in CG, 95% CI 1.03 to 1.78], thereby indicating that they were better in diabetes self-management. |
| Fukuoka et al (2015), United States ^50^. | | | | | |
| *Study Design* | *Participant Information* | *Intervention description* | | *Outcome measures* | *Results* |
| *Design*  2-group pilot RCT    *Group Info*   - IG, *n* = 30 - CG, *n* = 31   *Duration*  5 months  *Measurement points*  Baseline, 3 months, 5 months  *Attrition rate*  0% | *N* = 61    *Sample*  Middle-aged adults at risk of diabetes  *Mean age*   - IG = 57.1 (9.1) - CG = 53.4 (8.7)   *Gender distribution*   - IG = 76.7% female - CG = 77.4% female | *Intervention*  All participants received standard care and a pedometer to track PA levels. IG used the app to self-monitor weight, PA level, and caloric intake. IG set health goals and the app provided health recommendations such as reducing consumption of sugars and fat.    *eHealth features*  mDPP (mobile phone-based diabetes prevention program) is a mobile app that allows users to self-monitor health data, PA levels, and caloric intake. It included a reminder system to remind IG to enter data into the app. A pedometer was also provided to record PA levels. | | *Primary outcomes*   - Weight - BMI   *Secondary outcomes*   - WC - BP level - Lipid profile - Glucose level - PA levels - Caloric and fat intake (self-reported using Block Food Frequency Questionnaire) - 7-day physical activity recall - Self-efficacy for PA - Social support and exercise survey - Depressive symptoms measured by Center Epidemiologic Studies Depression Scale (CES-D) | *Primary outcomes*  At 5 months, IG had a significant reduction in weight [-6.2 (5.9) kg] and BMI [-2.2 (2.2)] compared to an increase in weight [0.3 (2.7)kg] and BMI [0.1 (1.0)] for CG (all *P*<.001).  *Secondary outcomes*  At 5 months, IG had smaller hip circumference [108.8 (10.9)] than CG [115.9 (14.4)], *P*=.008. Systolic BP level was lower in IG [121.1 (11.1)] than CG [129.5 (12.1)], *P*=.03. Diastolic BP was lower in IG [73.7 (7.0)] than CG [80.2 (8.1)], *P*=.003. IG had greater increase in step count [2551 (4712)] compared to a decrease in step count for CG [-734 (3308)], *P*<.001. IG reported increase in PA levels (*P*=.03). IG had greater reduction than CG in saturated fat [16.2 (8.3) vs 22.0 (9.8), *P*=.007] and sugar-sweetened drinks [7.3 (12.3) vs 19.3 (36.4), *P*=.02].  No differences in other secondary outcomes between groups. |
| Gilson et al (2017), Australia ^37^. | | | | | |
| *Study Design* | *Participant Information* | *Intervention description* | | *Outcome measures* | *Results* |
| *Design*  2-group pre-post study    *Group Info*  All participants took part in intervention  *Duration*  5 months  *Measurement points*  Baseline, 5 months, 7 months  *Attrition rate*  27% | *N* = 19    *Sample*  Healthy middle-aged adults (truck drivers)  *Mean age*  47.5 (9.8)  *Gender distribution*  100% male | *Intervention*  The intervention program included an incentive point-based system whereby IG could accumulate points and redeem cash prizes for achieving health goals (step counts and healthier food choices). Users used the app to enter daily dietary choices either by manual entry, or scanning a barcode. They were provided with monthly personalised feedback, and were encouraged to continue to use the app throughout the program. Wearables were provided to track step count.  *eHealth features*  A mobile app called UP, and a wearable activity tracker called Jawbone UP. | | *Primary outcomes*   - Dietary intake (self-reported) - PA level | *Primary outcomes*  Self-reported consumption of fruit and vegetable increased at the end of program (4 servings/day at baseline to 5 servings/day at 5 months, *P*=.02), but returned to baseline at follow-up. There were non-significant reductions in self-reported consumption of saturated fats and processed sugar (all *P>*.05). There were non-significant increases in PA levels at end of program and follow-up (*P*>.05). |
| Gomez-Marcos et al (2018), Spain ^76^. | | | | | |
| *Study Design* | *Participant Information* | *Intervention description* | | *Outcome measures* | *Results* |
| *Design*  2-group RCT    *Group Info*   - Counselling + app group, *n* = 415 - Counselling only group, *n* = 418     *Duration*  3 months  *Measurement points*  Baseline, 3 months, 12 months  *Attrition rate*   - Counselling + app group = 15.18% - Counselling only group = 13.16% | *N* = 833  *Sample*  Healthy older adults  *Mean age*   - Counselling + app group = 51.44 (12.22) - Counselling only group = 52.33 (12)     *Gender distribution*   - Counselling + app group = 60% female - Counselling only group = 64.1% female | *Intervention*  All participants received 30-min counselling on the PA and benefits of having a Mediterranean diet. For the IG, they received an additional mHealth application (smartphones) for 3 months, designed to promote adoption of Mediterranean diet and increase PA.  *eHealth features*  Record daily food intake, and any PA performed that was not detected by the smartphone’s pedometer. Pedometer in-built to the smartphone to record step counts and PA. App is able conduct daily analysis of PA and food intake, and can generate a plan of recommendations for the following days with the aim to improve diet and increase PA. | | *Primary outcomes*   - BMI - Waist circumference (WC) - Clinica Universidad de Nacarra – body adiposity estimator (CUN-BAE). | *Primary outcomes*  At 3 months, there were significant reduction of 0.7% in BMI amongst women in the counselling+app group (95% CI: −0.27 to −0.04). At 12 months, BMI increased by 0.7% in the counselling only (95% CI: 0.01−0.29).  There was a decrease of WC by 0.7% (95% CI: −1.888 to −0.160) and a decrease of CUN-BAE by 0.8% (95% CI: −0.923 to −0.075) amongst women in the counselling+app group. BMI increased by 1.1% (95% CI: 0.033 to 0.486) amongst men in the counselling+app group, and by 0.9% (95% CI: 0.008 to 0.282) in the counselling only group. |
| Haas et al (2019), Switzerland ^77^. | | | | | |
| *Study Design* | *Participant Information* | *Intervention description* | | *Outcome measures* | *Results* |
| *Design*  Pre-post pilot study    *Group Info*  All participants took part    *Duration*  12 months  *Measurement points*  Baseline, 3 months, 12 months  *Attrition rate*  16% | *N* = 43  *Sample*  Patients with obesity  *Mean age*  40.6 (12.4)    *Gender distribution*  84% female (36) | *Intervention*  Participants used the Oviva app on their mobile phones which facilitated their communication with a dietician. Users self-monitored their diet, PA levels, had access to nutritional content, set health goals, and received feedback and support from dietician.  *eHealth features*  Mobile app called Oviva | | *Primary outcomes*   - Weight   *Secondary outcomes*   - hbA1c level - Fasting glucose level - Triglycerides level - Insulin level - HDL-cholesterol - Blood pressure - BMI - Waist circumference - Body fat - PA level - Dietary assessment - Quality of life | *Primary outcomes*  At 12 months, the median weight loss was -4.9kg (*P*<.001) compared with baseline.  *Secondary outcomes*  At 12 months, participants significantly reduced BMI, waist circumference, body fat, and blood pressure (all *P*<.001) compared with baseline. There were no changes to their hbA1c, fasting blood glucose, insulin, triglycerides, HDL-cholesterol levels, PA levels and quality of life (all *P*>.05). Participants improved their diet by consuming more fruits and vegetables, and consuming less alcohol, sweet, and fats (*P*<.001) at 12 months. |
| Hageman (2017), United States ^92^. | | | | | |
| *Study Design* | *Participant Information* | | *Intervention description* | *Outcome measures* | *Results* |
| *Design*  3 group RCT    *Group Info*   - Web-based only (WO), n = 101 - Web-based discussion (WD), n = 100 - Web-based email (WE), n = 100   *Duration*  Intervention was in 3 phases:  Baseline to 6 months, 6 to 18 months, 18 to 30 months    *Measurement points*  Baseline, 6, 18, 30 months    *Attrition rate*  25% | *N* = 301  *Sample*  Women from underserved rural communities  *Mean age*   - 53.9(6.9) years     *Gender distribution*   - 100% female | | *Intervention*  Web-based intervention, with peer-led discussion, or professional email counselling. Behaviour change lifestyle plan on the website, eating and activity recommendations  *eHealth features*  Website with weekly posted content, peer-led discussion blog, and email. Pedometer to record daily steps and physical activity. | *Primary outcomes*   - Body weight (kg) - Waist circumference (cm) - Kcal intake daily - Weekly minutes moderate or greater intensity activity - Blood pressure level - Cholesterol level - Triglycerides level - Fasting glucose level   *Secondary outcomes*   - Calorie intake - Physical activity level | *Primary outcomes*  All groups had a decrease in weight and waist circumference, but inferential statistics and significance values were not reported. There were no group differences in mean change for weight and waist circumference.  Compared to baseline, decrease in systolic and diastolic BP after 6 months in WE, and a decrease in systolic BP in WO.  *Secondary outcomes*  No significant pairwise comparisons for calorie intake, weekly minutes of moderate or greater intensity activity, blood pressure, or lipids. |
| Haggerty et al (2017), United States ^51^. | | | | | |
| *Study Design* | *Participant Information* | *Intervention description* | | *Outcome measures* | *Results* |
| *Design*  3-group RCT  *Group Info*   - Telemedicine, *n* = 14 - Text4Diet, *n* = 13 - Usual care, *n* = 15     *Duration*  6 months  *Measurement points*  Baseline, 6 months  *Attrition rate*  22% | *N* = 41  *Sample*  Endometrial cancer survivors with obesity  *Mean age*  59.7 (8.7) for all groups    *Gender distribution*   - 100% female in all groups | *Intervention*  Telemedicine group received 15-20 min counselling sessions via telephone calls about weight loss management. Text4Diet group received 3-5 personalised text messages about weight loss management daily. The usual care group received handouts including healthy eating, exercise and weight management.    *eHealth features*  Telephone calls and text messages. | | *Primary outcomes*   - Weight - WC - PA level - Physical Health SF-12 | *Primary outcomes*  At 6 months, no changes in weight and WC for all groups compared to baseline (*P*>.05). No group differences observed in weight and WC. The usual care group had the highest increase in total PA level (*P*=.01) compared to the other groups. Compared to baseline, all groups increased physical health SF-12 scores at 6 months (*P*=.04). |
| Hales et al (2016), United States ^52^. | | | | | |
| *Study Design* | *Participant Information* | *Intervention description* | | *Outcome measures* | *Results* |
| *Design*  2-group RCT  *Group Info*   - IG, *n* = 26 - Comparison group, *n* = 25     *Duration*  3 months  *Measurement points*  Baseline, 3 months  *Attrition rate*  18% | *N* = 51  *Sample*  Overweight middle-aged adults  *Mean age*   - IG = 48.4 (11.9) - Comparison group = 43.9 (12.7)     *Gender distribution*   - IG = 85% female - Comparison group = 80% female | *Intervention*  IG used the Social POD app, while the comparison group used a commercially available app. Both types of apps allowed users to record dietary intake and PA levels, included a calorie and PA database, received health information, and were reminded to track and record their weight. In addition, the Social POD app included an additional element of social networks, notifications, and point-based system. IG users are able to send motivational and supportive messages to each other to encourage them to continue to use the app. IG were able to set goals, and earn points for goal achievement.  *eHealth features*  Mobile app called Social POD app, which was developed by the researchers, was used in IG. Commercially available tracking app (Calorie Counter app) were also used in the comparison group. | | *Primary outcomes*   - Weight     *Secondary outcomes*   - BMI - Caloric intake and expenditure - Social support for health behaviours (diet and exercise) - Self-efficacy for weight-loss behaviours - Expectations of treatment | *Primary outcomes*  At 3 months, IG had greater weight loss than comparison group from baseline (-5.3kg, 95% CI: -7.5 to -3.0 in IG vs -2.2kg, 95% CI: -⅗ to -1.0 in comparison group), *P*=.02.  *Secondary outcomes*  IG also had greater reduction in BMI compared to comparison group (-1.91, 95% CI: -2.6 to -1.2 in IG vs -.90, 95% CI: -1.4 to -.45 in comparison group), *P*=.02. There was a greater reduction in positive expectations of the treatment in comparison group compared to IG (*P*=.04). There were no between-group differences in other secondary outcomes (all *P*>.05). |
| Hansel et al (2017), France ^93^. | | | | | |
| *Study Design* | *Participant Information* | | *Intervention description* | *Outcome measures* | *Results* |
| *Design*  2-group RCT  *Group Info*   - IG,  *n* = 60 - CG, *n* = 60     *Duration*  4 months    *Measurement points*  Baseline, 4 months    *Attrition rate*  10.8% | *N* = 120  *Sample*  Participants with abdominal obesity and type 2 diabetes with haemoglobin  A1c (HbA1c) >5.6% and <8.5%    *Mean age*   - IG = 57.6 (8.1) - CG = 55.5 (10.3)     *Gender distribution*   - IG = 66.7% female - CG = 66.7% female | | *Intervention*  ANODE e-coaching program has 4 modules and there are: 1) diet and physical activity self-monitoring module, 2) nutritional  assessment, 3) balanced diet menu generator, and 4) physical  activity education and prescription program  *eHealth features*  Web-based nutritional support tool, automated digital scale, pedometer | *Primary outcomes*   - Body weight - BMI - Waist circumference - BP - VO2 - HbA1c - DQI-I score (International Diet Quality Index) - IPAQ (International Physical Activity Questionnaire)   *Secondary outcomes*   - IPAQ (International Physical Activity Questionnaire) - Fasting blood glucose, - Total cholesterol, LDL-C, HDL-C, - Triglycerides, - Serum Glutamic Pyruvic - Transaminase, - Serum Glutamic Oxaloacetic - Transaminase, Gammaglutamyl-transferase, - hs-CRP, - Uric acid, - Creatinine | *Primary outcomes*  At 16 weeks, IG had significantly greater reduction in body weight (kg) [IG=-2.3(3.0); CG=0.2(2.5); *P*=.01], waist circumference (cm) [IG=-0.9(4.7);CG=0.8(3.6); *P*=.01], and HbA1c (%) [IG=-0.30(0.94); CG=0.21(0.70); *P*<.001] compared to CG.  At 4 months, IG had significantly greater improvements in DQI-score [+4.55 (5.91)], compared to CG [-1.68 (5.18)], *P*<.001.  No between-group differences in VO2 max, and physical activity (all *P*>.05).  *Secondary outcomes*  No significant differences between groups in secondary outcomes (all *P*>.05) |
| Hartman et al (2016). United States ^53^. | | | | | |
| *Study Design* | *Participant Information* | *Intervention description* | | *Outcome measures* | *Results* |
| *Design*  2-group RCT  *Group Info*   - Intervention, *n* = 36 - CG, *n* = 18   *Duration*  6 months  *Measurement points*  Baseline, 6 months  *Attrition rate*   - Intervention = 8% - CG = 5% | *N* = 54  *Sample*  Women at-risk of breast cancer  *Mean age*   - Intervention = 59.4 (5.6) - CG = 59.8 (5.9)   *Gender distribution*  100% female in both groups | *Intervention*  IG input weight, goal weight, and goal rate of weight loss, and the app provides personalised daily calorie targets to help participants achieve weight goals. They also entered daily food intake and activity levels via a wearable device, Fitbit. The app generates daily nutrition and PA reports. Over 6 months, IG also received 12 phone calls (30 mins each) from health coaches to provide in-depth coaching and support for achieving weight loss goals.  CG received usual care and 2 short phone calls from clinicians, but without in-depth health coaching.  *eHealth features*  Mobile application, MyFitnessPal. | | *Primary outcomes*   - Weight - PA level | *Primary outcomes*  At 6 months, IG had greater reduction in weight (mean = 4.4 in IG vs mean = 0.8 in CG, *P*=.004). IG and CG increased PA level by 15.01 (14.2) and 10.9 (10.1) respectively, but there were no group differences (*P*>.05). |
| Holmen et al (2014), Norway ^78^. | | | | | |
| *Study Design* | *Participant Information* | *Intervention description* | | *Outcome measures* | *Results* |
| *Design*  3-parallel group RCT  *Group Info*   - Few Touch Application (FTA) group, *n* = 51 - Few Touch Application with Health Counselling (FTA-HC), *n* = 50 - CG, *n* = 50   *Duration*  12 months  *Measurement points*  Baseline, 4 months, 12 months  *Attrition rate*  21% | *N* = 151  *Sample*  Patients with diabetes mellitus (Type 2)  *Mean age*   - FTA group = 58.6 (11.8) - FTA-HC = 57.4 (12.1) - CG = 55.9 (12.2)   *Gender distribution*   - FTA group = 33% female - FTA-HC = 50% female - CG = 40% female | *Intervention*  FTA used the mHealth app to self-manage their diabetes by entering daily dietary and PA levels. They recorded their BP and readings will be sent to the mHealth app via Bluetooth. In addition to the mHealth app, the FTA-HC group received health counselling sessions via phone calls for 4 months. All groups received usual care.  *eHealth features*  Few Touch Application self-management system on smart phones. It includes a diabetes diary application in which they can record and track blood glucose levels, food habits, PA levels, and personal health goals. It provides health reports, and motivational feedback. | | *Primary outcomes*   - HbA1c level   *Secondary outcomes*   - Weight - Health Education Impact Questionnaire (heiQ) score - Dietary habits - PA levels - Quality of life measured by Short-Form 36v2 Health Survey - Depressive symptoms measured by CES-D | *Primary outcomes*  There were no within-group changes of HbA1c levels for all groups [FTA: 65 (61, 70) at baseline, 62 (58, 66) at 1 year, 95% CI: -3.4 (-7.4 to 0.6), FTA-HC: 65 (61, 69) at baseline, 63 (58, 68) at 1 year, 95% CI: -⅙ (-6.3, 3.1), CG: 68 (64, 72) at baseline, 66 (62, 71) at 1 year, 95% CI: -1/7 (-5.4 to 2.0)].  *Secondary outcomes*  There were no within-group changes for all secondary outcomes in all groups, with the exception of skill and technique acquisition domain in the heiQ score of the FTA-HC group [2.89 (2.75, 3.02) at baseline, 3.08 (2.96, 3.21), 95% CI: 0.19 (.05 to .33), *P*<.05]. |
| Huber et al (2015), United States ^54^. | | | | | |
| *Study Design* | *Participant Information* | *Intervention description* | | *Outcome measures* | *Results* |
| *Design*  2-group RCT  *Group Info*   - Intervention, *n* = 45 - CG, *n* = 45     *Duration*  3 months    *Measurement points*  Baseline, 6, 12, 18, 24 weeks    *Attrition rate*  15.6% | *N* = 90    *Sample*  Patients with obesity  *Mean age*   - Intervention = 48.3 (12.3) - CG = 47.4 (14.1)   *Gender distribution*   - Intervention = 84% female (n=38) - CG = 64% female (n=29) | *Intervention*  IG received a portion control plate and were taught how to use it alone with telecoaching. Telecoaching were conducted every two weeks, lasting about 20 mins per session. The sessions involved motivational interviewing aimed to improve patients’ lifestyle habits.  *eHealth features*  Telephone calls | | *Primary outcomes*   - Weight - BMI - Waist circumference - Waist-to-hip ratio   *Secondaary outcomes*   - Eating behaviours - PA level - Weight Efficacy Life-Style Questionnaire (WEL) | *Primary outcomes*   - At 3 months, there were greater reduction in BMI (estimated treatment effect -0.4kg/m^2^, *P*=.038) and waist-to-hip ratio (estimated treatment effect -.02, *P*=.037) in IG compared to CG. These changes were not significantly different between groups at 6 months.   *Secondary outcomes*   - There were no differences in changes of secondary outcomes between groups. |
| Järvelä-Reijonen et al (2018), Finland ^79^. | | | | | |
| *Study Design* | *Participant Information* | *Intervention description* | | *Outcome measures* | *Results* |
| *Design*  3-parallel group RCT  *Group Info*   - Face-to-face group (FTF), *n* = 70 - Mobile app group (MA), *n* = 78 - CG, *n* = 71   *Duration*  2 months    *Measurement points*  Baseline, 10 weeks, 36 weeks  *Attrition rate*  At 36 weeks:   - FTF group = 11% - MA group = 4% - CG = 4% | *N* = 219  *Sample*  Adults with psychological distress and obesity  *Mean age*   - FTF group = 50.3 (7.2) - MA group = 49.1 (7.7) - CG = 49.2 (7.4)     *Gender distribution*   - FTF group = 87% female (61) - MA group = 84.6% female (66) - CG = 81.7% female (58) | *Intervention*  Both FTF and MA groups took part in the intervention that involved acceptance and commitment therapy (ACT) in changing and improving health behaviours such as mindfulness, and healthy dietary habits. Education about nutrition was not part of the intervention. FTF attended sessions on-site, while MA accessed modules via a mobile app. CG received no intervention.  *eHealth features*  MA group used a mobile app (Oiva app). | | *Primary outcomes*   - Intuitive eating scale (IES) score - Three-factor eating questionnaire (TFEQ) score - Health and Taste attitude scale (HTAS) score - Eating competence - Regulation of eating behaviour (REBS) score - Food and nutrient intake (Index of Diet Quality, IDQ score) - Alcohol consumption - 48h dietary recall - Perceived stress (PSS) score | *Primary outcomes*  Group x time interaction were found to be significant for the subcomponent “eating for physical rather than emotional reasons” on the IES, uncontrolled eating on the TFEQ, using food as a reward on the HTAS, food acceptance, integrated and identified regulation on the REBS, such that FTF group did better than the other groups. No between group differences in other measures were found.  Within-group comparisons showed mixed results in the significant improvements of dietary scores amongst groups. |
| Kanera et al (2017), Netherlands ^80^. | | | | | |
| *Study Design* | *Participant Information* | *Intervention description* | | *Outcome measures* | *Results* |
| *Design*  2-group RCT  *Group Info*   - Intervention, *n* = 231 - CG, *n* = 231     *Duration*  6 months  *Measurement points*  Baseline, 6 months, 12 months  *Attrition rate*  17.5% | *N* = 462    *Sample*  Cancer survivors  *Mean age*   - Intervention = 55.6 (11.5) - CG = 56.2 (11.3)   *Gender distribution*   - Intervention = 79.2% female - CG = 80.5% female | *Intervention*  IG received personalised advice on different health modules, of which one of them was on diet and nutrition. IG were encouraged to increase healthy eating behaviours such as consuming more dietary fibre, whole grains, and fish. They were also encouraged to set dietary goals.  *eHealth features*  Web-based intervention Kanker Nazorg Wijzer (Cancer Aftercare Guide, KNW). | | *Primary outcomes*   - Daily food intake measured by 8-item Dutch Standard Questionnaire on Food Consumption - The frequency and serving sizes of vegetable consumption | *Primary outcomes*  IG increased vegetable intake at 6 months (*B*= 11.799, *P*=.001), but intervention effects were not maintained at 12 months (*B* = 5.860, *P*=.121). No change in vegetable intake in CG. |
| Kempf et al (2017), Germany ^81^. | | | | | |
| *Study Design* | *Participant Information* | *Intervention description* | | *Outcome measures* | *Results* |
| *Design*  2-group RCT  *Group Info*   - Intervention, *n* = 102 - CG, *n* = 100   *Duration*  3 months    *Measurement points*  Baseline, 12, 26, 52 weeks  *Attrition rate*  17% | *N* = 202  *Sample*  Patients with type 2 diabetes  *Mean age*   - Intervention = 59 (9) - CG = 60 (8)   *Gender distribution*   - Intervention = 79.2% female - CG = 80.5% female | *Intervention*  All participants received a weighing scale and step count. IG received telemedical coaching that aimed to provide motivation, were provided with formula diet regimen, and self-monitored hbA1c levels. CG received usual care.    *eHealth features*  Telephone calls | | *Primary outcomes*   - HbA1c levels - FBG level   *Secondary outcomes*   - Weight - Blood pressure - Cholesterol level - Triglycerides level - CVD risk - Quality of life - Diet behaviour - Antidiabetes medication | *Primary outcomes*  At 12 weeks, there were significant reduction in hbA1c levels (mean change = -1.1, *P*<.0001) and FBG levels (mean change = -21, *P*<.05) in IG compared to baseline. Significant reductions were maintained at 52 weeks. No changes in hbA1c levels (mean change = -.2, *P*>.05) and FBG levels (mean change = -5, *P*>.05) in CG.  *Secondary outcomes*  At 12 weeks, significant reduction in systolic BP (mean change = -6, *P*<.05) and triglycerides level (mean change = -24, *P*<.01) in IG compared to baseline. There were improvements in quality of life, eating behaviours, and MES medication in IG at 12 weeks compared to baseline. No changes observed for other outcomes. No changes found in CG for other outcomes. |
| Khanna et al (2014), United States ^55^. | | | | | |
| *Study Design* | *Participant Information* | *Intervention description* | | *Outcome measures* | *Results* |
| *Design*  2-group RCT  *Group Info*   - Intervention, *n* = 38 - CG, *n* = 37   *Duration*  3 months    *Measurement points*  Baseline, 3 months  *Attrition rate*  34.7% | *N* = 75  *Sample*  Patients with type 2 diabetes  *Mean age*   - Intervention = 51 (12) - CG = 53 (12)   *Gender distribution*   - Intervention = 50% female (19) - CG = 32% female (12) | *Intervention*  IG were educated on the importance of lowering high-glycemic index foods and common foods which have a high-glycemic index. The automated nutrition support (ATNS) system allows users to self-assess and feedback on their food intake by responding to the telephone key pad. IG received 2 phone calls peer week for 3 months. CG received usual care.    *eHealth features*  Telephone calls and telephone key pad for users to provide feedback. | | *Primary outcomes*   - HbA1c levels   *Secondary outcomes*   - BMI - Waist circumference - Cholesterol level - Blood pressure level - Triglycerides level | *Primary outcomes*  At 3 months, there were no significant differences between groups in hbA1c levels (*P*=.43). No significant changes were observed within groups as well.  *Secondary outcomes*  There were no significant changes within and between groups in all secondary outcomes at 3 months when compared to baseline. |
| Koot et al (2019), Singapore ^87^. | | | | | |
| *Study Design* | *Participant Information* | | *Intervention description* | *Outcome measures* | *Results* |
| *Design*  Single-arm feasibility study  *Group Info*   - All participants took part in intervention   *Duration*  6 months    *Measurement points*  Baseline, 6months    *Attrition rate*  17% | *N* = 100    *Sample*  Patients with type 2 diabetes, and HbA1c ≥7.5%    *Mean age*   - 53.5 (9.6)   *Gender distribution*   - 50% female | | *Intervention*  Glyco leap program was a comprehensive T2DM educational curriculum  delivered through online lessons. The Glyco mobile  app had health coaching component. Users log and monitor blood glucose levels, weight, meals, and physical activity, captured via pedometer. Health coaches provide  personalized feedback to participants on their progress and to  present opportunities for improvement.  *eHealth features*  Mobile app to log daily meals and physical activity tracking | *Primary outcomes*   - HbA1c - Weight - Dietary habits - Physical activity level | *Primary outcomes*  At 6 months, there were significant reductions in HbA1c [-1.1(-1.4 to -0.7); *P*<.001] and weight [-2.0(-2.8 to -1.2); *P*<.001] compared to baseline. There were improvements in their dietary habits post-intervention, as they increased fruit and vegetable consumption by 2.4 (95% CI: 1.6 to 3.1) servings/week (*P*<.001), and decreased high fat food consumption by -0.7 (95%CI: -1.1 to -0.2) servings/week (*P*=.003). There were no changes to their physical activity level (*P*=.14). |
| Lim et al (2016), South Korea ^38^. | | | | | |
| *Study Design* | *Participant Information* | *Intervention description* | | *Outcome measures* | *Results* |
| *Design*  2-group RCT  *Group Info*   - Intervention, *n* = 50 - CG, *n* = 50   *Duration*  6 months    *Measurement points*  Baseline, 3 months, 6 months  *Attrition rate*  15% | *N* = 100  *Sample*  Patients with HbA1c level <7% without hypoglycaemia  *Mean age*   - Intervention = 64.3 (5.2) - CG = 65.8 (4.7)   *Gender distribution*   - Intervention = 20% female (10) - CG = 30% female (15) | *Intervention*  IG used a public switched telephone network that is connected to a glucometer to measure their blood glucose level. They used a wearable technology to record their PA levels. They entered their daily dietary intake on the website and the data input were automatically converted to total caloric intake and amount of macronutrient components. CG received usual care and self-measured their blood glucose level    *eHealth features*  Ubiquitous health system using telephone line, web system, glucometer and wearable technology. | | *Primary outcomes*   - HbA1c levels   *Secondary outcomes*   - BMI - Fat mass - Blood pressure level - Cholesterol level - Triglyceride level - Clinical measures | *Primary outcomes*  At 6 months, there were significant reduction in HbA1c level (mean change = 7.5 mmol/mol, *P*<.001) compared to baseline in IG. No changes were observed in CG.  *Secondary outcomes*  IG had a significant reduction in fat mass (mean change = 1kg, *P*<.001) and android fat mass (mean change = .2kg, *P*<.001) at 6 months compared to baseline. There were no changes in the other anthropometric and clinical measures. There were greater reductions in caloric intake in IG than CG (8.5 vs 2.4%, *P*<.05). IG increased number of exercise per week (*P*<.05), but no changes were found in CG at 6 months. No changes were observed in CG at 6 months compared to baseline. |
| Liu et al (2018), Canada ^56^. | | | | | |
| *Study Design* | *Participant Information* | *Intervention description* | | *Outcome measures* | *Results* |
| *Design*  3-parallel group RCT  *Group Info*   - Expert-driven e-counseling, *n* = 43 - User-driven e-counseling, *n* = 42 - CG, *n* = 43   *Duration*  4 Months  *Measurement points*  Baseline, 4 months  *Attrition rate*   - Expert-driven e-counseling = 9.3% - User-driven e-counseling = 11.9% - CG = 9.3% | *N* = 128  *Sample*  Patients with hypertension  *Mean age*   - Expert-driven = 57.6 (1.5) - User-driven = 57.3 (1.4) - CG = 56.1 (1.4)   *Gender distribution*   - Expert-driven = 48.8% female - User-driven = 52.4% female - CG = 42% female | *Intervention*  E-counseling emails were delivered to the Igs weekly during the 4-month period. The user-driven group set their own health goals and received weekly emails that supported them to attain their set health goals. As for the expert-driven group, they received prescribed specific emails for lifestyle behaviours, which were intended to encourage them to adhere to healthy behaviours. It consisted of predetermined exercise and dietary goals. CG received weekly emails on general info of BP management.  *eHealth features*  Weekly emails for 4 months that aimed to support intervention groups to attain health goals necessary for BP management. Examples include increasing step counts, increase fibre intake. | | *Primary outcomes*   - Systolic BP   *Secondary outcomes*   - Cholesterol levels - Cardiovascular risk (10-year Framingham risk score) - Daily steps - Dietary habits. | *Primary outcomes*  Expert-driven group had the greatest decrease in systolic BP than CG at 4 month follow-up (mean difference = −7.5 mmHg, 95% CI: −12.5 to −2.6, *P*=0.01). No difference in BP change between expert-driven and user-driven groups.  *Secondary outcomes*  Compared with CG, only the expert-driven group showed improvement in pulse pressure (-5.2, 95% CI: -7.3 to -3.0, *P*<.01), cholesterol (-.38, 95% CI: -.59 to -.16, *P*<.01, and Framingham risk score (-3.8, 95% CI: -4.8 to -2.8, *P*<.01. Expert-driven group were more effective than user-driven and CG in increasing daily steps (2,036, 95% CI: 1263 to 2809, *P*<.01) and fruit intake (2.1, 95% CI: 1.3 to 2.8, *P*<.01). |
| Lorig et al (2016), United States ^57^. | | | | | |
| *Study Design* | *Participant Information* | *Intervention description* | | *Outcome measures* | *Results* |
| *Design*  2-group pragmatic trial  *Group Info*   - Web-based group, *n* = 687 - Face-to-face (FTF) group, *n* =170   *Duration*  12 months    *Measurement points*  Baseline, 6 months, 12 months  *Attrition rate*  30.3% (*n*=372/1229) | *N* = 857  *Sample*  Participants with type 2 diabetes  *Mean age*   - Web = 55.8 (8.62) - FTF = 65.6 (9.95)   *Gender distribution*   - Web = 65.4% female (449) - FTF = 72.4% female (123) | *Intervention*  The Better Choices, Better Health-Diabetes (BCBH-D) is a web-based programme that consists of 3 core sections that include education and learning (creating action plans, and providing feedback), discussion section to allow users to interact with each other, and tools section that allow users to track PA level, food and medication intake. FTF group took part in community workshops and covered topics on diabetes self-management.    *eHealth features*  Web-based application | | *Primary outcomes*   - HbA1c levels - Health indicators (e.g., eye, foot, cholesterol, kidney exam) - Aerobic exercise - General health - Psychological outcomes | *Primary outcomes*  At 12 months, 7/7 health indicators (including hbA1c levels) and 7/8 health behaviours significantly improved from baseline in both groups. Between group comparisons showed that web-based group had greater improvements in medication adherence (*P*=.02), while FTF had greater improvements in sleep (*P*=.03) and stress (*P*=.002) |
| Luley et al (2014), Germany ^82^. | | | | | |
| *Study Design* | *Participant Information* | *Intervention description* | | *Outcome measures* | *Results* |
| *Design*  3-group RCT  *Group Info*   - Active Body Control (ABC) group, *n* = 60 - 4sigma Telephone Coaching (4S) group, *n* = 68 - CG, *n* = 62   *Duration*  12 Months  *Measurement points*  Baseline, 4, 8, 12 months  *Attrition rate*   - ABC = 18% - 4S = 17% - CG = 35% | *N* = 184  *Sample*  Patients with metabolic syndrome  *Mean age*   - ABC = 50.3 (7.8) - 4S = 50.3 (8.0) - CG = 50.1 (8.1)   *Gender distribution*   - ABC = 30% female (18) - 4S = 47% female (27) - CG = 47% female(28) | *Intervention*  ABC and 4S groups received an accelerometer which measured their PA level, and allowed users to key in their dietary intake. For the ABC group, feedback and motivational messages were delivered to them via letters, whereas 4S group received them via telephone calls from a health coach. CG received usual care.  *eHealth features*  Wearable technology for all intervention group, and the addition of telephone calls for 4S group. | | *Primary outcomes*   - Weight   *Secondary outcomes*   - BMI - Waist circumference - Blood pressure level - Triglycerides level - Cholesterol levels - Apolipoprotein-B - Uric acid - Alanin-Aminotransferase (ALT) - Aspartat-Aminotransferase (AST) - C-reactive protein (CRP) high sensitivity - HbA1c level - Insulin - Homeostasis model of assessment (HOMA) | *Primary outcomes*  At 12 months, weight reduction was greatest in ABC, followed by 4S and control (*P*<.05).    *Secondary outcomes*  At 12 months, there were significant reduction in all groups for BMI, waist circumference, systolic and diastolic BP, triglycerides, HDL-cholesterol, apolipoprotein-B, ALT, AST, HOMA, and insulin, compared with baseline.  Significant reduction in LDL-cholesterol, CRP and uric acid were found in ABC and 4S group only.  No changes were observed in hbA1c levels, but there was an increase in control.  Compared with control, ABC and 4S greater improvement were found in weight, BMI, waist circumference, and in the ABC group only, triglycerides, CRP, HbA1c, and insulin resistance parameters. For weight, BMI and apolipoprotein-B, ABC showed greater improvement than 4S. |
| Martin et al (2015), United States ^94^. | | | | | |
| *Study Design* | *Participant Information* | | *Intervention description* | *Outcome measures* | *Results* |
| *Design*  2-group pilot RCT  *Group Info*   - SmartLoss (SL) group,  *n* = 20 - Health Education (HE) group, *n* = 20   *Duration*  12 weeks    *Measurement points*  Baseline, 4, 8, 12 weeks    *Attrition rate*  5% | *N* = 40  *Sample*  Healthy adults    *Mean age*   - SL group = 45.6 (2.67) - HE group = 43.3 (2.63)     *Gender distribution*   - SL group = 80% female - HE group = 85% female | | *Intervention*  SL group used mobile app to record their weight, and obtained feedback from a counsellor. They were also prescribed a diet that was consistent with health guidelines. Nutrition education was provided in the toolbox of the app. The HE group received health information via text messages or emails  *eHealth features*  Mobile app and wearables for SL group. Text-messaging and emails for HE group. | *Primary outcomes*   - Weight - Waist circumference - Blood pressure level | *Primary outcomes*  At 12 weeks, mean waist circumference reduction in SL group was -6.9 (SE 1.0) cm, and 1.7 (SE 1.0) cm in HE group, when compared to baseline. SL also had significant improvements  compared to HE on waist circumferences at all  time points (*P*<0.05).  At 12 weeks, SL group reduced weight by -9.4 (0.5%) kg, and HE group by -0.6 (0.5%) kg. SL had significantly greater weight loss (percent of baseline weight) than HE  F_(1,35)_=100.62, *P*<.001.  By week 12, systolic blood pressure change in SL was -6.3(1.77), and -1.5(1.78) in HE. SL had significantly larger reductions in systolic blood pressure compared to HE,  F_(1,40.3)_=4.17, *P*<.05.  There were no significant changes or group-differences in diastolic blood pressure at 12 weeks in both groups (*P*>.05). |
| Mason et al (2018), United States ^58^. | | | | | |
| *Study Design* | *Participant Information* | *Intervention description* | | *Outcome measures* | *Results* |
| *Design*  Feasibility, pre-post study  *Group Info*  All participants took part in intervention  *Duration*  3 months  *Measurement points*  Baseline, 1 month post-intervention  *Attrition rate*  5.8% | *N* = 104  *Sample*  Women who were overweight  *Mean age*  46.2 (14.1)  *Gender distribution*  100% female | *Intervention*  Administered a 28-day mindful eating intervention via a mobile app. Educational content focused on behavioural change and mindfulness to food cravings. Users received feedback about mindful eating, and set reminders to allow them to check in with their hunger and emotional state so that mindless eating was discouraged. They could also set and track their health goals.  *eHealth features*  Mobile application | | *Primary outcomes*   - Weight - Food craving questionnaire trait reduced (FCQ-TR) score - Reward-based eating drive scale (RED) score - Palatable eating motives scale (PEMS) score - Intervention engagement | *Primary outcomes*  Participants who completed the intervention within 3 months (timely completers) significantly reduced weight (mean change = -.82kg, *P*=.019).  There were reductions in craving-related eating behaviour (40.21% reduction, *P*<.001) and self-reported overeating behaviour (*P*<.001). Scores of FCQ-TR, RED and PEMS were reduced post-intervention (*P*<.001) compared with baseline.  On average, users accessed the app twice a day for an average of 11.74 (8.89) mins per day. |
| McCarroll et al (2015), United States ^59^. | | | | | |
| *Study Design* | *Participant Information* | *Intervention description* | | *Outcome measures* | *Results* |
| *Design*  Feasibility, pre-post study  *Group Info*  All participants took part in intervention  *Duration*  One month  *Measurement points*  Baseline, 1 month  *Attrition rate*  30% | *N* = 50  *Sample* Overweight or obese endometrial and breast cancer survivors  *Mean age*  58.4 (10.3)  *Gender distribution*  100% female | *Intervention*  The nutritional component focused on limiting daily carbohydrates intake and increasing daily fibre intake. Participants are able to monitor their carb intake through the app. Participants received motivational push-notifications and encouraged to log daily meals and PA levels.  *eHealth features*  LoseIt! Mobile application Participants are able to record food intake and PA level on the app. Push notifications was sent to motivate participants to log in meals and PA levels. | | *Primary outcomes*   - Weight - BMI - WC - Functional Assessment of Cancer Therapy-General (FACT-G), evaluates physical, social/family, emotional, and functional well-being - Weight Efficacy Life-Style Questionnaire (WEL) - Nutrient quality of the participant’s daily food intake log | *Primary outcomes*  Compared to baseline, there were significant reduction in weight [97.3 (22.5) to 95 (22.1), *P*<.001)], BMI [36.4 (8.1) to 35.6 (8.0), *P*<.001], and WC [106.6 (16.8) to 103.4 (17.4), *P*<.001]. There was significant improvement of WEL scores from 99.38 (41.8) to 120.19 (47.1), *P*=.043. No significant changes of FACT-G scores from baseline [50.47 (13.3)] and one-month follow up [44.35(19.9)], *P*=.152. No significant changes in the nutrient quality of the macronutrient categories such as carbohydrates (*P*=.73), fats *(P*=.18), protein (*P* = .23), fiber (*P*=.28), and calories (*P=.*26). No *s*ignificant changes in PA patterns (*P*=1.00). |
| McKenzie et al (2017), United States ^60^. | | | | | |
| *Study Design* | *Participant Information* | *Intervention description* | | *Outcome measures* | *Results* |
| *Design*  2-arm pre-post study  *Group Info*  All participants took part in the intervention  *Duration*  10 weeks  *Measurement points*  Baseline, 10 weeks    *Attrition rate*  9.2% | *N* = 262    *Sample*  Adults with type 2 diabetes  *Mean age*  54 (8)    *Gender distribution*  66.8% female (175) | *Intervention*  The Virta Clinic is an e-health service that aimed to provide educational content, biometric feedback, and peer support for users. Content included management of diabetes in relation to diet, nutrition, and behavioural change techniques.  *eHealth features*  Web-based application | | *Primary outcomes*   - hbA1c level - Fasting blood glucose level - BMI - Weight - Blood pressure level - Cholesterol level - Triglycerides level - Serum creatinine - ALT - AST - Alkaline phosphatase - C-reactive protein - White blood cell - Change in medication prescription | *Primary outcomes*  The level of significance was set to *P*<.003 for multiple comparisons.  All outcomes were significantly reduced from baseline to follow-up (all *P*<.001), with the exception of total cholesterol (*P*=.009), LDL-C (*P*=.32), HDL-C (*P*=.33), and C-reactive protein (*P*=.01). 42.7% of participants decreased their medication prescription, 8% stopped their medication, 5% had new or increased prescription, and 33.6% had no change in their prescription. |
| Michaelides et al (2018), United States ^61^. | | | | | |
| *Study Design* | *Participant Information* | *Intervention description* | | *Outcome measures* | *Results* |
| *Design*  Pre-post pilot study  *Group Info*  All participants took part in the intervention.   - Starters, *n* = 59 - Completers, *n* = 47 - Maintenance completers, *n* = 32   *Duration*  65 weeks  *Measurement points*  Baseline, 24 weeks, 65 weeks  *Attrition rate*  20.3% dropped out from the main core intervention | *N* = 59  *Sample*  Participants with type 2 diabetes  *Mean age*   - Starters = 51.27 (9.25) - Completers = 51.45 (9.47) - Maintenance completers = 51.34 (9.55)   *Gender distribution*   - Starters = 81% female (48) - Completers = 79% female (37) - Maintenance completers = 81% female (26) | *Intervention*  Participants used the Noom app which allowed users to interacts with a health coach, set daily health challenges, provide self-monitoring tools to log their weekly PA and diet intake, receive feedback about their food choices and provide educational content and motivational messages to continue using the app that will help them make positive lifestyle changes.  *eHealth features*  Mobile application | | *Primary outcomes*   - Weight - BMI - App usage | *Primary outcomes*  At 65 weeks, mean weight loss for the Starters group were -5.93kg (6.78), -7.05 kg (7.10) for Completers, and -8.78kg (7.71) for Maintenance Completers (all *P*<.001) compared with baseline. BMI was also reduced by -2.12 (2.43) in Starters, -2.53 (2.54) in Completers, and -3.14 (2.76) in Maintenance Completers.  Results show that the longer users interacted with the app, the greater positive health changes they experienced. |
| Miller et al (2017), USA ^95^ | | | | | |
| *Study Design* | *Participant Information* | | *Intervention description* | *Outcome measures* | *Results* |
| *Design*  2-group pre-post study  *Group Info*   - IG,  n = 722 - CG, n = 800   *Duration*  6 months    *Measurement points*  Baseline, 6 months    *Attrition rate*  Did not specify | *N* = 722  *Sample*  Adults with mixed dyslipidaemia  *Mean age*   - 59 (13)     *Gender distribution*   - IG = 62.7% female - CG = 64% female | | *Intervention*  IG took part in health coaching sessions delivered by dietitians. These sessions promoted healthy lifestyle such as having a healthy diet and regular exercise. Participants also had dietary recall, and set health goals for weight loss. CG received standard care.  *eHealth features*  Telephone-based health coaching. | *Primary outcomes*   - Weight - BMI - Cholesterol level | *Primary outcomes*  At 6 months, there were no significant changes in IG in their weight [mean Δ from 82.6 (11.7)kg to 82.6 (18.2)kg, *P*=0.75] and BMI [mean Δ from 30.4(2.3) to 30.5(2.9), *P*=0.78] compared to baseline.  There were no significant changes to LDL-C and HDL-C levels post-intervention (all *P*>.05).  IG significantly reduced serum concentrations of apoB100, and decreased cholesterol content of VLDL subfractions compared to controls (*P*<.05). |
| Moin et al (2018), United States ^62^. | | | | | |
| *Study Design* | *Participant Information* | *Intervention description* | | *Outcome measures* | *Results* |
| *Design*  Pre-post, non-randomized comparative study  *Group Info*   - Online DPP, *n* = 180 - VA-DPP, *n* = 198 - MOVE!, *n* = 64   *Duration*  12 months  *Measurement points*  Baseline, 6 months, 12 months    *Attrition rate*  25.6% from online DPP did not complete >1 session | *N* = 442    *Sample*  Participants who had obesity  *Mean age*   - Online DPP = 60.2 (10.6) - VA-DPP = 59.2 (8.9) - MOVE! = 60.5 (9.1)   *Gender distribution*   - Online DPP = 35.6% female (64) - VA-DPP = 13.1% female (26) - MOVE! = 6.3% female (4) | *Intervention*  The online diabetes prevention programme (DPP) provided users with education on nutrition and exercise, allowed users to interact with a health coach, send messages to a group chat, set weight goals, and self-monitor their weight. VA-DPP and MOVE! Were health intervention programmes that were conducted prior to the online DPP study, and there were no e-health components involved.  *eHealth features*  Web-based application, and a wireless scale for weight recording. | | *Primary outcomes*   - Weight - App usage | *Primary outcomes*  At 12 months, Online DPP significantly reduced weight by -4.0kg (95%CI: -4.9 to -3.0), *P*<.001), compared with baseline. Online DPP reduced more weight as compared to the other groups. |
| Mundi et al (2015), United States ^63^. | | | | | |
| *Study Design* | *Participant Information* | *Intervention description* | | *Outcome measures* | *Results* |
| *Design*  Pre-post feasibility trial study  *Group Info*  All participants took part in the intervention  *Duration*  4 months (15 weeks)  *Measurement points*  Baseline, 15 weeks  *Attrition rate*  33% | *N* = 30  *Sample*  Patients who were undergoing bariatric surgery  *Mean age*  41.3 (11.4)  *Gender distribution*  90% Female | *Intervention*  The mobile app sent timely messages, depending on user’s lifestyle patterns, that were framed based on several educational modules. The key domains of messaging included a nutritional and PA modules, which provided information about healthy eating (diet, healthier food choices, servings and portions, food labels) and being active (exercise basics, fitness plans, maintaining motivation). Supportive messages to encourage users to maintain health behaviours were also delivered through the app.  *eHealth features*  Mobile application sent educational videos and messages, and supportive messages. | | *Primary outcomes*   - Weight - BMI - Bariatric surgery knowledge questionnaire (self-developed) - International Physical Activity Short Form | *Primary outcomes*  At study completion, there were significant reductions in weight [127.4 (27.5) at baseline vs 123.9 (28.6) at completion, *P*=.006], and BMI [46.3 (7.4) at baseline vs 45.1 (8.5) at completion, *P*<.001]. There was an increase in vigorous activity levels from 25.5 (43.9) min to 49.4 (51.1) min, *P*=.04.  There were no changes in other outcomes (all *P*>.05). |
|  | | | | | |
| Nepper et al (2019), USA ^96^ | | | | | |
| *Study Design* | *Participant Information* | | *Intervention description* | *Outcome measures* | *Results* |
| *Design*  2-group pre-post study  *Group Info*   - IG,  n = 40 - CG, n = 39   *Duration*  12 weeks    *Measurement points*  Baseline, 12 weeks    *Attrition rate*  11.4% | *N* = 79  *Sample*  Patients with Type 2 diabetes  *Mean age*   - IG = 58.0 (10.6) - CG = 55.7 (12.2)     *Gender distribution*   - IG = 65.3% female - CG = 67.1% female | | *Intervention*  IG received educational text messages on diabetes self-care activities,  cardiovascular disease (CVD) risk awareness, and home food availabilities related to food choices  among patients with type 2 diabetes. CG did not receive any text messages.  *eHealth features*  Text messages | *Primary outcomes*   - Diabetes self-care activities - Cardiovascular disease (CVD) risk awareness - Physical activity (PA) - Dietary intake - Home Food Self-Inventory   *Secondary outcomes*   - Satisfaction regarding feasibility and usefulness of intervention | *Primary outcomes*  At 12 weeks, there were no between-group differences in changes of scores on diabetes self-care activities.  Regarding CVD risk awareness, there was a statistically significant improvement for IG compared to CG for level of concern [IG: Δ from 1.01(0.23) to 0.21(1.26) compared to CG: Δ from 1.27(0.25) to 0.94(0.26); 0.58(0.29); 57% increase; *P*=0.04].  Compared to CG, IG had a significant increase in total PA (MET - metabolic equivalent of task minutes per week) [IG=5548; CG=2877; Δ 16%; *P*=0.02) and in moderate/vigorous PA [IG=3163; CG=405; Δ 80%; *P*=0.006) compared to baseline.  Comparing IG and CG, there were no statistically significant differences in intakes of relevant nutrients.  *Secondary outcomes*  94% (n=35) of IG said intervention was useful and would highly recommend to others with type 2 diabetes. |
| Nolan et al (2018), Canada ^64^. | | | | | |
| *Study Design* | *Participant Information* | *Intervention description* | | *Outcome measures* | *Results* |
| *Design*  2-arm parallel RCT  *Group Info*   - IG, *n* = 133 - CG, *n* = 131   *Duration*  12 months  *Measurement points*  Baseline, 4 months, 12 months.    *Attrition rate*   - IG = 26.3% - CG = 25.2% | *N* = 264    *Sample*  Patients with hypertension  *Mean age*   - IG = 58 - CG = 57.2     *Gender distribution*   - IG = 56% female (74) - CG = 61% female (80) | *Intervention*  IG received e-counselling services that aimed to provide motivational and cognitive-behavioural skills to promote healthy lifestyle (including nutrition), and medication adherence. CG received usual care and information about hypertension management.  *eHealth features*  Web-based system | | *Primary outcomes*   - Blood pressure level - Pulse pressure level - Cholesterol level - Framingham CVD risk | *Primary outcomes*  At 12 months, both groups reduced their systolic BP, diastolic BP, and pulse pressure level compared to baseline, with IG having greater reduction in systolic BP (*P*=.02) and pulse pressure (*P*=.04) compared to CG. No changes in cholesterol level observed in both groups at 12 months. Framingham risk was reduced significantly in IG but not in CG at 12 months (*P*=.02) |
| Orlandoni et al (2016), Italy ^83^. | | | | | |
| *Study Design* | *Participant Information* | *Intervention description* | | *Outcome measures* | *Results* |
| *Design*  2-group RCT  *Group Info*   - IG, *n*= 100 - CG, *n* = 88     *Duration*  6.7 months  *Measurement points*  Baseline, monthly visits for an average of 6.7 months per patient  *Attrition rate*  Not specified | *N* = 188  *Sample*  Patients treated with home enteral nutrition (HEN)  *Mean age*   - IG = 86.45 (7.03) - CG = 84.36 (7.05)   *Gender distribution*   - IG = 72% female (72) - CG = 76.14% female (67) | *Intervention*  Home visiting staff used a tablet for a video consultation with a physician during their monthly home visits with the patient. The video consultation involves examining the patient, and changing medication or nutrition regimen if necessary. CG received regular home visits and care by a home visiting staff.  *eHealth features*  Video call | | *Primary outcomes*   - Incidence rate of complications - Incidence rates of outpatient hospital visits - Incidence rates of hospitalisation | *Primary outcomes*  Incidence rates for 365 days/patient were observed. IG had lower incidence rates of overall complications compared with CG (IR .011, 95%CI: .098 to .122 in IG vs IR .16, 95%CI: .145 to .175 in CG, *P<*.0001). Incidence rate of gastrointestinal and metabolic complications were higher in CG than in IG (*P*<.0001). There were no differences in the incidence rates of outpatient hospital visits and hospitalisation. |
| Peimani et al (2015), Iran ^97^ | | | | | |
| *Study Design* | *Participant Information* | | *Intervention description* | *Outcome measures* | *Results* |
| *Design*  3 group RCT  *Group Info*   - Tailored SMS (IG-T-SMS), n = 50 - Non-tailored SMS (IG-NT-SMS), n = 50 - Control group (CG), n = 50   *Duration*  12 weeks    *Measurement points*  Baseline, 12 weeks    *Attrition rate*  0% | *N* = 150  *Sample*  Patients with type 2 diabetes  *Mean age*   - IG-T-SMS = 49.78(9.76) - IG-NT-SMS = 53.26(10.49) - CG = 54.56(9.88)     *Gender distribution*   - IG-T-SMS = 46% female - IG-NT-SMS = 44% female - CG = 48% female | | *Intervention*  IG-T-SMS and IG-NT-SMS groups received educational messages about self-management of diabetes. The topics included were on nutrition, exercise, medication adherence, and blood glucose monitoring. IG-T-SMS group received tailored messages that focused on participants’ barriers on diabetes self-management. CG received usual care.  *eHealth features*  Text messaging | *Primary outcomes*   - BMI - FBS (fasting blood sugar levels) - HbA1C levels - Lipid profile - Self-Care Inventory(SCI) - Diabetes self-care barriers (DSCB) - Diabetes Management Self-Efficacy Scale(DMSES) | *Primary outcomes*  At 12 weeks, there was a significant decline in BMI in IG-T-SMS [from 27.71 (5.29) to 27.14 (5.51), *P*<.001] and IG-NT-SMS [from 27.40 (4.73) to 26.90(4.57); *P=*.002. There was an increase in BMI for CG [from 27.92 (4.97) to 28.21 (5.15), *P*= 0.045].  There was significant reduction of FBS in IG-T-SMS [from 172.44 (70.74) to 152.54 (81.09), *P=*.003] and IG-NT-SMS [from 169.54 (70.87) to 147.82 (47.27), *P*=.026]. No significant changes in CG [from 166.94 (67.52) to 165.32 (57.85), *P*=.85]  No significant changes in HbA1c and other lipid profiles in all groups (all *P*>.05)  At 12 weeks, SCI score significantly increased in IG-T-SMS [from 55.41 (10.54) to 65.37 (10.26), *P*<.001] and IG-NT-SMS [from 55.43 (10.67) to 65.79 (9.99), *P*<.001]. There was an increase in SCI score in CG [from 54.57 (9.13) to 49.98 (11.25), *P*<.001].  DSCB decreased in IG-T-SMS [from 45.60 (11.06) to 31.42(11.80), *P*<.001] and in IG-NT-SMS [from 42.98 (10.20) to 29.24 (11.55), *P*<.001]. DSCB increased in CG [from 49.78 (10.62) to 57.56 (12.50), *P*<.001]. DMSES decreased in IG-T-SMS [from 57.40 (12.90) to 43.77(11.50), *P*<.001], and in IG-NT-SMS [from 53.63 (12.39) to 39.78 (8.67), *P*<.001]. DMSES increased in CG [from 58.95 (11.86) to 66.95 (11.38), *P*<.001] |
| Pfaeffli Dale et al (2015), New Zealand ^33^. | | | | | |
| *Study Design* | *Participant Information* | *Intervention description* | | *Outcome measures* | *Results* |
| *Design*  2-group parallel RCT  *Group Info*   - Intervention, *n*= 61 - CG, *n* = 62     *Duration*  6 months  *Measurement points*   - Primary outcomes were measured at baseline, 3 months, and 6 months. - Secondary outcomes were measured at baseline, and at 6 months.   *Attrition rate*   - Intervention = 6.56% - CG = 4.84% | *N* = 123  *Sample*  Patients with coronary heart disease  *Mean age*   - Intervention = 59 (10.5) - CG = 59.9 (11.8)   *Gender distribution*   - Intervention = 21% female - CG = 16% female | *Intervention*  All participants received usual care of cardiac rehabilitation. In addition to usual care, intervention group received 24-week mHealth programme. It sends text messages to encourage participants to adopt healthy lifestyle changes such as consuming 5 servings of fruits and vegetables a day, smoking cessation, and have regular exercise. The mHealth intervention was based on social cognitive theory, with self-efficacy as the key mediator (those with higher self-efficacy levels will be better able to adopt and maintain health behaviours). Participants received one message a day and had access to supporting website but from week 13 to 24, they only received 5 messages per week. Intervention group also received pedometer to track PA.  *eHealth features*  Text4Heart, Text messaging encouraging them to adopt and maintain health behaviours. Messages also provided links to supporting website whereby they may receive additional information and help. | | *Primary outcomes*   - Health behaviours measured were smoking habit, fruit and vegetable intake, alcohol intake, and PA. - Adherence to recommended health guidelines measured by the European Prospective Investigation into Cancer (EPIC)-Norfolk Prospective Population Study   *Secondary outcomes*   - BP - Cholesterol - BMI - Waist-to-hip ratio - CVD risk score (Framingham score) - Medication adherence - Psychological outcomes (including overall illness threat, hospital anxiety, hospital depression, and self-efficacy). | *Primary outcomes*  IG increased adherence to health behaviours from 33% at baseline to 59% at 3 months, but plateaued with 53% at 6 months. CG increased adherence from 27% at baseline to 37% at 3 months, and 39% at 6 months. A significant treatment effect was found in ounse of the intervention was observed at 3 months (adjusted odds ratio, AOR, 2.55, 95% CI 1.12 to 5.84; *P*=.03), but not at 6 months (AOR 1.93, 95% CI 0.83 to 4.53; *P*=.13).  *Secondary outcomes*  At 6 months, IG reported a significantly greater medication adherence score (mean difference = 0.58, 95% CI 0.19 to 0.97, *P*=.004) and greater hospital anxiety (mean difference = 1.18, 95% CI: .28 to 2.08, *P*=.01), as compared to CG.  No other differences observed in other outcomes |
| Ramadas et al (2018), Malaysia ^98^. | | | | | |
| *Study Design* | *Participant Information* | | *Intervention description* | *Outcome measures* | *Results* |
| *Design*  2-group RCT  *Group Info*   - IG,  n = 62 - CG, n = 66     *Duration*  6 months    *Measurement points*  Baseline, 6 months, 12 months    *Attrition rate*  0.03% | *N* = 132  *Sample*  Patients with type 2 diabetes  *Mean age*   - IG = 49.6 (10.7) - CG = 51.5 (10.3)     *Gender distribution*   - IG = 37.9% female - CG = 24.2% female | | *Intervention*  myDIDeA web-based intensive dietary intervention consisted of lesson plans that were targeted towards encouraging healthy eating behaviours such as reducing sugar intake, improving eating out habits, and increasing fruit and vegetable consumption. IG group were reminded to log in if they had been inactive with their lesson plans. CG received standard care.  *eHealth features*  Web-based application | *Primary outcomes*   - Dietary Knowledge, Attitude and Behaviour (DKAB) scores - Dietary Stages of Change (DSOC) - Fasting blood glucose (FBG) - HbA1c | *Primary outcomes*  At 12 months, both IG and CG improved DKAB score compared to baseline [IG: from 34.2(5.2) to 45.2(8.0) to 54.0(8.7), *P*<.001 and CG: from 33.7(5.5) to 41.3 (7.7), *P*<.001]. IG significantly improved DKAB score compared with CG at 12 months (*P*<.001).  IG significantly improved DSOC score [from 193.3(14.6) to 197.5(16.7) to 199.7(18.2); *P*=.046], but no changes were observed in CG [from 191.2(16.2) to 191.5(15.1), *P*=.99]. IG significantly improved DSOC score compared with CG at 12 months (*P*=.007).  Both IG and CG improved FBG levels at 12 months compared to baseline [IG: from 8.9(3.9) to 7.9(2.5), *P*=0.015; CG: from 8.3(2.9) to 7.7(2.6), *P*=.12]. No significant difference between IG and CG (*P*=0.345). Both IG and CG improved HbA1c level at 12 months compared to baseline [IG: from 9.1(2.0) to 8.5(1.8), *P*=.004; CG: from 8.9(1.9) to 8.4(2.2), *P*=.001]. No significant difference between IG and CG (*P*=0.51). |
| Recio-Rodriguez et al (2016), Spain ^85^. | | | | | |
| *Study Design* | *Participant Information* | *Intervention description* | | *Outcome measures* | *Results* |
| *Design*  2-arm parallel RCT  *Group Info*   - Counseling + app group, *n* = 415 - Counseling only, *n* = 418   *Duration*  3 months  *Measurement points*  Baseline, 3 months  *Attrition rate*   - Counseling + app group = 8.67% - Counseling only = 7.66% | *N* = 833    *Sample*  Healthy older adults  *Mean age*   - Counseling+app group = 51.4 (12.1) - Counseling only = 52.3 (12)   *Gender distribution*   - Counseling+app group = 60% female - Counseling only = 64.1% female | *Intervention*  Counseling on PA and Mediterranean diet were provided for all participants.  Participants can log food intake and exercise levels on the app. Personalised dietary recommendation were provided for users. The app provided daily summary reports of food intake and PA performed, and generated plans for the following days with an aim to improve diet and increase PA.  *eHealth features*  Mobile application | | *Primary outcomes*   - Adherence to Mediterranean diet was measured using the Mediterranean Diet Adherence Screener Questionnaire - PA level measured by the Physical Activity Recall (PAR) questionnaire and an accelerometer   *Secondary outcomes*   - BP - WC - BMI | *Primary outcomes*  Compared to baseline, both groups increased adherence to the Mediterranean diet at 3 months, with 8.4% of participants in the Counseling+app group (95% CI: 3.4 to 13.5, *P*=.001) and 10.4% of participants from Counselling only group (95% CI: 5 to 15.8, *P*<.001). Both groups had significant improvements in the adherence to Mediterranean diet score, with an increase of 0.42 in Counseling+app group (95% CI: 0.24 to 0.6, *P*<.001) and 0.53 in Counselling only group (95% CI: 0.35 to 0.71, *P*<.001).  No between-group difference in improvement (mean difference -0.02, 95% CI: -0.25 to 0.21, *P*=.86). PAR outcomes such as moderate -to-vigorous physical activity (MVPA) increased in Counseling+app group (mean 29.1, 95% CI 4.9 to 53 min/week; *P*=.02) but not in the Counselling only group (mean 11.7, 95% CI –14.6 to 38.1 min/week; *P*=.38). No difference in MVPA between both groups (mean difference 9.5, 95% CI: -22.6 to 41.6, *P*=.56). |
| Recio-Rodriguez et al (2018), Spain ^84^. | | | | | |
| *Study Design* | *Participant Information* | *Intervention description* | | *Outcome measures* | *Results* |
| *Design*  Similar to Recio-Rodriguez et al (2016)  *Measurement points*  Baseline, 3 months, 12 months  *Attrition rate*   - Counseling + app group = 15.2% - Counseling only = 13.2% | See Recio-Rodriguez et al (2016) | See Recio-Rodriguez et al (2016) | | *Primary outcomes*   - Nutritional composition of food intake measured by Food Frequency Questionnaire | *Primary outcomes*  At 12 months, both groups decreased their energy intake with mean difference of -114 (95% CI: -191 to -36, *P*<.001) and -108 (95% CI: -184 to -31, *P*<.001) respectively. Counseling + app group reported higher intake of carbohydrates (1.1%, 05% CI: 0.1 to 1.9, *P*<.05), and lower intake of fats (-1.0%, 95% CI: -1.8 to -0.1, *P*<.05) and saturated fats (-0.6%, 95% CI: -0.9 to -0.3, *P*<.001) when compared to Counseling group. |
| Shahid et al (2014), Pakistan ^99^ | | | | | |
| *Study Design* | *Participant Information* | | *Intervention description* | *Outcome measures* | *Results* |
| *Design*  2-group RCT    *Group Info*   - IG,  *n* = 220 - CG, *n* = 220   *Duration*  4 months    *Measurement points*  Baseline, 4 months    *Attrition rate*  Did not specify | *N* = 440  *Sample*  Rural diabetic patients with T2DM  *Mean age*   - IG = 48.95 (8.83) - CG = 49.21 (7.92)     *Gender distribution*   - IG = 38.6% female - CG = 38.6% female | | *Intervention*  IG received regular feedback (every 15 days) based on blood glucose readings via mobile phone. CG received standard care.  *eHealth features*  Mobile phone | *Primary outcomes*   - Systolic blood pressure - Diastolic blood pressure - Hypertension - BMI - HbA1c - LDL-Cholesterol level - Number of participants following diet plan - Number of participants who were physically active | *Primary outcomes*  At 4 months, there were significant reductions in systolic BP and diastolic BP in IG [systolic BP: mean Δ -= 2.88(0.5), *p*<.001; diastolic BP: mean Δ = - 5.43(0.5); *P*<0.001] compared to baseline. No significant changes were found in CG [systolic BP: mean Δ = -1.22(0.65), *P*=.062; diastolic BP: mean Δ = 0.59(0.59), *P*=.321]. Significant reduction in hypertension for both [IG: mean Δ = -19.1%, *P*<.001; CG: mean Δ = -7.3%; *P*<.001]. Significant increase in BMI for both groups at 4 months when compared to baseline [IG: mean Δ = 0.96(09), *P*<.001; CG: mean Δ = 1.02(0.09), *P*<.001]. At 4 months, significant reduction in HbA1c for IG, but an increase for CG [IG: mean Δ = -1.46(.07), *P*<.001; CG: mean Δ = 0.48(0.04); *P*<.001]. Significant reduction in LDL for both groups [IG: mean Δ = -23(1.4), *P*<.001; CG: mean Δ = -9.04(0.77), *P*<.001]. There was an increase in number of participants in IG for following a diet plan (Δ= 26.3%; *P*<.001), and for being physically active (Δ= 28.1%; *P*<.001). No changes were found in CG in number of participants who followed a diet plan (Δ = 2.3%; *P*=0.522), and those who were physically active (Δ = 2.5%; *P*=0.472). |
| Santo et al (2018), Australia ^39^. | | | | | |
| *Study Design* | *Participant Information* | *Intervention description* | | *Outcome measures* | *Results* |
| *Design*  2-arm parallel RCT  *Group Info*   - IG, *n* = 338 - CG, *n* = 351   *Duration*  6 months  *Measurement points*  Baseline, 6 months  *Attrition rate*  21/710 (2.92%), did not specify from which group. | *N* = 710  *Sample*  Patients with coronary heart diseases  *Mean age*  57.6 (9.18)  *Gender distribution*  18% female | *Intervention*  IG received 4 text messages weekly for 6 months in addition to usual care. Text messages were advice and motivational reminders to encourage a change in a healthy lifestyle. The contents of the message were on general cardiovascular health, smoking cessation, physical activity, and diet. With regard to the messages that focused on diet, they were sent to encourage patients to eat increase their fibre and fish intake, and decrease unhealthy fats and high-salt foods, and having healthier methods of cooking.  *eHealth features*  Text messages via mobile phone | | *Primary outcomes*   - Dietary intake (fruits, vegetables, fish, oil, fat, and salt intake, measured by 10-item survey based on WHO STEPS instrument (World Health Organisation STEPwise approach to chronic disease risk factor Surveillance) | *Primary outcomes*  At 6 months, IG had significantly more patients adhering to 4 or more recommendations as compared with CG (relative risk =1.23, 95% CI: 1.15 to 1.31, *P*<.001). IG reported consuming more vegetables (mean difference = 5.94, 95% CI: 4.61 to 7.26, *P*<.001), fruits (mean difference = 3.8, 95% CI: 2.78 to 4.83, *P*<.001) and fish (mean difference=69.7, 95% CI:40.68 to 98.72, *P*<.001), and lower amounts of takeaway foods (mean difference=-.87 95% CI:-1.22 to -.51, *P*<.001). |
| Saslow et al (2017), United States ^65^. | | | | | |
| *Study Design* | *Participant Information* | *Intervention description* | | *Outcome measures* | *Results* |
| *Design*  2-group RCT    *Group Info*   - IG, *n* = 12 - CG, *n* = 13   *Duration*  32 weeks  *Measurement points*  Baseline, 16 weeks, 32 weeks  *Attrition rate*   - IG = 8% - CG = 46% | *N* = 25    *Sample*  Participants with type 2 diabetes  *Mean age*   - IG = 53 (10.2) - CG = 58.2 (6.7)   *Gender distribution*   - IG = 50% female - CG = 69% female | *Intervention*  IG received an online intervention which encouraged very low-carbohydrate ketogenic diet. They were also encouraged to make positive lifestyle changes. CG received were educated on the “create your plate” diet. Both IG and CG received emails with at different frequency.  *eHealth features*  Emails with links to online resources | | *Primary outcomes*   - hbA1c level   *Secondary outcomes*   - Weight - Triglycerides level - Cholesterol level - Diabetes Distress Scale - CESD scale - mDES scale - Self-report physical health - Diet intake | *Primary outcomes*  At 32 weeks, IG had greater reduction in hbA1c level compared with CG (difference by -.5, 95%CI: -.8 to -.2), *P*=.002.    *Secondary outcomes*  At 32 weeks, IG had greater reduction in weight (-9.6kg, 95%CI:-14 to -5.3, *P*<.001), triglycerides level (-53.9, 95%CI:-93.6 to -14.2, p=.01), total grams of nonfiber carbs (-107.9, 95%CI: -164.6 to -51.3, *P*<.001), and total grams of sugar (-32.2, 95%CI: -53.9 to -10.6, *P*<.001).  No between group differences were found at 32 weeks for the other outcomes. |
| Saslow et al (2018), United States ^66^. | | | | | |
| *Study Design* | *Participant Information* | *Intervention description* | | *Outcome measures* | *Results* |
| *Design*  Pre-post study    *Group Info*  All participants took part in the intervention  *Duration*  10 weeks  *Measurement points*  Baseline, 12 months  *Attrition rate*  29.2% | *N* = 1000    *Sample*  Adults with type 2 diabetes    *Mean age*  56.1 (15.7)  *Gender distribution*  59.3% female | *Intervention*  The Low-Carb Program was delivered digitally and provided nutrion-focused intervention that included education on nutrition and diet, allowed users to self-monitor and track their health data, set health goals, reduce carbohydrate intake, and obtained feedback about their progress.    *eHealth features*  Web-based platform | | *Primary outcomes*   - HbA1c level - BMI - Medication prescription | *Primary outcomes*  At 12-months follow-up, all participants significantly reduced hbA1c levels by .76% (1.46), *P*<.001. They also reduced weight by 4.35 kg (12.93), *P*<.001. Those who did not complete the intervention had no change in their hbA1c level (*P*=.01) and weight (*P*=.53)  Of those who were prescribed to at least one hypoglycemic medication at baseline, 40.4% (289/714) were able to stop one or more medication. |
| Sepah et al (2017), United States ^67^. | | | | | |
| *Study Design* | *Participant Information* | *Intervention description* | | *Outcome measures* | *Results* |
| *Design*  Pre-post study    *Group Info*  All participants took part in the intervention  *Duration*  Participants had access to the DPP for as long as they like  *Measurement points*  Baseline, 16 weeks, 1, 2, and 3 years  *Attrition rate*  54% were not retained at 3-year follow-up. | *N* = 220    *Sample*  Adults with prediabetes    *Mean age*  43.6 (12.4)  *Gender distribution*  82.7% female | *Intervention*  The Diabetes Prevention Programme (DPP) was translated for cultural adaptation, and made accessible via web-based platform and mobile devices. It allowed users to record and monitor their health data, access to a health coach, peer support, and educational content on achieving positive lifestyle changes such as nutrition and exercise.    *eHealth features*  Web-based platform or mobile devices. | | *Primary outcomes*   - Glycosylated haemoglobin (A1c) level - Programme attendance - Weight | *Primary outcomes*  Those who completed four or more sessions were called Starters, while those who completed nine or more lessons were known as Completers. At 3-year follow-up, Starters lost 6.7kg(2.0), *P*=.0009, and Completers lost 6.3kg(2.1), *P*=.0024, compared with baseline. Both also reduced A1c levels by .31(.09), *P*=.0008, and by .33(.09), *P*=.0005, respectively. |
| Sun et al (2019), China, ^100^. | | | | | |
| *Study Design* | *Participant Information* | | *Intervention description* | *Outcome measures* | *Results* |
| *Design*  2-group RCT    *Group Info*   - IG,  *n* = 44 - CG, *n* = 47     *Duration*  6 months    *Measurement points*  Baseline, 3, and 6 months    *Attrition rate*  Did not specify | *N* = 91  *Sample*  Patients with type 2 diabetes  *Mean age*   - IG = 67.9 (IQR: 66-71) - CG = 68.04 (IQR: 66-72)     *Gender distribution*   - IG = 56.8% female - CG = 61.7% female | | *Intervention*  Participants in IG were given an mHealth management app to upload glucometer data via Bluetooth. Medical advice and reminders are sent to patients via personal messaging app. IG had to key in physical activity data via text messaging. CG received standard care.    *eHealth features*  Mobile app and text messages. | *Primary outcomes*   - HbA1c - Postprandial blood glucose (PBG) level - Cholesterol level - BMI - Blood pressure level - Fasting blood glucose (FBG) level - Triglyceride level - Satisfaction | *Primary outcomes*  At 6 months, both IG and CG significantly reduced and improved their hbA1c level (*P*<.05) compared to baseline. IG significantly improved more than CG at 6 months [IG: 6.84% (.76) vs CG: 7.22% (.87), *P*=.02).  IG also significantly reduced PBG levels at 6 months as compared to CG (IG: 10.62mmol/L (2.07) vs CG: 12.19 mmol/L (2.54), *P*=.004).  There were no group-differences for the other outcomes at 6 months (all *P*>.05). |
| Vadheim et al (2017), United States ^68^. | | | | | |
| *Study Design* | *Participant Information* | *Intervention description* | | *Outcome measures* | *Results* |
| *Design*  2-group pre-post study    *Group Info*   - IG, *n* = 256 - CG, *n* = 638   *Duration*  2008-2015 (to allow all 7 towns of Miles City to take part)  *Measurement points*  Data collected were categorised to baseline, 0-6 weeks, 7-13 weeks, and 14-16 weeks.  *Attrition rate*  Not specified | *N* = 894    *Sample*  Overweight adults  *Mean age*   - IG = 52 (11.4) - CG = 51.6 (12.4)   *Gender distribution*   - IG = 87% female (221) - CG = 83% female (529) | *Intervention*  The diabetes prevention programme (DPP) was delivered through telehealth videoconference to IG. DPP aimed to improve lifestyle behaviours by encouraging participants to self-monitor their diet, weight, increasing PA levels and reducing fat intake. CG took part in the DPP intervention on-site.  *eHealth features*  Videoconferencing | | *Primary outcomes*   - Programme attendance - PA level - Weight - BMI | *Primary outcomes*  There were no differences in programme attendance between IG and CG (*P*>.05). There were no between-group differences in PA levels (*P*=.44), weight loss (*P*=.10), and BMI reduction (*P*=.45). |
| van Doorn-van Atten et al (2018), Netherlands ^86^. | | | | | |
| *Study Design* | *Participant Information* | *Intervention description* | | *Outcome measures* | *Results* |
| *Design*  2-group pre-post study    *Group Info*   - IG, *n* = 97 - CG, *n* = 107   *Duration*  6 months  *Measurement points*  Baseline, 4.5 months, 6 months  *Attrition rate*  20% | *N* = 204    *Sample*  Healthy older adults  *Mean age*   - IG = 78.4 (7.2) - CG = 81 (7.9)   *Gender distribution*   - IG = 66% female - CG = 76.6% female | *Intervention*  PhysioDom Home Dietary Intake Monitoring (HDIM) intervention consisted of telemonitoring of health (weight, step count, and BP), nutrition education (tailored and non-tailored) nutritional information and advice, and a follow-up session by a nurse. CG group received usual care.  *eHealth features*  A set-up box connected to participant’s television with Internet connection | | *Primary outcomes*   - Nutritional status measured by the Mini Nutritional Assessment (Short form)   *Secondary outcomes*   - Weight - Diet quality measured by the DHD-FFQ (Dutch Health Diet-FFQ) - Appetite measured by Simplified Nutritional Appetite Questionnaire - Level of physical functioning measured by Katz-15 Short Physical Performance Battery - Quality of life measured by MOS 36 short form health survey. | *Primary outcomes*  At 6 months, IG improved nutritional status (*Δ*=1.77; 95 % CI 0.60 to 2.94), compliance with Dutch guidelines for the intake of vegetables (*Δ*=1.27; 95 % CI 0.49 to 2.05), fruit (*Δ*=1·24; 95 % CI 0.60 to 1.88), dietary fiber (*Δ*=1.13; 95 % CI 0.70 to 1.57), protein (*Δ*=1.20; 95 % CI 0.15 to 2.24), and PA (*Δ*=2.13; 95 % CI 0.98 to 3.29). No changes in CG.  *Secondary outcomes*  No changes in other outcomes. No changes in CG group. |
| Ventura Marra et al (2019), United States ^69^. | | | | | |
| *Study Design* | *Participant Information* | *Intervention description* | | *Outcome measures* | *Results* |
| *Design*  2-group pilot RCT    *Group Info*   - IG, *n* = 29 - CG, *n* = 30   *Duration*  3 months  *Measurement points*  Baseline, 6, 12 weeks  *Attrition rate*  5% | *N* = 59    *Sample*  Men with chronic diseases related to obesity  *Mean age*   - IG = 58.6 (8.1) - CG = 59.3 (7.4)   *Gender distribution*  100% male | *Intervention*  All participants were educated on dietary health topics to improve their diet. They were asked to self-monitor their weight, and dietary behaviours (fruit and vegetable intake, dietary health goals). IG received additional telephone health coaching and videoconferencing sessions to discuss topics on goal setting, weight change, and overcoming barriers to poor dietary habits.  *eHealth features*  Telephone calls, and videoconference | | *Primary outcomes*   - Weight     *Secondary outcomes*   - Body fat - Waist circumference - Energy intake (kcal) - Dietary quality | *Primary outcomes*  At 12 weeks, both IG (mean change = 8.3kg, *P*<.0001) and CG (mean change=5kg, *P*<.0001) significantly reduced their weight compared to baseline. However, no between-group differences were found at 12 weeks (relative *P*=.085).  *Secondary outcomes*  At 12 weeks, both groups significantly reduced body fat (by 2.5% in IG and 1.9% in CG), waist circumference (by 6.8cm in IG, 4.5 cm in CG) and energy intake (by 600.2kcal in IG, 445kcal in CG), and improved diet quality (by score of 20.3 in IG, 12.8 in CG) compared to baseline (all *P*<.0001). There were no between-group differences on these measures at 12 weeks (all *P*>.05). |
| Waki et al (2014), Japan ^40^. | | | | | |
| *Study Design* | *Participant Information* | *Intervention description* | | *Outcome measures* | *Results* |
| *Design*  2-group RCT    *Group Info*   - IG, *n* = 27 - CG, *n* = 27   *Duration*  3 months  *Measurement points*  Baseline, 3 months  *Attrition rate*  18% | *N* = 54    *Sample*  Patients with diabetes mellitus (Type 2)  *Mean age*   - IG = 57.1 (10.2) - CG = 57.4 (9.4)   *Gender distribution*   - IG = 26% female - CG = 22% female | *Intervention*  IG recorded their dietary intake, and input health data, such as weight, step count, BP, into the app. Health data will be evaluated and IG are able to receive feedback from health professionals through the app.  *eHealth features*  Mobile application, DialBetics, which included 4 modules for data transmission, data evaluation, communication, and dietary evaluation. | | *Primary outcomes*   - HbA1c level   *Secondary outcomes*   - Fasting blood glucose (FBG) level - BMI - Cholesterol level - BP level - Diabetes self-management (Diet and Exercise) | *Primary outcomes*  In IG, HbA1c level decreased at 3 months (mean=-0.4) compared to an increase in CG (mean =0.1), *P*=.015.  *Secondary outcomes*  FBG levels in IG decreased at 3 months (mean=-5.5) compared to an increase in CG (mean=16.9), *P*=.019. No significant changes and differences within and between groups for all other outcomes. |
| Wayne et al (2015), Canada ^70^. | | | | | |
| *Study Design* | *Participant Information* | *Intervention description* | | *Outcome measures* | *Results* |
| *Design*  2-group RCT    *Group Info*   - IG, *n* = 67 - CG, *n* = 64   *Duration*  6 months  *Measurement points*  Baseline, 3, 6 months  *Attrition rate*  26% | *N* = 131    *Sample*  Patients with diabetes mellitus (Type 2)  *Mean age*   - IG = 53.1 (10.9) - CG = 53.3 (11.9)     *Gender distribution*   - IG = 65% female (31) - CG = 80% female (39) | *Intervention*  Participants from both IG and CG work together with health coaches to set personalised health goals and track their progress. IG received an additional mobile phone monitoring support that allowed them to self-monitor their health, enter daily food intake, and PA levels. They were able to contact their health coach at any time using the mobile phone.  *eHealth features*  Mobile application | | *Primary outcomes*   - HbA1c level   *Secondary outcomes*   - Weight - BMI - Waist circumference - Satisfaction with life - Hospital Anxiety and Depression scale (HADS) - Positive and negative affect (PANAS) - Health survey scores (SF-12) | *Primary outcomes*  Both IG (mean change = -.82, 95% CI: .46 to 1.17, *P*<.001) and CG (mean change = -.76, 95% CI: .41 to 1.11, *P*<.001) significantly reduced hbA1c levels at 6 months compared with baseline.  However, there were no between-group differences in hbA1c levels at 6 months (*P*=.48).    *Secondary outcomes*  Compared with baseline, IG significantly reduced weight (mean change=-1.22kg, 95% CI:.35 to 2.08, *P*=.006) and waist circumference (mean change=2.23cm, 95% CI:.53 to 3.93, *P*=.01) at 6 months, and CG had no change. There were no changes to BMI in both groups. Both groups improved their satisfaction with life, and certain subscales of the PANAS, HADS, and SF-12. |
| Whitelock et al (2019), UK ^101^. | | | | | |
| *Study Design* | *Participant Information* | | *Intervention description* | *Outcome measures* | *Results* |
| *Design*  2-group RCT  *Group Info*   - IG,  *n* = 53 - CG*, n* = 54   *Duration*  8 weeks    *Measurement points*  Baseline, 4 weeks, 8 weeks    *Attrition rate*  At 4 weeks 20%, at 8 weeks 21% | *N* = 107  *Sample*  Overweight adults  *Mean age*   - IG = 42.8 (10.5) - CG = 44.5 (10.7)     *Gender distribution*   - IG = 77.4% female - CG = 70.4% female | | *Intervention*  IG used a mobile app to record and take pictures of their meal. The app sent notification reminders for users to review their past recordings before their mealtimes. Dietary information is provided in the app. CG received standard care.  *eHealth features*  Mobile application. | *Primary outcomes*   - Weight - Energy intake (kcal)   *Secondary outcomes*   - Body fat (%) | *Primary outcomes*  At 8 weeks, there were no significant group differences in weight reduction between IG and CG [mean difference=-0.1kg (95% CI: -1.6 to 1.3), *P*=.89]. There were no group differences in energy intake [mean difference = 74.9 kcal (95% CI:-273.4 to 423.3) *P*=.67], and no group difference in body fat change [mean difference = 0.1% (95% CI: (-0.7 to 0.9), *P*=.81]. |
| Zhou et al (2016), China ^41^. | | | | | |
| *Study Design* | *Participant Information* | *Intervention description* | | *Outcome measures* | *Results* |
| *Design*  2-group RCT  *Group Info*   - IG, *n* = 50 - CG, *n* = 50   *Duration*  3 months  *Measurement points*  Baseline, 3 months  *Attrition rate*  0% | *N* = 100  *Sample*  Patients with diabetes mellitus (Type 1 and 2)  *Mean age*   - IG = 55 (13.1) - CG = 53.5 (12.4)   *Gender distribution*   - IG = 40% female - CG = 46% female | *Intervention*  IG had access to information relating to diabetes management, such as diet and exercise. They entered self-care data such as dietary intake and blood glucose values. They are able to communicate with clinicians through Welltang. Clinicians provided feedback and help IG set goals and personalised medication regimens.  CG received usual care.  *eHealth features*  Mobile application for diabetes management, Welltang. It has a database for diabetes management information, and diabetes knowledge. | | *Primary outcomes*   - HbA1c level   *Secondary outcomes*   - FBG level - Low-density lipoprotein cholesterol (LDL-C) - Weight - BP - Hypoglycemic events - Diabetes knowledge - Self-care behaviours | *Primary outcomes*  Compared to baseline, both groups decreased HbA1c levels during follow-up (*P*<.001). IG had greater decrease in HbA1c levels compared to CG (mean=1.95 in IG vs mean=.79 in CG, *P*<.001)  *Secondary outcomes*  Compared to baseline, both groups decreased FBG levels, and improved diabetes knowledge and self-care scores during follow-up (all *P*<.001). IG had greater decrease in FBG levels compared to CG (mean=1.90 in IG vs mean=.96 in CG, *P*<.001), and greater improvements in diabetes knowledge (mean=14.3 in IG vs mean=10 in CG, *P*<.001), and self-care scores (mean=15.8 in IG vs mean=10.2 in CG, *P*<.001).  No changes in weight and LDL-C levels at follow-up for both groups. No difference in hypoglycemic events between both groups. |
