# Supplementary material for: Effectiveness of eHealth Nutritional Interventions for Middle-Aged and Older Adults: Systematic Review and Meta-analysis
Source: J Med Internet Res. 2021 May 17;23(5):e15649. doi: 10.2196/15649 (PMC8167617; doi:10.2196/15649)
Supplement: Multimedia Appendix 8 [file jmir_v23i5e15649_app8.docx]

Table S1. Summary of meta-analysis results.

| Anthropometric outcomes | | | | | | |
| --- | --- | --- | --- | --- | --- | --- |
| Outcome | Number of studies | Heterogeneity | | Mean difference | Overall effect | |
|  |  | I^2^ (%) | p-value | MD (95% CI) | Z-value | p-value |
| Weight, kg | 20 | 97 | <.001 | -2.31 (-2.78, -1.84) | 9.64 | <.001 |
| BMI | 16 | 93 | <.001 | -0.68 (-0.98, -0.39) | 4.62 | <.001 |
| Waist circumference, cm | 9 | 91 | <.001 | -2.04 (-3.24, -0.84) | 3.33 | <.001 |
| Clinical outcomes | | | | | | |
| LDL-cholesterol | 3 | 100 | <.001 | -4.41 (-4.52, -4.29) | 72.65 | <.001 |
| Systolic blood pressure | 5 | 74 | 0.002 | -1.67 (-1.77, -1.56) | 30.15 | <.001 |
| Fasting blood glucose | 7 | 79 | <.001 | -0.34 (-0.86, 0.17) | 1.30 | 0.19 |
| hbA1c level, mmol/mol | 12 | 99 | <.001 | -2.12 (-3.01, -1.23) | 4.67 | <.001 |
| Body fat | 5 | 72 | 0.007 | -0.63 (-1.71, 0.45) | 1.14 | 0.26 |
| Triglyceride level | 4 | 87 | <.001 | -1.25 (-2.90, 0.41) | 1.48 | 0.14 |
| Framingham risk | 4 | 95 | <.001 | -1.14 (-3.35, 1.07) | 1.01 | 0.31 |
| Behavioural outcomes | | | | | | |
| Calorie intake, kcal | 5 | 9 | 0.36 | -0.13 (-0.4, 0.13) | 0.98 | 0.33 |
| Fruit and vegetable consumption | 2 | 71 | 0.005 | 0.4 (0.09, 0.7) | 2.52 | 0.01 |

Figure S1. Effect of eHealth interventions on weight (kg).

Figure S2. Effect of eHealth interventions on BMI.

Figure S3. Effect of eHealth interventions on waist circumference (cm).

Figure S4. Effect of eHealth interventions on LDL-cholesterol.

Figure S5. Effect of eHealth interventions on systolic blood pressure.

Figure S6. Effect of eHealth interventions on fasting blood glucose.

Figure S7. Effect of eHealth interventions on hbA1c level (mmol/mol).

Figure S8. Effect of eHealth interventions on body fat.

Figure S9. Effect of eHealth interventions on triglyceride level.

Figure S10. Effect of eHealth interventions on Framingham risk.

Figure S11. Effect of eHealth interventions on calorie intake (kcal).

Figure S12. Effect of eHealth interventions on fruit and vegetable consumption (FVC).
